# Site-Specific Expression Pattern of PIWI-Interacting RNA in Skin and Oral Mucosal Wound Healing

**DOI:** 10.3390/ijms21020521

**Published:** 2020-01-14

**Authors:** Lin Chen, Zujian Chen, Alyne Simões, Xinming Wu, Yang Dai, Luisa A. DiPietro, Xiaofeng Zhou

**Affiliations:** 1 Center for Wound Healing and Tissue Regeneration, College of Dentistry, University of Illinois at Chicago, Chicago, IL 60612, USA; chenlin@uic.edu (L.C.); lysimoes@usp.br (A.S.); 2 Department of Periodontics, College of Dentistry, University of Illinois at Chicago, Chicago, IL 60612, USA; chenzuj@uic.edu (Z.C.); xwu84@uic.edu (X.W.); 3 Oral Biology Laboratory, Department of Biomaterials and Oral Biology, School of Dentistry, University of São Paulo, São Paulo, SP 05508-000, Brazil; 4 Department of Bioengineering, College of Engineering, University of Illinois at Chicago, Chicago, IL 60607, USA; yangdai@uic.edu; 5 Graduate College, University of Illinois at Chicago, Chicago, IL 60607, USA; 6 UIC Cancer Center, University of Illinois at Chicago, Chicago, IL 60612, USA

**Keywords:** wound healing, piRNA, *PIWI* gene, oral mucosa, skin

## Abstract

The oral mucosa exhibits exceptional healing capability when compared to skin. Recent studies suggest that intrinsic differences in coding genes and regulatory small non-coding RNA (sncRNA) genes (e.g., microRNAs) may underlie the exceptional healing that occurs in the oral mucosa. Here, we investigate the role of a novel class of sncRNA—Piwi-interacting RNA (piRNA)—in the tissue-specific differential response to injury. An abundance of piRNAs was detected in both skin and oral mucosal epithelium during wound healing. The expression of *PIWI* genes (the obligate binding partners of piRNAs) was also detected in skin and oral wound healing. This data suggested that PIWI-piRNA machinery may serve an unknown function in the highly orchestrated wound healing process. Furthermore, unique tissue-specific piRNA profiles were obtained in the skin and oral mucosal epithelium, and substantially more changes in piRNA expression were observed during skin wound healing than oral mucosal wound healing. Thus, we present the first clue suggesting a role of piRNA in wound healing, and provide the first site-specific piRNA profile of skin and oral mucosal wound healing. These results serve as a foundation for the future investigation of the functional contribution(s) of piRNA in wound repair and tissue regeneration.

## 1. Introduction

Impaired cutaneous wound healing affects approximately 6.7 million patients in the U.S., and up to 2% of the population in developed countries suffer from chronic wounds [1]. The incidence is expected to rise at a rate of 2% annually over the next decade, partly brought on by an aging population and increasing rates of diseases and conditions such as diabetes, obesity, and the late effects of radiation therapy. Oral mucosal wound healing has long been considered an optimal wound resolution and is characterized by rapid and scarless healing. Recent comparative studies focusing on differential molecular characterization of the skin and oral mucosa have revealed that the intrinsic molecular differences in skin and oral mucosa contribute to the divergent wound healing outcomes [2,3,4]. This novel approach defines critical molecular regulators involved in accelerated wound healing and allows the harnessing of regenerative healing capabilities in oral mucosa to treat deficient healing processes.

Wound healing is a complex process that consists of four overlapping phases: Hemostasis, Inflammatory, Proliferative, and Maturation. This highly orchestrated process is regulated by numerous genetic and epigenetic regulators, including regulatory small non-coding RNAs (sncRNA) such as microRNA and piwi-interacting RNA (piRNA). While the role of microRNA in wound healing has been studied extensively [5], the role of piRNA in wound healing is not known. piRNAs are a class of sncRNAs discovered recently (in 2006). They are slightly longer (25–32 nt) than microRNAs (18–25 nt) and belong to the largest class of sncRNAs in animal cells [6,7,8,9]. While initially discovered as a class of germline-specific sncRNAs that mediate silencing of transposons in germline cells [10,11], piRNAs also target non-transposable elements, such as protein-coding genes, and modulate their expression not only in germline cells but also in somatic cells by mechanisms similar to that of microRNAs [12,13]. The functional relevance of piRNAs in somatic cells has just begun to be recognized. Most of the relevant knowledge gained in somatic cells are from recent cancer studies [14]. Studies on piRNA in wound healing and tissue regeneration are very limited, including an early study implicating the involvement of piRNA in limb regeneration in salamanders [15,16], and a recent report suggesting that piRNA-mediated post-transcriptional gene silencing is involved in neuronal regeneration in C. elegans [17]. Similar to microRNAs, piRNAs also interact with the Argonaute (AGO) family of RNA-binding proteins to guide target-specific gene regulation, specifically the PIWI (P-element Induced WImpy testis) subclass of AGO proteins. Piwi proteins are highly conserved RNA-binding proteins and are present in both plants and animals. Four *PIWI* genes have been identified in the human genome (hPIWIL1, 2, 3, 4), and 3 *PIWI* genes were identified in mice (*mPIWIL1*, 2, 4). In this study, we explored the potential relevance of PIWI-piRNA machinery in the skin and oral mucosal wound healing and assessed its role in differential responses to injury. Our results provide the first clue that piRNA may play an important role in regulating wound healing.

## 2. Results

### 2.1. PIWI Genes Expression in Skin and Oral Mucosal Wounds

**Figure 1:**
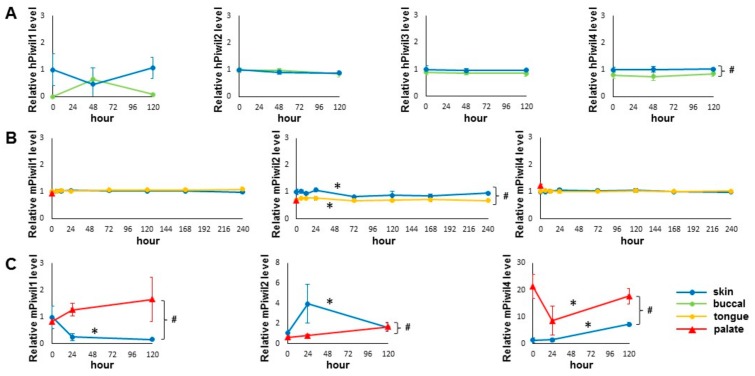
Detection of PIWI gene expression in skin and oral mucosal wounds. (**A**) The relative expression values of human *PIWI* genes (*PIWIL1*, *PIWIL2*, *PIWIL3*, *PIWIL4*) during skin and buccal mucosal wound healing time course (0 h, 48 h, and 120 h) were extracted from the existing RNA-seq gene expression dataset (GSE97615), as described in Materials and Methods. (**B**) The relative expression values of mouse *PIWI* genes (*PIWIL1*, *PIWIL2*, *PIWIL4*) during skin and tongue wound healing time course (0 h, 6 h, 12 h, 24 h, 72 h, 120 h, 168 h, and 240 h) and unwounded mouse palate mucosa (baseline) were extracted from the existing microarray gene expression datasets (GSE23006 and GSE56135), as described in Materials and Methods. (**C**) The expression levels of mouse *PIWI* genes (*PIWIL1*, *PIWIL2*, *PIWIL4*) were assessed by quantitative real-time PCR on tissue samples procured from a paired murine skin and oral mucosal wound healing model, at 0 h, 24 h and 120 h post-wounding (*n* = 3 for both groups). * indicates one-way ANOVA test *p* < 0.05 (within time-course comparison); ^#^ indicates two-way ANOVA test *p* < 0.05 (between time-courses comparison).

The expression of *PIWI* genes in the skin and oral mucosal wounds was evaluated by examining the existing transcriptome profiling datasets. Included in this analysis were two recent time course experiments on paired human skin and buccal mucosal wounds and on paired mouse skin and tongue wounds, respectively [2,4]. As shown in Figure 1A, the mRNA of all four human *PIWI* genes (*hPIWIL1*, 2, 3, 4) were present in human skin and buccal mucosa samples. A statistically significant difference in hPIWIL4 mRNA levels was observed between the skin and buccal mucosal epithelium. The mRNA expression levels of all other *PIWI* genes were relatively consistent during the time course of both skin and buccal mucosal wound healing in humans. A similar observation was made in mice (Figure 1B), where all three mouse PIWI mRNAs (mPIWIL1, 2, 4) were present in mouse skin and tongue. A statistically significant difference in mPIWIL2 mRNA levels was observed between skin and tongue, and statistically significant changes in mPIWIL2 mRNA levels were observed in both skin and tongue wound healing time courses. The mRNA levels of other *PIWI* genes (*mPIWIL1* and *mPIWIL4*) are relatively consistent during the time course of both skin and tongue wound healing in mice. The data on mouse *PIWI* genes from the unwounded hard palate mucosa (baseline) [18] were also included in Figure 1B, and their mRNA levels were comparable with those observed in the tongue. 

We further examined the expression of *PIWI* genes by RT qPCR in a paired murine skin and hard palate wound healing model. As shown in Figure 1C, the expression of all three mouse *PIWI* genes was detectible by qPCR assays. Tissue-specific statistically significant differences between skin and palate were detected in all three mouse *PIWI* genes. Statistically significant expression changes of all three mouse *PIWI* genes were detected by qPCR during skin wound healing. A statistically significant change was observed only in *mPIWIL4* expression during palate wound healing. Taken together, while variation in *PIWI* gene expression was observed among different studies on skin wounds and oral mucosa wounds at different sites (buccal mucosa, tongue, palate), the expression of *PIWI* genes were consistently detected in both skin and oral mucosa. 

### 2.2. High Levels of piRNA Presented in Skin and Oral Mucosa Epithelium

**Figure 2:**
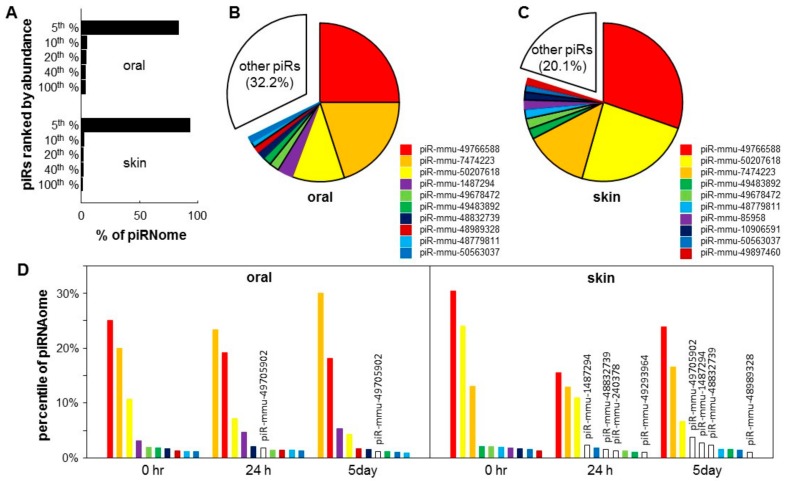
Piwi-interacting RNA (piRNA) landscape of skin and oral mucosa. Baseline piRNA profiles were obtained on the normal mouse skin and oral mucosa (hard palate) (*n* = 3 for both groups). (**A**) The piRNA landscape is presented as histograms of the percentage of total piRNA contents (piRNAome) vs. piRNA species grouped by their abundance (top 5%, 6–10%, 11–20%, 21–40%, and 41–100%). Pie charts illustrated that the top 10 most abundant piRNAs account for 67.8% and 79.9% of the piRNAome in the oral mucosa (palate) (**B**) and skin (**C**). (**D**) Injury induced changes in the levels of the top 10 most abundant piRNAs in the skin and oral mucosa during the wound healing time course (0 h, 24 h and 5 days), expressed as a percentage of the piRNAome.

The expression of piRNA genes in the normal murine uninjured skin and oral mucosal epithelium (baseline) was explored by re-analyzing of our previous small-RNA seq data [3], and mapping to the piRNA database. A total of 20,862,850 and 3,769,588 sequence reads were mapped to 1084 and 1103 unique piRNAs for skin and oral mucosa, respectively. The top 5% most abundant piRNA species account for 83.6% and 93.4% of all piRNAs in oral mucosa and skin epithelium, respectively (Figure 2A). The top 10 most abundant piRNAs in the skin and oral mucosa account for 67.8% and 79.9% of all piRNAs, respectively (Figure 2B,C). 

### 2.3. Tissue-Specific piRNome Landscape Changes during the Wound Healing 

To examine the piRNA landscape changes during wound healing, we calculated the % of the piRNome represented by each of the top 10 most abundant piRNAs during the time course of the skin and oral mucosa wound healing (0 h, 24 h and 5 day). As shown in Figure 2D, for oral mucosa, while the expression levels changed after wounding, the composition of the top 10 most abundant piRNAs remained relatively constant. Only 1 out of the top 10 most abundant piRNAs was replaced by a new piRNA (piR-mmu-49705902 appeared in the top 10 list at 24 h and 5 days post-wounding). In contrast, for skin, after wounding, four out of the top 10 most abundant piRNAs were replaced by new piRNAs (piR-mmu-1487294, piR-mmu-48832739, piR-mmu-240378 and piR-mmu-49293964 appeared in the top 10 list at 24 h and 5 days post-wounding). The list of differentially expressed piRNAs during skin and oral mucosal wound healing is shown in Figure S1 and Table S1.

**Figure 3:**
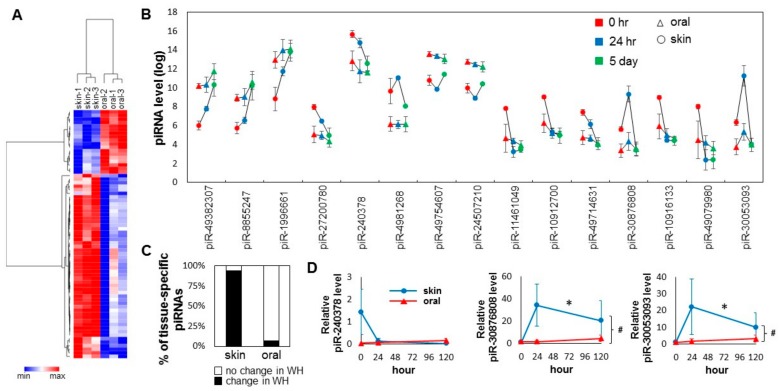
Injury induced changes in the tissue-specific piRNA pattern during skin and oral mucosa wound healing. **(A**) Tissue-specific piRNA signatures (presented as heatmap) of the baseline skin and oral mucosa (palate) epithelium were established by their differential expression patterns (70 piRNAs, BH adjusted *p* < 0.05). (**B**) Changes in the levels of the top 15 tissue-specific piRNAs between the skin and oral mucosa during wound healing time course (* *p* < 0.05). (**C**) The percentage of those piRNAs that were identified as differentially expressed in normal uninjured tissue that also exhibit expressional changes during skin and oral mucosal wound healing (WH). The difference is statistically significant (chi-square test *p* = 0.0001). (**D**) The relative levels of piR-mmu-240378, piR-mmu-30876808, and piR-mmu-30053093 were assessed by TaqMan assay-based real-time PCR assays on samples procured from the paired murine skin and oral mucosa (palate) wound healing model (at 0 h, 24 h and 5 days post-wounding, *n* ≥ 3). * indicates one-way ANOVA test *p* < 0.05 (within time-course comparison); ^#^ indicates two-way ANOVA test *p* < 0.05 (between time-courses comparison).

A total of 70 tissue-specific differentially expressed piRNAs were identified (Figure 3A, Table S2). Of these 70 piRNAs, 18 are oral-specific (high in oral mucosa and low in skin), and 52 are skin-specific (high in skin and low in oral mucosa). Bioinformatic analysis (target prediction and pathway analysis) revealed that different biological pathways are targeted by oral-specific and skin-specific piRNAs (Table 1 and Table S3). As shown in Figure 3C, of the 70-baseline tissue-specific differentially expressed piRNAs, 66 (94.3%) were differentially expressed during skin wound healing, and only five (5.7%) were differentially expressed during oral mucosa wound healing (chi-square test *p* < 0.0001). For the top 15 baseline tissue-specific differentially expressed piRNAs, all of them showed statistically significant changes during skin wound healing, and only one (piR-30053093) of them was changed during oral mucosal wound healing (Figure 3B). To confirm the differential expression of selected piRNAs (piR-240378, piR-30876808, piR-30053093), TaqMan assay-based validation was performed in additional wound tissue samples (*n* >= 3). As shown in Figure 3D, statistically significant differential expression of piR-30876808, piR-30053093 were confirmed during skin wound healing (one-way ANOVA *p*-value < 0.05), and between the skin and oral mucosal wound healing time courses (two-way ANOVA *p*-value < 0.05). While apparent differences in piR-240378 level were observed during the skin wound healing and between the skin and oral mucosal wound healing time courses, these differences were not statistically significant.

## 3. Discussion

piRNAs are the largest class of regulatory sncRNAs expressed in animal cells. Extensive studies on the roles of piRNAs in the germline and gonads revealed that piRNAs exert their functions by forming RNA-induced silencing complex (RISC) with PIWI proteins [10,11]. While initially, piRNAs were thought to regulate transposons exclusively in germline cells, recent studies demonstrated that piRNAs also participate in gene regulation in somatic cells [19,20,21]. Studies on lower organisms (salamanders and C. elegans) suggested that piRNAs play an important role in tissue regeneration [15,16,17]. Intriguingly the PIWI-piRNA system has been suggested to regulate essential cellular functions of relevance to the repair of somatic tissues, including cell proliferation, viability, cell invasion and migration [22]. However, most of the newly gained knowledge of the functional role of piRNA is based on cancer studies, including studies on skin cancers [23,24] and oral cancers [25,26,27,28]. It is tempting to hypothesize that piRNAs play a role in wound healing. We observed high levels of piRNAs in the skin and oral mucosa during the wound healing time course. Similarly, the expression of *PIWI* genes (obligate binding partners of piRNAs) was also observed in skin and oral mucosal wounds, suggesting that PIWI-piRNA machinery is an integral component of the highly orchestrated wound healing process. The observed tissue-specific piRNA and PIWI gene pattern between the skin and oral mucosa further suggested that the differential regulation of PIWI-piRNA machinery in skin and oral mucosal wounds may contribute to the site-specific healing response.

In this study, we present the first tissue-specific piRNA profiles in corresponding skin and oral mucosal wounds. Together with our previous study that established the tissue-specific transcriptome and microRNAome of matching skin and mucosal wounds [2,3], we demonstrated striking differences in the transcribed genome (transcriptome, microRNAome, and piRNAome) of oral mucosal and skin wounds. Along with studies by others [4,29], our results suggest that the intrinsic differences in the genetic and epigenetic responses to injury in the skin and mucosa contribute to the divergent wound healing outcomes. One of our important observations is that more piRNA genes were differentially expressed in skin wounds than in oral mucosal wounds. This is in agreement with our previous observations showing that nearly twice as many protein-coding genes and six times as many microRNA genes are differentially expressed in the skin wounds than in mucosal wounds [2,3]. Collectively, these observations imply that, as compared to the skin, the oral mucosa has an intrinsic genetic/epigenetic controlling program that makes it be highly adaptable to the accelerated healing upon injury. These differences at the genomic level underlie the functional observation that oral mucosa turns over more rapidly than skin [30,31]. This rapid turnover of mucosal cells in the oral cavity is crucial to support the frequent repair of small injuries and constant shedding of the microbiome. A possible explanation for the apparent differences in the transcribed genome (both transcriptome and sncRNAome) could be that the mucosa, being “preactivated”, would not require a significant change in the expression of genes during the healing process [4]. An alternative explanation for the observed larger changes in skin wounds might be that some of the highly expressed skin-specific genes (at uninjured baseline situation) may function as suppressors of injury response. These suppressors need to be downregulated upon injury so that skin wounds can achieve a proper healing outcome. This is supported by our bioinformatic based prediction suggesting that the skin-specific piRNAs target a number of proliferation pathways (such as MAPK and Ras signaling pathways). Similar observations were made at the microRNA level. For example, members of the miR-10 and miR-99 families are highly expressed in skin, and they suppress the skin wound healing process [3,32,33]. To activate the injury-induced genomic response program, these suppressor genes (e.g., miR-10/99 family members) need to be turned off in the skin, which adds complexity and extra steps, compared to the oral mucosa. 

The feasibility of microRNA-based application in wound healing treatment is currently under intense investigation. We have recently demonstrated that we were able to accelerate skin wound closure by manipulating specific microRNAs (e.g., miR-21, miR-10b, and miR-31) [3,34]. Similar results showing the feasibility and effectiveness of the microRNA-based approach to regulate various aspects of wound healing (e.g., inflammatory response, re-epithelialization, and angiogenesis) were also reported by other groups [35,36,37,38,39]. Since piRNA and microRNA share many structural and functional similarities, it is logical to hypothesize that specific piRNAs can also serve as therapeutic targets for wound healing treatment. With proper optimization, this strategy may be adapted to develop a novel piRNA-based approach for wound healing treatment.

While the role of piRNA in somatic cells is currently under intense investigation, several technical difficulties hinder the progress of research in this area. For example, currently, the bioinformatics tool for predicting non-transposon piRNA targets is very limited. In this study, we adapted an established algorithm developed for microRNA target prediction (microT method from DIANA-LAB [40,41]) for the prediction of piRNA targets. Although it is accepted that piRNAs and microRNAs utilize similar mechanisms to guide target-specific gene regulation (based on RNA-induced silencing complex, or RISC), elusive differences in sequence features guiding the binding to their mRNA targets have been observed (e.g., length of seed region and allowable mismatches) [42,43]. As a consequence, our attempt to utilize microT for piRNA target prediction will inevitably lead to false-positive results (even with stringent statistical settings). Thus, additional validation studies (e.g., reporter gene assay and ribonucleoprotein-IP assay) and functional assays (based on knockdown and/or overexpression strategies) are required to fully validate our predicted piRNA target genes. 

In summary, we presented here the first piRNA profile in wound healing and provided the first clue of the potential involvement of piRNA in wound healing regulation. Our results, together with our previous findings [2,3], clearly demonstrated that intrinsic genetic and epigenetic differences in oral mucosa contribute to its accelerated wound healing. This study advances our understanding of the complexity of the wound healing process and provides a foundation for developing novel sncRNA-based therapeutic targets to promote cutaneous wound closure and/or prevent chronic wounds.

## 4. Materials and Methods

### 4.1. Animals and Wound Models

The animal protocol was approved by the University of Illinois at Chicago Office of Animal Care and Institutional Biosafety Committee (ACC 17-219, approved on 01/18/2018), and all experimental procedures were performed in accordance with the National Institutes of Health (NIH) Guide for the Care and Use of Laboratory Animals. Female Balb/c mice (8–10-week-old, Jackson Laboratory, Bar Harbor, ME) were housed in a temperature-controlled environment (22–24 °C) on a 12 h/12 h light-dark cycle, and were provided with food and water ad libitum. For wound healing studies in skin and oral mucosa, the previously established mouse incisional skin and oral mucosal wound healing models were adapted with minor modifications [3,44,45]. In brief, mice (total *n* = 18) were randomly allocated to three groups (skin wound, oral wound, and control groups). Prior to all procedures, mice were anesthetized with ketamine (100 mg/kg) and xylazine (5 mg/kg), administered intra-peritoneally. To create the skin wounds, mice (*n* = 6) were shaved and cleaned thoroughly with 70% isopropyl alcohol. Using a pair of scissors, two 1 cm long full-thickness incisional wounds were created on the dorsal skin of mice (one on each side of the midline). The animals were euthanized at 24 h and 5 days post-wounding (*n* = 3 per time-point). The tissue specimens were harvested and immediately placed in RNAlater (Sigma-Aldrich, St. Louis, MO, USA) and stored at −20 °C until RNA isolation. For the oral wound group (*n* = 6), three 0.5 cm incisional wounds were made on the anterior of the hard palate using a scalpel blade. The animals were euthanized at 24 h and 5 days post-wounding (*n* = 3 per time-point). The tissue specimens were harvested and immediately placed in RNAlater (Sigma-Aldrich, St. Louis, MO, USA) and stored at −20 °C until RNA isolation. Unwounded skin and hard palate samples (baseline, *n* = 3 per group) were harvested from the control animals. 

### 4.2. Real-Time PCR

The mRNA levels of mouse *PIWI* genes were determined by quantitative two-step RT-PCR assay with gene-specific primer sets for *PIWIL1*, *PIWIL2* (OriGene Technologies, Rockville, MD, USA) and *PIWIL4* (Real-Time Primers, Elkins Park, PA, USA) as described before [32]. The expression levels of piRNA genes were determined by custom-designed TaqMan assays produced using the Custom TaqMan Small RNA Assay service from Applied Biosystems (Foster City, CA, USA) (piR-mmu-240378 (Assay ID: CTRWEUD, Context Sequence: CCCTGGTGGTCTAGTGGTTAGGATTCGGC); piR-mmu-30876808 (Assay ID: CTTZ9EA, Context Sequence: AAATGGATTTTTGGAACTAGG); and piR-mmu-30053093 (Assay ID: CTU62X7, Context Sequence: AAATGGATTTTTGGAAGTAGG)). The relative mRNA and piRNA levels were computed using the 2-delta delta Ct analysis method [46], where *β*-actin and U6 were used as internal controls, respectively.

### 4.3. Bioinformatic Analysis

For evaluating the expression of *PIWI* genes in skin and oral musical wounds, two existing transcriptome profiling datasets were used. The first was an RNA-seq gene expression dataset on paired human skin and buccal mucosal wound healing [4]. The expression profile dataset was downloaded from the Gene Expression Omnibus (GEO, accession # GSE97615). Data analysis was performed with CyberT [47], using a Variance Stabilizing Normalization (VSN) method [48,49], and the normalized expression values of human *PIWI* genes (*PIWIL1*, *PIWIL2*, *PIWIL3*, *PIWIL4*) were extracted and presented as relative values to skin at the 0 h time point (baseline). We chose CyberT due to its capability of handling both microarray and RNA-seq datasets, and its documented superiority in handling a small number of replicates [50,51]. The second is a microarray dataset on a paired mouse skin and tongue wound healing model, as well as unwounded palate (baseline) [2,18]. The expression profile dataset was downloaded from GEO (accession # GSE23006 and GSE56135). Data analysis and normalization were performed with GEO2R [52], and the normalized gene expression values of mouse *PIWI* genes (*PIWIL1*, *PIWIL2*, *PIWIL4*) were extracted and presented as relative values to skin at the 0 h time point (baseline). 

The previously published small RNA-seq dataset of paired mouse skin and hard palate mucosa wound healing over time (GEO accession # GSE121996) was processed, mapped and analyzed using a proprietary pipeline script, ACGT101-miR v4.2g (LC Sciences, Houston, TX, USA) as described previously [3], where piR mouse v1.0 database (www.regulatoryrna.org/database/piRNA/) was used for mapping. Normalization and differential expression analysis were performed with CyberT [47], using Variance Stabilizing Normalization (VSN) method [48,49]. The prediction of piRNA target genes was carried out by utilizing a MapReduce-based implementation of the microT target prediction method (MR-microT, beta version) from DIANA-LAB (http://diana.imis.athena-innovation.gr/DianaTools/, [40,41]), with a stringent aggregated microT score (≥ 0.9). The predicted piRNA targets were analyzed with the Database for Annotation, Visualization, and Integrated Discovery (DAVID, v6.8) [53,54] to unveil their involvement in biological processes and pathways.

Student’s *t*-test was used for between-group comparisons, one-way ANOVA was used for time-course data analysis, and two-way ANOVA was used for between two time-course data comparisons. The Benjamini-Hochberg adjusted *p*-values were computed for multiple hypothesis testing. A value of *p* < 0.05 was considered statistically significant unless stated otherwise. 

## Figures and Tables

**Table 1:** Bioinformatic prediction of molecular pathways regulated by tissue-specific differentially expressed piRNAs ^a^.

KEGG_PATHWAY ^b^	Pathway ID	Genes Targeted	*p*-Value
**Oral-Specific piRNAs Targeted Pathways:**			
mRNA surveillance pathway	mmu03015	10	1.92 × 10^−5^
Oocyte meiosis	mmu04114	8	0.001947
Dopaminergic synapse	mmu04728	8	0.005301
AMPK signaling pathway	mmu04152	7	0.015899
RNA transport	mmu03013	8	0.018233
**Skin-Specific piRNAs Targeted Pathways:**			
Renal cell carcinoma	mmu05211	13	2.58 × 10^−5^
T cell receptor signaling pathway	mmu04660	14	3.44 × 10^−4^
Axon guidance	mmu04360	14	0.003386
MAPK signaling pathway	mmu04010	21	0.005309
Ras signaling pathway	mmu04014	19	0.009136

**^a^** 70 tissue-specific differentially expressed piRNAs (including 18-oral specific and 52 skin-specific piRNAs, Table S2) were used for the analysis. The piRNA target gene prediction was performed with MR-microT (aggregated score ≥ 0.9), and the predicted target genes were presented in Tables S4 and S5; **^b^** The pathway analysis was performed with DAVID (v6.8). Top five pathways targeted by oral-specific and skin-specific piRNAs were presented in the table. A complete list of pathways regulated by 70 tissue-specific differentially expressed piRNAs is presented in Table S3.

## Supplementary Materials

Supplementary materials can be found at https://www.mdpi.com/1422-0067/21/2/521/s1.
