# Supplementary material for: Site-Specific Expression Pattern of PIWI-Interacting RNA in Skin and Oral Mucosal Wound Healing
Source: Int J Mol Sci. 2020 Jan 14;21(2):521. doi: 10.3390/ijms21020521 (PMC7013508; doi:10.3390/ijms21020521)
Supplement: Supplementary file 1 [file ijms-21-00521-s001.pdf]

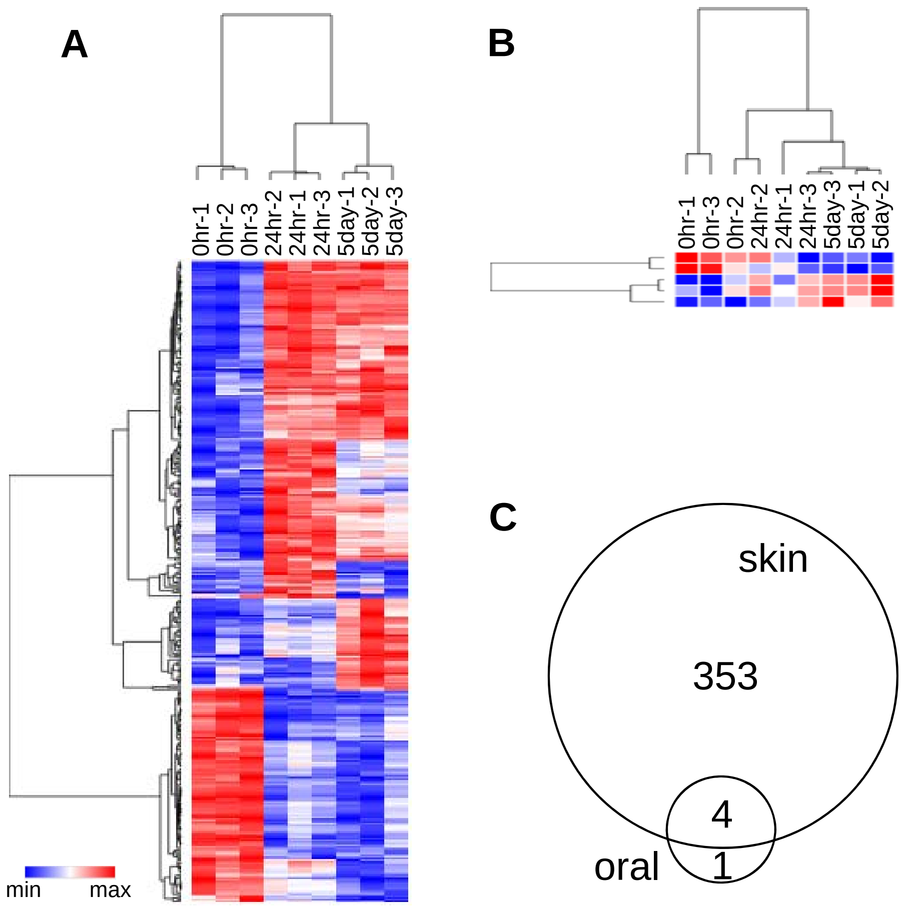


**Supplementary Figure S1: Differentially expressed piRNAs during skin and oral mucosal wound healing.** (**A**) piRNA profiles were obtained on mouse skin and oral mucosal (palate) wound healing time course (0hr, 24 hr, and 5 day). A total of 357 differentially expressed piRNA were identified during skin wound healing (Bonferroni adjusted P value <0.05). See **Supplementary Table 1A** for the full list. (**B**) Five differentially expressed piRNA were identified during oral mucosal wound healing (P value <0.01, list presented in **Supplementary Table 1B**). Note: more stringent statistical cut-off ((Bonferroni adjusted P value) yield 0 differentially expressed piRNA gene. (**C**) Venn diagram illustrates overlaps between differentially expressed piRNAs in skin and oral mucosal wound healing.

**Supplementary Table S1a: Differentially expressed piRNAs in skin wound healing**

| | Mean | | | StDev | | | | |
|---|---|---|---|---|---|---|---|---|
| **piRNA** | 0 hr | 24 hr | 5 day | 0 hr | 24 hr | 5 day | **pVal** | **adj P** |
| piR-mmu-15927330 | 5.418351 | 11.39746 | 10.799 | 0.34576 | 0.253492 | 0.154802 | 6.32E-14 | 6.97E-11 |
| piR-mmu-49559417 | 5.301647 | 10.10777 | 9.816878 | 0.441719 | 0.222335 | 0.032479 | 3.95E-12 | 4.36E-09 |
| piR-mmu-30053093 | 6.32531 | 11.26384 | 4.020902 | 0.280841 | 1.057847 | 0.178798 | 5.21E-12 | 5.76E-09 |
| piR-mmu-29303577 | 5.15005 | 10.47662 | 9.52175 | 0.554877 | 0.26622 | 0.163283 | 1.53E-11 | 1.69E-08 |
| piR-mmu-49254706 | 5.187673 | 10.19644 | 9.622374 | 0.520671 | 0.330378 | 0.191116 | 1.96E-11 | 2.16E-08 |
| piR-mmu-49005170 | 5.415133 | 9.639725 | 9.565967 | 0.411507 | 0.281143 | 0.095657 | 2.01E-11 | 2.22E-08 |
| piR-mmu-33056206 | 5.178179 | 9.864265 | 9.39513 | 0.538986 | 0.283804 | 0.240795 | 2.29E-11 | 2.53E-08 |
| piR-mmu-49117425 | 5.43053 | 9.776696 | 9.379862 | 0.332302 | 0.370967 | 0.418124 | 2.63E-11 | 2.90E-08 |
| piR-mmu-49947745 | 5.15005 | 10.39038 | 9.553774 | 0.554877 | 0.404618 | 0.063169 | 2.77E-11 | 3.05E-08 |
| piR-mmu-30876808 | 5.633238 | 9.351468 | 3.413962 | 0.221386 | 0.842191 | 0.638678 | 2.80E-11 | 3.09E-08 |
| piR-mmu-24697737 | 6.749407 | 13.33618 | 9.219858 | 0.78039 | 0.376817 | 0.664177 | 3.32E-11 | 3.66E-08 |
| piR-mmu-26877685 | 5.825909 | 9.412237 | 10.40902 | 0.295957 | 0.296975 | 0.549284 | 5.09E-11 | 5.62E-08 |
| piR-mmu-51208629 | 5.322489 | 9.674555 | 4.339173 | 0.482649 | 0.476203 | 0.569868 | 5.94E-11 | 6.56E-08 |
| piR-mmu-49214057 | 5.173474 | 9.463311 | 9.688759 | 0.530532 | 0.186272 | 0.203834 | 6.33E-11 | 6.99E-08 |
| piR-mmu-49857560 | 6.096626 | 10.80289 | 8.089564 | 0.305426 | 0.190109 | 0.500752 | 7.57E-11 | 8.36E-08 |
| piR-mmu-28107338 | 5.279592 | 9.686733 | 4.636521 | 0.436702 | 0.597722 | 0.352074 | 8.29E-11 | 9.15E-08 |
| piR-mmu-29851930 | 5.187673 | 8.477601 | 10.01247 | 0.520671 | 0.319898 | 0.305159 | 9.40E-11 | 1.04E-07 |
| piR-mmu-37120680 | 5.61471 | 9.783534 | 9.842512 | 0.257938 | 0.487058 | 0.553785 | 1.07E-10 | 1.18E-07 |
| piR-mmu-49293964 | 8.081628 | 14.49751 | 10.49034 | 1.118888 | 0.318574 | 0.503249 | 1.22E-10 | 1.35E-07 |
| piR-mmu-49342972 | 10.3054 | 5.26409 | 5.567938 | 0.317342 | 0.194286 | 0.552373 | 1.63E-10 | 1.80E-07 |
| piR-mmu-11998481 | 5.306418 | 9.156603 | 8.816 | 0.448088 | 0.068244 | 0.191428 | 1.96E-10 | 2.16E-07 |
| piR-mmu-33436983 | 5.302276 | 9.555314 | 8.642954 | 0.503112 | 0.361598 | 0.236862 | 2.16E-10 | 2.38E-07 |
| piR-mmu-29438338 | 5.164249 | 8.891336 | 9.356356 | 0.54637 | 0.166207 | 0.354669 | 2.42E-10 | 2.67E-07 |
| piR-mmu-29446719 | 5.446596 | 9.023653 | 9.080276 | 0.368554 | 0.442015 | 0.127741 | 2.44E-10 | 2.70E-07 |
| piR-mmu-33225252 | 5.918832 | 10.28668 | 7.585454 | 0.363775 | 0.362888 | 0.064971 | 2.52E-10 | 2.79E-07 |
| piR-mmu-46771645 | 5.879289 | 9.182917 | 9.463217 | 0.139979 | 0.459672 | 0.253782 | 2.61E-10 | 2.88E-07 |
| piR-mmu-31717841 | 6.311648 | 12.04745 | 10.12849 | 0.747336 | 0.530349 | 0.751021 | 2.91E-10 | 3.21E-07 |
| piR-mmu-24890504 | 5.15005 | 9.761486 | 8.754651 | 0.554877 | 0.398959 | 0.209057 | 3.38E-10 | 3.73E-07 |
| piR-mmu-49197585 | 8.442509 | 3.27852 | 3.589529 | 0.294622 | 0.394896 | 0.048466 | 3.40E-10 | 3.76E-07 |
| piR-mmu-48846936 | 5.797893 | 10.29159 | 8.177274 | 0.376319 | 0.355817 | 0.306872 | 3.76E-10 | 4.15E-07 |
| piR-mmu-50223161 | 5.422519 | 9.841802 | 9.600623 | 0.565765 | 0.386174 | 0.805022 | 3.89E-10 | 4.29E-07 |
| piR-mmu-49657255 | 5.869841 | 11.37368 | 5.02339 | 0.189147 | 1.661196 | 0.715557 | 4.37E-10 | 4.83E-07 |
| piR-mmu-48726739 | 5.15005 | 8.89129 | 9.519018 | 0.554877 | 0.315097 | 0.538707 | 5.17E-10 | 5.71E-07 |
| piR-mmu-40290832 | 5.613701 | 9.037247 | 9.057221 | 0.270534 | 0.413085 | 0.402927 | 5.19E-10 | 5.73E-07 |
| piR-mmu-50927440 | 5.395527 | 8.985665 | 9.625324 | 0.494797 | 0.197356 | 0.692051 | 6.23E-10 | 6.88E-07 |

| | | | | | | | | |
|---|---|---|---|---|---|---|---|---|
| piR-mmu-35442814 | 6.280799 | 10.20491 | 10.31227 | 0.210296 | 0.510832 | 0.271543 | 7.61E-10 | 8.40E-07 |
| piR-mmu-49665792 | 5.232933 | 7.595773 | 9.459027 | 0.503115 | 0.154799 | 0.26182 | 8.58E-10 | 9.47E-07 |
| piR-mmu-49940004 | 6.149593 | 10.01617 | 9.962588 | 0.149371 | 0.132921 | 0.805658 | 8.87E-10 | 9.80E-07 |
| piR-mmu-49079980 | 8.016324 | 2.404083 | 2.367978 | 0.225311 | 1.070106 | 0.933203 | 1.09E-09 | 1.20E-06 |
| piR-mmu-3729268 | 6.727558 | 12.34273 | 9.038599 | 0.432931 | 0.396272 | 1.054685 | 1.13E-09 | 1.25E-06 |
| piR-mmu-23866657 | 5.909041 | 8.758412 | 3.731125 | 0.481623 | 0.969402 | 0.29095 | 1.24E-09 | 1.36E-06 |
| piR-mmu-49200681 | 5.517559 | 8.819822 | 11.29521 | 0.558373 | 0.255394 | 1.359045 | 1.31E-09 | 1.45E-06 |
| piR-mmu-10916133 | 9.006139 | 4.435075 | 4.378467 | 0.181429 | 0.191708 | 0.472498 | 1.38E-09 | 1.52E-06 |
| piR-mmu-49442773 | 5.695952 | 9.162211 | 9.759958 | 0.65509 | 0.110523 | 0.693257 | 1.57E-09 | 1.73E-06 |
| piR-mmu-24767632 | 6.246944 | 9.893676 | 9.87215 | 0.670547 | 0.218314 | 0.148024 | 1.60E-09 | 1.77E-06 |
| piR-mmu-49468812 | 5.259046 | 8.890393 | 8.84603 | 0.456434 | 0.374162 | 0.236757 | 1.72E-09 | 1.89E-06 |
| piR-mmu-46411880 | 5.757963 | 9.210236 | 8.870957 | 0.209247 | 0.505991 | 0.188884 | 1.84E-09 | 2.03E-06 |
| piR-mmu-49629011 | 6.394067 | 9.899318 | 10.77082 | 0.318246 | 0.29194 | 0.214496 | 1.99E-09 | 2.20E-06 |
| piR-mmu-23061108 | 5.15005 | 8.432179 | 9.612841 | 0.554877 | 0.318266 | 0.739589 | 2.10E-09 | 2.31E-06 |
| piR-mmu-48906998 | 5.734571 | 8.846223 | 9.455816 | 0.676172 | 0.195041 | 0.368177 | 2.13E-09 | 2.35E-06 |
| piR-mmu-11461049 | 7.831382 | 3.227082 | 3.459768 | 0.164814 | 0.587455 | 0.270473 | 3.00E-09 | 3.32E-06 |
| piR-mmu-49659703 | 9.51909 | 4.712022 | 5.762557 | 0.379303 | 0.524145 | 0.255331 | 3.35E-09 | 3.70E-06 |
| piR-mmu-49952640 | 5.15005 | 9.073816 | 8.406652 | 0.554877 | 0.146685 | 0.056975 | 3.36E-09 | 3.71E-06 |
| piR-mmu-31182420 | 5.15005 | 8.855188 | 8.368443 | 0.554877 | 0.189043 | 0.274056 | 3.68E-09 | 4.06E-06 |
| piR-mmu-49080070 | 5.298168 | 8.575196 | 8.325364 | 0.495614 | 0.242747 | 0.39256 | 4.34E-09 | 4.79E-06 |
| piR-mmu-10991144 | 8.439477 | 3.910995 | 3.865678 | 0.188807 | 0.635618 | 0.437285 | 5.50E-09 | 6.08E-06 |
| piR-mmu-49773186 | 6.751307 | 11.08459 | 7.312575 | 0.479732 | 0.329914 | 0.135458 | 5.64E-09 | 6.23E-06 |
| piR-mmu-29895302 | 5.899725 | 9.894493 | 9.918986 | 0.786352 | 0.223651 | 0.893297 | 5.76E-09 | 6.36E-06 |
| piR-mmu-51487042 | 6.014965 | 9.997085 | 9.125916 | 0.560519 | 0.592697 | 0.612345 | 6.19E-09 | 6.84E-06 |
| piR-mmu-32883726 | 5.923341 | 9.27594 | 9.006999 | 0.178182 | 0.679798 | 0.210836 | 6.95E-09 | 7.68E-06 |
| piR-mmu-49933547 | 5.75779 | 5.389256 | 9.089344 | 0.462186 | 0.049603 | 0.783662 | 6.97E-09 | 7.69E-06 |
| piR-mmu-2672873 | 5.15005 | 8.25819 | 9.136136 | 0.554877 | 0.159099 | 0.633959 | 7.50E-09 | 8.28E-06 |
| piR-mmu-29637882 | 5.15005 | 8.019067 | 9.186451 | 0.554877 | 0.212343 | 0.451819 | 7.55E-09 | 8.34E-06 |
| piR-mmu-20597713 | 7.203771 | 12.23855 | 11.87161 | 1.220746 | 0.389528 | 0.632727 | 8.22E-09 | 9.07E-06 |
| piR-mmu-51274259 | 6.017372 | 9.123959 | 8.67975 | 0.256763 | 0.218963 | 0.441455 | 9.23E-09 | 1.02E-05 |
| piR-mmu-48866107 | 5.595204 | 9.032033 | 5.723512 | 0.419958 | 0.446255 | 0.597236 | 1.05E-08 | 1.16E-05 |
| piR-mmu-49241740 | 6.279664 | 9.685847 | 10.69884 | 0.222912 | 0.703932 | 0.900408 | 1.09E-08 | 1.21E-05 |
| piR-mmu-49382286 | 5.158933 | 8.65092 | 8.249933 | 0.570095 | 0.267719 | 0.159262 | 1.34E-08 | 1.48E-05 |
| piR-mmu-51643821 | 7.519207 | 12.6145 | 9.596769 | 1.102484 | 0.543142 | 0.290728 | 1.39E-08 | 1.54E-05 |
| piR-mmu-22671960 | 5.97871 | 9.647747 | 8.370724 | 0.160223 | 0.613471 | 0.201582 | 1.41E-08 | 1.55E-05 |
| piR-mmu-49588383 | 7.083072 | 13.01262 | 10.72906 | 0.787661 | 0.766097 | 1.287702 | 1.47E-08 | 1.63E-05 |
| piR-mmu-33480893 | 5.462742 | 6.221246 | 9.113601 | 0.315912 | 0.11925 | 0.754174 | 1.48E-08 | 1.63E-05 |
| piR-mmu-18556972 | 5.27125 | 8.584016 | 8.476492 | 0.517445 | 0.1457 | 0.329352 | 1.50E-08 | 1.65E-05 |
| piR-mmu-49933562 | 7.526843 | 12.04363 | 10.46176 | 0.638879 | 0.481849 | 0.382207 | 1.59E-08 | 1.75E-05 |

| | | | | | | | | |
|---|---|---|---|---|---|---|---|---|
| piR-mmu-49569681 | 6.25268 | 7.042786 | 9.935162 | 0.427913 | 0.422728 | 0.455765 | 1.72E-08 | 1.90E-05 |
| piR-mmu-10912700 | 9.019803 | 5.378028 | 4.955719 | 0.150037 | 0.350307 | 0.811744 | 1.85E-08 | 2.04E-05 |
| piR-mmu-12389572 | 8.370798 | 12.76121 | 11.56309 | 0.427422 | 0.807022 | 0.400508 | 1.89E-08 | 2.09E-05 |
| piR-mmu-33182941 | 5.47189 | 9.420339 | 5.377668 | 0.456577 | 0.266522 | 1.121171 | 1.89E-08 | 2.09E-05 |
| piR-mmu-49087018 | 6.021527 | 7.814657 | 9.737039 | 0.407342 | 0.257139 | 0.661694 | 1.99E-08 | 2.19E-05 |
| piR-mmu-48841475 | 6.069887 | 10.16306 | 7.650951 | 0.71545 | 0.63043 | 0.556374 | 2.47E-08 | 2.72E-05 |
| piR-mmu-49837945 | 5.15005 | 8.870562 | 7.90699 | 0.554877 | 0.26627 | 0.12543 | 2.52E-08 | 2.78E-05 |
| piR-mmu-23770174 | 6.085307 | 5.644995 | 9.801009 | 0.435243 | 1.135329 | 0.391661 | 2.54E-08 | 2.81E-05 |
| piR-mmu-22987998 | 5.250023 | 8.654647 | 7.73253 | 0.507556 | 0.351792 | 0.304585 | 3.00E-08 | 3.32E-05 |
| piR-mmu-49382307 | 6.011552 | 7.781772 | 10.32848 | 0.450553 | 0.235632 | 1.226581 | 3.20E-08 | 3.53E-05 |
| piR-mmu-49033791 | 5.590985 | 8.324095 | 8.921579 | 0.380193 | 0.170834 | 0.800676 | 3.55E-08 | 3.92E-05 |
| piR-mmu-49673022 | 5.213775 | 8.686233 | 7.65827 | 0.542266 | 0.260251 | 0.312388 | 3.55E-08 | 3.92E-05 |
| piR-mmu-49326551 | 5.232933 | 8.208439 | 8.098537 | 0.503115 | 0.238952 | 0.246988 | 3.71E-08 | 4.10E-05 |
| piR-mmu-10834668 | 5.15005 | 7.419065 | 9.869112 | 0.554877 | 0.318432 | 1.305473 | 4.23E-08 | 4.67E-05 |
| piR-mmu-11031895 | 7.58526 | 3.818405 | 3.666734 | 0.223864 | 0.202072 | 0.497063 | 4.24E-08 | 4.68E-05 |
| piR-mmu-49012494 | 6.35505 | 10.39922 | 6.521731 | 0.689823 | 0.532296 | 0.45326 | 4.35E-08 | 4.81E-05 |
| piR-mmu-49654384 | 6.041147 | 9.400683 | 7.074314 | 0.782673 | 0.381656 | 0.27158 | 4.67E-08 | 5.15E-05 |
| piR-mmu-49581248 | 8.172059 | 13.51836 | 11.35049 | 0.815937 | 0.973087 | 0.999903 | 4.91E-08 | 5.42E-05 |
| piR-mmu-12465402 | 6.126048 | 9.545263 | 8.698818 | 0.369055 | 0.412143 | 0.726986 | 5.06E-08 | 5.59E-05 |
| piR-mmu-48843118 | 5.173474 | 8.453387 | 8.237587 | 0.530532 | 0.214422 | 0.657828 | 5.37E-08 | 5.93E-05 |
| piR-mmu-49232656 | 7.503123 | 11.55595 | 10.26856 | 0.518236 | 0.457277 | 0.416419 | 5.43E-08 | 6.00E-05 |
| piR-mmu-31167747 | 5.164249 | 9.086734 | 7.898283 | 0.54637 | 0.802484 | 0.259897 | 5.45E-08 | 6.02E-05 |
| piR-mmu-51113620 | 5.728505 | 8.035786 | 8.771263 | 0.450867 | 0.087553 | 0.374874 | 5.71E-08 | 6.30E-05 |
| piR-mmu-49019848 | 9.051946 | 13.57422 | 11.53909 | 1.138489 | 0.350275 | 0.413354 | 6.09E-08 | 6.73E-05 |
| piR-mmu-18257020 | 6.742615 | 10.88126 | 8.703871 | 0.854295 | 0.399511 | 0.229938 | 6.28E-08 | 6.93E-05 |
| piR-mmu-10991141 | 8.657402 | 5.195215 | 4.93923 | 0.158311 | 0.41419 | 0.738379 | 6.32E-08 | 6.98E-05 |
| piR-mmu-10940440 | 8.908801 | 5.314249 | 4.964897 | 0.204908 | 0.677881 | 0.648399 | 6.63E-08 | 7.32E-05 |
| piR-mmu-10934588 | 8.501352 | 4.201323 | 4.41681 | 0.155096 | 0.697259 | 0.613556 | 6.68E-08 | 7.37E-05 |
| piR-mmu-49285797 | 5.250764 | 8.270168 | 7.697008 | 0.471369 | 0.191989 | 0.083471 | 6.72E-08 | 7.42E-05 |
| piR-mmu-48753165 | 8.530879 | 12.21483 | 12.57544 | 0.588704 | 0.424104 | 0.681559 | 7.34E-08 | 8.10E-05 |
| piR-mmu-23829230 | 5.539701 | 9.12877 | 7.516856 | 0.456486 | 0.229009 | 0.967028 | 7.96E-08 | 8.79E-05 |
| piR-mmu-8855247 | 5.742301 | 6.533495 | 10.2249 | 0.619377 | 0.344106 | 1.514327 | 8.01E-08 | 8.85E-05 |
| piR-mmu-49698133 | 5.794405 | 4.41104 | 8.942213 | 0.58773 | 0.705548 | 1.089062 | 8.59E-08 | 9.49E-05 |
| piR-mmu-10940441 | 8.500941 | 5.03694 | 4.332035 | 0.151103 | 0.590197 | 0.647477 | 8.62E-08 | 9.51E-05 |
| piR-mmu-31375764 | 5.15005 | 8.829099 | 7.928576 | 0.554877 | 0.441288 | 0.511066 | 8.68E-08 | 9.59E-05 |
| piR-mmu-41640190 | 6.262204 | 9.78718 | 8.194538 | 0.62874 | 0.34805 | 0.351869 | 8.96E-08 | 9.90E-05 |
| piR-mmu-7719044 | 6.340522 | 10.39298 | 7.838189 | 0.213712 | 0.640536 | 0.672611 | 9.59E-08 | 0.000106 |
| piR-mmu-51029016 | 7.769701 | 11.91035 | 11.04514 | 0.471187 | 0.845009 | 0.481716 | 1.10E-07 | 0.000121 |
| piR-mmu-31551436 | 5.918102 | 9.019492 | 7.123002 | 0.222429 | 0.369406 | 0.261168 | 1.12E-07 | 0.000124 |

| | | | | | | | | |
|---|---|---|---|---|---|---|---|---|
| piR-mmu-22674430 | 5.259647 | 7.847954 | 8.522718 | 0.486441 | 0.342926 | 0.336344 | 1.13E-07 | 0.000125 |
| piR-mmu-50675109 | 9.332318 | 12.74613 | 12.91409 | 0.787817 | 0.077376 | 0.582716 | 1.15E-07 | 0.000127 |
| piR-mmu-5610469 | 5.990627 | 6.766829 | 9.306074 | 0.337435 | 0.585676 | 0.543532 | 1.28E-07 | 0.000142 |
| piR-mmu-15983767 | 6.391659 | 10.87489 | 8.535181 | 1.037321 | 0.480604 | 0.423725 | 1.35E-07 | 0.000149 |
| piR-mmu-11461014 | 8.787251 | 4.760203 | 4.634775 | 0.086673 | 0.857346 | 0.727927 | 1.38E-07 | 0.000152 |
| piR-mmu-33199875 | 14.47334 | 11.81288 | 10.58285 | 0.30886 | 0.565109 | 1.021616 | 1.44E-07 | 0.000159 |
| piR-mmu-49287809 | 9.095436 | 5.619299 | 5.175905 | 0.376088 | 0.666893 | 0.710208 | 1.54E-07 | 0.00017 |
| piR-mmu-1395616 | 6.287623 | 9.477844 | 7.866021 | 0.256231 | 0.094607 | 0.439529 | 1.56E-07 | 0.000172 |
| piR-mmu-10960276 | 8.086195 | 4.878327 | 4.018662 | 0.213635 | 0.489612 | 0.83721 | 1.61E-07 | 0.000178 |
| piR-mmu-29446216 | 5.293697 | 7.925616 | 7.732174 | 0.488806 | 0.122804 | 0.242607 | 1.61E-07 | 0.000178 |
| piR-mmu-49597408 | 5.232933 | 8.317475 | 8.111378 | 0.503115 | 0.410303 | 0.632287 | 1.63E-07 | 0.00018 |
| piR-mmu-49544423 | 6.264344 | 8.562567 | 10.43317 | 1.1196 | 0.262805 | 0.899261 | 1.67E-07 | 0.000185 |
| piR-mmu-8117275 | 6.934675 | 11.37606 | 9.359973 | 1.199739 | 0.363795 | 0.169896 | 1.77E-07 | 0.000195 |
| piR-mmu-49039221 | 5.15005 | 8.274018 | 7.358781 | 0.554877 | 0.174456 | 0.153788 | 1.83E-07 | 0.000202 |
| piR-mmu-7474227 | 9.38674 | 6.189675 | 5.665049 | 0.093529 | 0.545149 | 0.922661 | 1.88E-07 | 0.000208 |
| piR-mmu-4213313 | 6.697146 | 9.91544 | 10.91775 | 1.120732 | 0.598208 | 0.368005 | 1.95E-07 | 0.000215 |
| piR-mmu-50063001 | 7.325331 | 10.73829 | 11.18788 | 0.878548 | 0.075752 | 0.451351 | 2.04E-07 | 0.000226 |
| piR-mmu-16168384 | 6.576752 | 8.043489 | 10.8472 | 1.041081 | 0.303017 | 0.697361 | 2.08E-07 | 0.00023 |
| piR-mmu-50769050 | 9.567962 | 5.830838 | 6.644783 | 0.480003 | 0.271591 | 0.449119 | 2.15E-07 | 0.000237 |
| piR-mmu-50433158 | 8.670875 | 4.998029 | 5.6868 | 0.553755 | 0.296602 | 0.426545 | 2.21E-07 | 0.000244 |
| piR-mmu-50642085 | 5.907762 | 8.961841 | 8.560374 | 0.475786 | 0.139757 | 0.770191 | 2.29E-07 | 0.000253 |
| piR-mmu-10916134 | 8.326815 | 5.438947 | 4.929679 | 0.194624 | 0.363756 | 0.285463 | 2.36E-07 | 0.000261 |
| piR-mmu-1996661 | 8.821273 | 11.75874 | 13.76222 | 1.240297 | 0.463161 | 0.947619 | 2.39E-07 | 0.000264 |
| piR-mmu-4589701 | 6.142416 | 9.007192 | 8.478244 | 0.081337 | 0.302226 | 0.272007 | 2.40E-07 | 0.000265 |
| piR-mmu-11031886 | 8.487193 | 5.016835 | 4.617445 | 0.055488 | 0.570872 | 0.752002 | 2.51E-07 | 0.000277 |
| piR-mmu-3447241 | 5.956158 | 6.874357 | 9.257937 | 0.262845 | 0.771845 | 0.47863 | 2.52E-07 | 0.000278 |
| piR-mmu-10911100 | 9.254792 | 5.975397 | 5.590992 | 0.126515 | 0.418464 | 0.819511 | 2.57E-07 | 0.000283 |
| piR-mmu-7425154 | 8.026957 | 4.936781 | 4.244071 | 0.276842 | 0.563058 | 0.635839 | 2.62E-07 | 0.000289 |
| piR-mmu-49205030 | 5.37697 | 8.122776 | 7.049101 | 0.415804 | 0.125672 | 0.09431 | 2.65E-07 | 0.000293 |
| piR-mmu-49224045 | 7.597267 | 11.44538 | 11.58604 | 1.389622 | 0.191575 | 0.613602 | 2.68E-07 | 0.000296 |
| piR-mmu-49115216 | 10.26824 | 13.03387 | 13.79316 | 0.272606 | 0.494095 | 0.482476 | 2.86E-07 | 0.000316 |
| piR-mmu-49362409 | 5.797761 | 8.438295 | 8.18114 | 0.518101 | 0.165572 | 0.480947 | 3.04E-07 | 0.000336 |
| piR-mmu-49092060 | 5.608102 | 8.20684 | 7.973137 | 0.2751 | 0.215895 | 0.104471 | 3.06E-07 | 0.000338 |
| piR-mmu-16474472 | 5.627487 | 8.870892 | 6.497755 | 0.496171 | 0.604497 | 0.302872 | 3.14E-07 | 0.000347 |
| piR-mmu-51015918 | 8.792336 | 5.522712 | 5.736947 | 0.272205 | 0.103976 | 0.396465 | 3.18E-07 | 0.000351 |
| piR-mmu-49823282 | 8.629291 | 5.139206 | 5.755066 | 0.429578 | 0.405527 | 0.345846 | 3.35E-07 | 0.00037 |
| piR-mmu-49973886 | 9.803798 | 5.837109 | 6.395967 | 0.146429 | 0.288981 | 0.650906 | 3.42E-07 | 0.000378 |
| piR-mmu-50920012 | 5.746641 | 8.786812 | 6.818866 | 0.361585 | 0.238098 | 0.558011 | 3.55E-07 | 0.000392 |
| piR-mmu-51625409 | 9.090178 | 9.919294 | 13.55212 | 1.436288 | 0.426177 | 0.750406 | 3.77E-07 | 0.000416 |

| | | | | | | | | |
|---|---|---|---|---|---|---|---|---|
| piR-mmu-20743584 | 8.599392 | 6.123449 | 4.645802 | 0.300336 | 0.699845 | 0.411834 | 3.81E-07 | 0.000421 |
| piR-mmu-24780774 | 5.502449 | 7.942628 | 7.778368 | 0.398977 | 0.221477 | 0.343328 | 3.84E-07 | 0.000424 |
| piR-mmu-10932261 | 9.169739 | 5.981045 | 5.367342 | 0.144462 | 0.612888 | 0.829605 | 3.95E-07 | 0.000437 |
| piR-mmu-3078829 | 5.187062 | 8.87548 | 6.927667 | 0.553964 | 0.596496 | 0.809741 | 3.96E-07 | 0.000437 |
| piR-mmu-10906591 | 16.71583 | 13.97554 | 12.97739 | 0.028771 | 0.620408 | 1.079699 | 4.03E-07 | 0.000445 |
| piR-mmu-10911823 | 9.170175 | 6.111828 | 5.488273 | 0.173396 | 0.481199 | 0.755892 | 4.04E-07 | 0.000446 |
| piR-mmu-10978311 | 7.840821 | 5.309242 | 4.259595 | 0.221247 | 0.381587 | 0.755246 | 4.18E-07 | 0.000462 |
| piR-mmu-49427307 | 9.419166 | 6.980699 | 6.069427 | 0.510991 | 0.276694 | 0.146718 | 4.39E-07 | 0.000485 |
| piR-mmu-25580810 | 8.447645 | 12.08875 | 11.54713 | 0.661896 | 0.687285 | 0.581423 | 4.48E-07 | 0.000494 |
| piR-mmu-49909396 | 5.79033 | 8.990596 | 6.33238 | 0.429559 | 0.279289 | 0.756561 | 4.53E-07 | 0.000501 |
| piR-mmu-49118066 | 7.600456 | 5.07719 | 4.084155 | 0.381216 | 0.343345 | 0.342678 | 4.58E-07 | 0.000506 |
| piR-mmu-12112309 | 8.888458 | 12.72464 | 12.70091 | 1.364692 | 0.080906 | 0.249148 | 4.75E-07 | 0.000525 |
| piR-mmu-4704882 | 6.429275 | 10.23249 | 8.347158 | 0.907712 | 0.298605 | 0.179584 | 4.94E-07 | 0.000545 |
| piR-mmu-29978745 | 5.15005 | 8.02254 | 7.577541 | 0.554877 | 0.202322 | 0.246633 | 5.01E-07 | 0.000553 |
| piR-mmu-1275617 | 6.655362 | 7.246865 | 9.788821 | 0.044382 | 0.321163 | 0.361733 | 5.03E-07 | 0.000555 |
| piR-mmu-85958 | 16.70036 | 14.34846 | 13.38272 | 0.352028 | 0.219821 | 0.82999 | 5.89E-07 | 0.000651 |
| piR-mmu-51389087 | 5.15005 | 8.269196 | 7.216026 | 0.554877 | 0.120398 | 0.082929 | 6.08E-07 | 0.000671 |
| piR-mmu-51430009 | 6.342917 | 6.298657 | 9.304158 | 0.651361 | 0.250279 | 0.352705 | 6.16E-07 | 0.00068 |
| piR-mmu-48942900 | 7.577654 | 10.32499 | 10.80663 | 0.544103 | 0.160223 | 0.24646 | 6.47E-07 | 0.000714 |
| piR-mmu-10953882 | 8.904071 | 5.539356 | 5.414267 | 0.148529 | 0.55955 | 0.652457 | 6.62E-07 | 0.000731 |
| piR-mmu-11065039 | 7.816388 | 11.84815 | 10.988 | 0.683414 | 0.639694 | 1.14166 | 7.02E-07 | 0.000775 |
| piR-mmu-2001062 | 5.479745 | 3.525885 | 7.148306 | 0.353337 | 0.771477 | 0.454687 | 7.25E-07 | 0.0008 |
| piR-mmu-10910310 | 10.22279 | 7.754421 | 6.251734 | 0.125766 | 0.668621 | 0.957331 | 7.29E-07 | 0.000805 |
| piR-mmu-7719646 | 8.127894 | 5.060983 | 4.540183 | 0.293166 | 0.253327 | 0.96444 | 7.67E-07 | 0.000847 |
| piR-mmu-48782409 | 5.178179 | 7.64989 | 7.732366 | 0.538986 | 0.230487 | 0.252759 | 8.64E-07 | 0.000954 |
| piR-mmu-18494776 | 6.005717 | 8.031514 | 8.904245 | 0.242086 | 0.551226 | 0.530218 | 8.91E-07 | 0.000984 |
| piR-mmu-5093664 | 8.04862 | 11.41533 | 10.80107 | 0.858568 | 0.39795 | 0.391669 | 9.11E-07 | 0.001006 |
| piR-mmu-49615547 | 6.758346 | 10.20742 | 11.03159 | 1.390446 | 0.066464 | 0.774353 | 9.24E-07 | 0.00102 |
| piR-mmu-49613252 | 5.272219 | 7.632196 | 7.989142 | 0.447475 | 0.295456 | 0.046439 | 9.87E-07 | 0.00109 |
| piR-mmu-50378326 | 8.444847 | 11.40833 | 11.59005 | 0.841725 | 0.274716 | 0.440534 | 1.02E-06 | 0.001131 |
| piR-mmu-49714631 | 7.39974 | 6.148673 | 3.930353 | 0.302992 | 0.460166 | 0.492526 | 1.04E-06 | 0.001151 |
| piR-mmu-10879220 | 5.298027 | 8.38539 | 6.423259 | 0.491951 | 0.497285 | 0.351575 | 1.08E-06 | 0.001189 |
| piR-mmu-29271766 | 11.77436 | 9.234084 | 7.925069 | 0.31301 | 0.542792 | 0.885497 | 1.09E-06 | 0.001208 |
| piR-mmu-51145640 | 6.273702 | 7.723474 | 9.292517 | 0.371243 | 0.713459 | 0.269636 | 1.11E-06 | 0.001227 |
| piR-mmu-6049578 | 7.437362 | 11.42809 | 9.83497 | 1.318983 | 0.280897 | 0.228958 | 1.12E-06 | 0.001241 |
| piR-mmu-50884454 | 6.610728 | 10.02755 | 8.434765 | 0.393945 | 0.720409 | 0.392883 | 1.13E-06 | 0.001243 |
| piR-mmu-10909435 | 9.257176 | 6.628314 | 5.781335 | 0.09225 | 0.375187 | 0.980443 | 1.16E-06 | 0.001277 |
| piR-mmu-49248879 | 6.234551 | 9.134487 | 8.585095 | 0.433591 | 0.51576 | 0.683921 | 1.16E-06 | 0.001281 |
| piR-mmu-10882589 | 6.799763 | 10.20454 | 8.931421 | 0.502148 | 0.517195 | 0.270898 | 1.23E-06 | 0.001359 |

| | | | | | | | | |
|---|---|---|---|---|---|---|---|---|
| piR-mmu-48899520 | 13.07643 | 9.682496 | 9.603453 | 0.559318 | 0.378683 | 1.205807 | 1.23E-06 | 0.001362 |
| piR-mmu-48911844 | 10.1134 | 7.150765 | 7.58682 | 0.348919 | 0.408214 | 0.557144 | 1.40E-06 | 0.001545 |
| piR-mmu-10911632 | 9.008779 | 5.806274 | 5.237652 | 0.063744 | 0.628441 | 1.069517 | 1.41E-06 | 0.00156 |
| piR-mmu-49550745 | 5.19172 | 6.299442 | 8.955102 | 0.532758 | 0.247511 | 1.330675 | 1.48E-06 | 0.001637 |
| piR-mmu-2004883 | 7.476168 | 9.049655 | 11.41162 | 0.884439 | 0.341857 | 0.972046 | 1.49E-06 | 0.001642 |
| piR-mmu-49308008 | 8.670316 | 6.62997 | 5.18286 | 0.416201 | 0.47603 | 0.156802 | 1.49E-06 | 0.001644 |
| piR-mmu-48741163 | 5.376003 | 7.982424 | 6.630218 | 0.453055 | 0.257171 | 0.295747 | 1.54E-06 | 0.001699 |
| piR-mmu-10940438 | 8.210238 | 4.549404 | 4.241595 | 0.279598 | 0.804566 | 1.238049 | 1.57E-06 | 0.001736 |
| piR-mmu-50003926 | 5.15005 | 7.659926 | 7.614041 | 0.554877 | 0.140063 | 0.203429 | 1.59E-06 | 0.001755 |
| piR-mmu-72373 | 9.290186 | 7.745465 | 5.796298 | 1.019776 | 0.388048 | 0.278786 | 1.61E-06 | 0.00178 |
| piR-mmu-5808923 | 6.404205 | 9.806518 | 7.718845 | 0.130633 | 0.790935 | 0.431462 | 1.62E-06 | 0.001791 |
| piR-mmu-50207618 | 20.52026 | 17.72975 | 16.99092 | 0.151505 | 0.69064 | 1.15932 | 1.63E-06 | 0.001801 |
| piR-mmu-49508490 | 5.394587 | 7.796824 | 7.921617 | 0.441945 | 0.22065 | 0.563299 | 1.69E-06 | 0.001869 |
| piR-mmu-7073709 | 8.8502 | 12.50497 | 11.9505 | 1.368842 | 0.216072 | 0.106038 | 1.79E-06 | 0.001979 |
| piR-mmu-2692796 | 8.133247 | 6.732823 | 4.704361 | 0.256382 | 0.493568 | 0.578319 | 1.82E-06 | 0.002015 |
| piR-mmu-49377801 | 7.799159 | 11.11428 | 9.840577 | 0.860255 | 0.233827 | 0.434855 | 1.84E-06 | 0.002029 |
| piR-mmu-29261732 | 9.438859 | 10.59309 | 7.51579 | 0.686445 | 0.415146 | 0.283848 | 1.85E-06 | 0.002038 |
| piR-mmu-49361860 | 8.565421 | 6.769727 | 4.857444 | 0.597649 | 0.565582 | 0.527361 | 2.00E-06 | 0.002204 |
| piR-mmu-49777149 | 6.941687 | 8.065948 | 10.55196 | 0.863135 | 0.364425 | 0.521907 | 2.02E-06 | 0.00223 |
| piR-mmu-10856482 | 5.20529 | 6.813013 | 9.178682 | 0.550931 | 0.318184 | 1.518146 | 2.04E-06 | 0.002256 |
| piR-mmu-240378 | 15.68287 | 14.75963 | 12.56915 | 0.372211 | 0.506789 | 0.824103 | 2.12E-06 | 0.002339 |
| piR-mmu-32331990 | 5.182357 | 6.640634 | 8.735867 | 0.545857 | 0.42655 | 1.11578 | 2.16E-06 | 0.002388 |
| piR-mmu-1994694 | 5.862405 | 8.945896 | 5.655384 | 0.211242 | 1.4606 | 0.242902 | 2.19E-06 | 0.002416 |
| piR-mmu-2041313 | 7.366998 | 11.1951 | 9.620152 | 0.779925 | 1.127385 | 0.384151 | 2.21E-06 | 0.002442 |
| piR-mmu-13828175 | 7.259749 | 3.954346 | 4.27727 | 0.589611 | 0.28426 | 0.351462 | 2.23E-06 | 0.002467 |
| piR-mmu-49391324 | 10.52253 | 8.296355 | 7.303789 | 0.383225 | 0.246239 | 0.682009 | 2.25E-06 | 0.002487 |
| piR-mmu-10907457 | 10.82699 | 8.226629 | 7.232688 | 0.307625 | 0.498926 | 0.910951 | 2.32E-06 | 0.002566 |
| piR-mmu-48799366 | 7.674723 | 4.758569 | 4.91387 | 0.475571 | 0.150108 | 0.229654 | 2.49E-06 | 0.002754 |
| piR-mmu-2760499 | 14.35281 | 13.0764 | 11.66238 | 0.324212 | 0.174461 | 0.418969 | 2.57E-06 | 0.002836 |
| piR-mmu-49599562 | 10.56144 | 7.761591 | 7.678816 | 0.116039 | 0.746119 | 0.562395 | 2.60E-06 | 0.002867 |
| piR-mmu-26860261 | 9.200038 | 6.483039 | 6.10972 | 0.4233 | 0.656817 | 0.584648 | 2.61E-06 | 0.002881 |
| piR-mmu-51194478 | 7.913513 | 11.07214 | 9.942886 | 0.674663 | 0.70234 | 0.24566 | 2.62E-06 | 0.002889 |
| piR-mmu-11663635 | 7.827281 | 5.180671 | 4.857439 | 0.35159 | 0.612585 | 0.636837 | 2.73E-06 | 0.003017 |
| piR-mmu-10906686 | 8.211093 | 4.646317 | 4.77175 | 0.660359 | 0.376073 | 1.084405 | 2.83E-06 | 0.003124 |
| piR-mmu-26426124 | 6.134832 | 8.920991 | 7.793253 | 0.69451 | 0.353726 | 0.530925 | 3.09E-06 | 0.003416 |
| piR-mmu-49623631 | 7.380719 | 10.59183 | 9.567407 | 0.846506 | 0.395288 | 0.105672 | 3.15E-06 | 0.003481 |
| piR-mmu-48805945 | 6.993775 | 10.46061 | 9.058865 | 0.594192 | 0.53616 | 0.133652 | 3.20E-06 | 0.003534 |
| piR-mmu-50062170 | 5.822566 | 8.266445 | 8.069891 | 0.583283 | 0.245154 | 0.667773 | 3.28E-06 | 0.003626 |
| piR-mmu-51294737 | 7.059209 | 3.659042 | 3.412649 | 0.539499 | 0.886144 | 0.381325 | 3.30E-06 | 0.003648 |

| | | | | | | | | |
|---|---|---|---|---|---|---|---|---|
| piR-mmu-2618472 | 12.52104 | 11.14293 | 9.520583 | 0.333304 | 0.141049 | 0.319337 | 3.31E-06 | 0.003653 |
| piR-mmu-27200780 | 8.000454 | 6.472638 | 4.912414 | 0.274638 | 0.068637 | 0.816221 | 3.50E-06 | 0.003863 |
| piR-mmu-48882277 | 7.945725 | 6.474342 | 4.8149 | 0.214656 | 0.464959 | 0.64771 | 3.51E-06 | 0.003878 |
| piR-mmu-49493928 | 5.877385 | 7.842784 | 9.491075 | 0.605953 | 0.912022 | 1.267387 | 3.54E-06 | 0.003914 |
| piR-mmu-10718433 | 7.017807 | 6.676283 | 10.19579 | 0.732362 | 1.043989 | 0.32353 | 3.57E-06 | 0.003938 |
| piR-mmu-15658525 | 6.420198 | 7.238486 | 9.752767 | 0.905034 | 0.16995 | 0.59321 | 3.80E-06 | 0.004195 |
| piR-mmu-48949488 | 5.991379 | 8.583455 | 6.424379 | 0.735038 | 0.133786 | 0.329852 | 4.02E-06 | 0.004443 |
| piR-mmu-7474220 | 10.82525 | 7.659336 | 8.816542 | 0.408047 | 0.32561 | 0.559642 | 4.05E-06 | 0.004477 |
| piR-mmu-11663672 | 7.743442 | 4.199059 | 4.796372 | 0.551066 | 0.910293 | 0.536521 | 4.22E-06 | 0.004664 |
| piR-mmu-29521986 | 6.71111 | 10.33882 | 7.585598 | 0.261209 | 1.313086 | 0.705133 | 4.58E-06 | 0.005061 |
| piR-mmu-24711038 | 5.266051 | 7.715008 | 7.086142 | 0.448278 | 0.244631 | 0.469 | 4.59E-06 | 0.00507 |
| piR-mmu-48729792 | 7.945213 | 5.004343 | 5.052365 | 0.141262 | 0.60082 | 0.814533 | 4.78E-06 | 0.005274 |
| piR-mmu-7474228 | 9.980355 | 6.8163 | 7.708103 | 0.332775 | 0.262706 | 0.706878 | 5.06E-06 | 0.005584 |
| piR-mmu-49609791 | 8.498115 | 6.524281 | 4.978015 | 0.843415 | 0.420725 | 0.477631 | 5.09E-06 | 0.005615 |
| piR-mmu-10843785 | 8.01296 | 7.378577 | 10.42935 | 1.079667 | 0.239065 | 0.377541 | 5.23E-06 | 0.005769 |
| piR-mmu-49257406 | 8.24247 | 9.421308 | 11.96635 | 1.484544 | 0.22379 | 0.467532 | 5.33E-06 | 0.005888 |
| piR-mmu-12363372 | 9.285227 | 7.35348 | 5.771644 | 1.339932 | 0.279096 | 0.282008 | 5.70E-06 | 0.00629 |
| piR-mmu-30257664 | 9.445169 | 10.34701 | 7.43995 | 0.690172 | 0.379365 | 0.383495 | 5.92E-06 | 0.006536 |
| piR-mmu-2201360 | 7.119097 | 8.978778 | 11.18654 | 1.067111 | 0.17253 | 1.164653 | 5.96E-06 | 0.006584 |
| piR-mmu-8379514 | 6.622364 | 6.197301 | 8.847092 | 0.072181 | 0.477445 | 0.400561 | 5.99E-06 | 0.006614 |
| piR-mmu-10934587 | 8.693607 | 6.114771 | 5.525992 | 0.230376 | 0.435112 | 0.841673 | 6.34E-06 | 0.006998 |
| piR-mmu-9022216 | 7.113574 | 10.68702 | 8.999236 | 1.188609 | 0.333782 | 0.104875 | 6.52E-06 | 0.007194 |
| piR-mmu-48890693 | 9.864513 | 12.28318 | 12.93912 | 0.182877 | 0.429406 | 0.547153 | 6.64E-06 | 0.007326 |
| piR-mmu-49658450 | 5.899551 | 7.927312 | 8.507981 | 0.589915 | 0.217481 | 0.61792 | 6.88E-06 | 0.007599 |
| piR-mmu-48810876 | 7.989998 | 9.756279 | 11.44511 | 1.22001 | 0.487962 | 0.507502 | 6.89E-06 | 0.007606 |
| piR-mmu-49015749 | 12.86147 | 11.97195 | 9.747566 | 0.409896 | 0.445825 | 0.7636 | 6.94E-06 | 0.007666 |
| piR-mmu-50046028 | 8.449156 | 5.633704 | 5.319871 | 0.147332 | 0.320844 | 0.960873 | 7.03E-06 | 0.007756 |
| piR-mmu-5611284 | 6.330003 | 7.714953 | 9.622114 | 0.938557 | 0.299616 | 0.772625 | 7.21E-06 | 0.00796 |
| piR-mmu-49413063 | 8.061575 | 4.832312 | 5.242824 | 0.204806 | 0.892659 | 0.624654 | 7.39E-06 | 0.008155 |
| piR-mmu-8422651 | 6.591113 | 9.644249 | 8.46939 | 0.595955 | 0.36376 | 0.527188 | 7.52E-06 | 0.008297 |
| piR-mmu-49897460 | 15.91163 | 13.21208 | 12.71857 | 0.610685 | 0.49207 | 1.242274 | 7.56E-06 | 0.008342 |
| piR-mmu-49735293 | 9.511419 | 6.507697 | 7.272849 | 0.389742 | 0.076029 | 0.718808 | 7.66E-06 | 0.008462 |
| piR-mmu-34200 | 11.21122 | 9.232899 | 8.566129 | 0.346755 | 0.274261 | 0.283831 | 8.10E-06 | 0.008947 |
| piR-mmu-49804704 | 5.15005 | 6.171665 | 8.733763 | 0.554877 | 0.63966 | 1.482224 | 8.72E-06 | 0.009626 |
| piR-mmu-49822140 | 10.43499 | 8.012334 | 7.906484 | 0.468738 | 0.186906 | 0.463742 | 8.76E-06 | 0.009673 |
| piR-mmu-11066710 | 10.73449 | 8.183064 | 8.338899 | 0.183498 | 0.377009 | 0.650985 | 8.83E-06 | 0.009753 |
| piR-mmu-49224859 | 6.22965 | 8.555219 | 6.94873 | 0.300275 | 0.449468 | 0.437928 | 9.18E-06 | 0.010131 |
| piR-mmu-7489022 | 7.587968 | 4.740949 | 4.353133 | 0.325471 | 0.97168 | 0.650665 | 9.21E-06 | 0.010164 |
| piR-mmu-49838805 | 13.06354 | 10.31838 | 9.814057 | 0.546025 | 0.426125 | 1.178442 | 9.44E-06 | 0.010418 |

| | | | | | | | | |
|---|---|---|---|---|---|---|---|---|
| piR-mmu-11220195 | 13.63104 | 11.04577 | 11.02138 | 0.220207 | 0.510841 | 0.62488 | 9.63E-06 | 0.010632 |
| piR-mmu-49054943 | 8.636926 | 6.542112 | 5.976527 | 0.40793 | 0.035059 | 0.307999 | 9.77E-06 | 0.01079 |
| piR-mmu-49153404 | 5.340213 | 6.943701 | 8.434471 | 0.476839 | 0.64017 | 0.946158 | 1.01E-05 | 0.011103 |
| piR-mmu-49896624 | 7.880696 | 5.779684 | 4.970201 | 0.377477 | 0.588529 | 0.439668 | 1.02E-05 | 0.011262 |
| piR-mmu-49915338 | 8.739174 | 6.132398 | 5.484683 | 0.100555 | 0.568912 | 0.976529 | 1.03E-05 | 0.011398 |
| piR-mmu-72274 | 9.807956 | 6.291221 | 7.286231 | 0.401482 | 1.04273 | 0.465882 | 1.05E-05 | 0.01157 |
| piR-mmu-49565256 | 6.871939 | 9.869836 | 9.378294 | 0.758358 | 0.403073 | 0.720992 | 1.06E-05 | 0.011715 |
| piR-mmu-50516164 | 10.7438 | 9.377604 | 7.891294 | 0.342858 | 0.120759 | 0.390206 | 1.07E-05 | 0.01178 |
| piR-mmu-10991138 | 7.6793 | 5.056276 | 4.594643 | 0.309202 | 0.544167 | 0.917362 | 1.08E-05 | 0.011881 |
| piR-mmu-2439726 | 6.448435 | 9.781072 | 8.424182 | 0.583461 | 0.923901 | 0.496371 | 1.11E-05 | 0.012252 |
| piR-mmu-50304273 | 12.11005 | 13.24378 | 10.21945 | 0.755315 | 0.399992 | 0.304094 | 1.15E-05 | 0.012716 |
| piR-mmu-16071780 | 7.367531 | 8.400201 | 10.07397 | 0.649795 | 0.148865 | 0.147294 | 1.15E-05 | 0.012726 |
| piR-mmu-29894166 | 6.460966 | 8.571532 | 9.180376 | 0.26629 | 0.366434 | 0.638582 | 1.16E-05 | 0.012793 |
| piR-mmu-49094654 | 6.63703 | 6.57484 | 9.13026 | 0.659059 | 0.239644 | 0.562664 | 1.20E-05 | 0.01323 |
| piR-mmu-48897293 | 11.2542 | 9.087936 | 8.647718 | 0.364596 | 0.131111 | 0.591904 | 1.20E-05 | 0.013245 |
| piR-mmu-1283203 | 10.62302 | 10.60273 | 13.56431 | 1.099935 | 0.392028 | 0.323472 | 1.21E-05 | 0.013361 |
| piR-mmu-44699003 | 7.089614 | 6.234116 | 9.746314 | 1.171342 | 0.761771 | 0.402724 | 1.23E-05 | 0.013532 |
| piR-mmu-48824062 | 8.268448 | 11.48074 | 9.829226 | 0.982995 | 0.49076 | 0.422688 | 1.34E-05 | 0.014846 |
| piR-mmu-4945476 | 7.126532 | 6.318107 | 9.438444 | 0.995508 | 0.271743 | 0.216896 | 1.41E-05 | 0.015577 |
| piR-mmu-49155517 | 9.035335 | 7.016972 | 6.045797 | 0.178024 | 0.63782 | 0.815393 | 1.44E-05 | 0.015895 |
| piR-mmu-377765 | 6.590078 | 8.657053 | 8.929418 | 0.178718 | 0.246794 | 0.456491 | 1.46E-05 | 0.016077 |
| piR-mmu-11429767 | 6.293501 | 9.138722 | 8.422984 | 0.803984 | 0.499257 | 0.698957 | 1.52E-05 | 0.016778 |
| piR-mmu-16477705 | 5.351494 | 7.832507 | 7.213782 | 0.442874 | 0.396377 | 0.598123 | 1.56E-05 | 0.017175 |
| piR-mmu-25482018 | 9.196342 | 7.921032 | 6.509744 | 0.32034 | 0.179014 | 0.456418 | 1.56E-05 | 0.017208 |
| piR-mmu-49923410 | 6.425332 | 9.109425 | 7.087576 | 0.81532 | 0.153119 | 0.146426 | 1.56E-05 | 0.017264 |
| piR-mmu-31018127 | 7.881568 | 4.914338 | 5.093678 | 0.657226 | 0.962929 | 0.58418 | 1.58E-05 | 0.017477 |
| piR-mmu-50556010 | 9.352118 | 6.619248 | 7.096739 | 0.37347 | 0.474703 | 0.739299 | 1.62E-05 | 0.01792 |
| piR-mmu-2027261 | 6.366683 | 9.042537 | 7.844226 | 0.447454 | 0.359289 | 0.561471 | 1.67E-05 | 0.018421 |
| piR-mmu-3293489 | 7.330385 | 5.310355 | 4.350313 | 0.758375 | 0.249346 | 0.443755 | 1.68E-05 | 0.018533 |
| piR-mmu-49483892 | 17.03115 | 14.46958 | 15.16703 | 0.291639 | 0.470049 | 0.58884 | 1.70E-05 | 0.018735 |
| piR-mmu-50773373 | 9.568962 | 6.820177 | 7.342682 | 0.473984 | 0.186936 | 0.598006 | 1.70E-05 | 0.018814 |
| piR-mmu-5644081 | 6.435484 | 9.213359 | 6.959113 | 0.800336 | 0.426713 | 0.304733 | 1.71E-05 | 0.018834 |
| piR-mmu-48811585 | 9.360661 | 9.897245 | 12.50077 | 1.407415 | 0.088379 | 0.465778 | 1.71E-05 | 0.018913 |
| piR-mmu-23550216 | 10.59583 | 9.360865 | 7.890852 | 0.372091 | 0.234727 | 0.385293 | 1.72E-05 | 0.019019 |
| piR-mmu-30249863 | 7.741988 | 11.2776 | 9.916662 | 1.599357 | 0.261799 | 0.253491 | 1.75E-05 | 0.019349 |
| piR-mmu-49597541 | 11.26435 | 10.01263 | 8.542848 | 0.350769 | 0.186335 | 0.475092 | 1.79E-05 | 0.019789 |
| piR-mmu-49794365 | 7.195728 | 6.285041 | 10.11637 | 1.729598 | 0.279175 | 0.726797 | 1.81E-05 | 0.020001 |
| piR-mmu-50670137 | 7.628111 | 5.599317 | 4.327988 | 0.845437 | 0.672287 | 0.744504 | 1.85E-05 | 0.020432 |
| piR-mmu-49121139 | 5.772802 | 8.010896 | 6.98259 | 0.560162 | 0.307717 | 0.198206 | 1.95E-05 | 0.021507 |

| | | | | | | | | |
|---|---|---|---|---|---|---|---|---|
| piR-mmu-3753105 | 8.049068 | 6.525187 | 5.074443 | 0.496475 | 0.494875 | 0.589175 | 2.03E-05 | 0.022435 |
| piR-mmu-11220180 | 10.32558 | 7.705047 | 7.9696 | 0.223655 | 0.65727 | 0.570421 | 2.04E-05 | 0.022513 |
| piR-mmu-24893113 | 6.778698 | 9.043916 | 9.273699 | 0.557823 | 0.674377 | 0.027043 | 2.08E-05 | 0.022919 |
| piR-mmu-15757013 | 6.608934 | 9.017957 | 6.339959 | 0.328246 | 0.847314 | 0.253452 | 2.11E-05 | 0.023338 |
| piR-mmu-2678950 | 7.919631 | 6.648886 | 5.099925 | 0.502653 | 0.290963 | 0.527478 | 2.23E-05 | 0.024568 |
| piR-mmu-50773402 | 6.767372 | 9.974545 | 9.164421 | 1.349618 | 0.340071 | 0.447596 | 2.25E-05 | 0.024802 |
| piR-mmu-49279079 | 7.617926 | 9.298544 | 9.898779 | 0.286718 | 0.162208 | 0.34907 | 2.26E-05 | 0.024953 |
| piR-mmu-30125408 | 6.13517 | 10.79343 | 7.9276 | 0.201234 | 0.794917 | 2.587181 | 2.27E-05 | 0.025084 |
| piR-mmu-4955906 | 6.387204 | 7.308338 | 9.374232 | 0.100285 | 0.6349 | 1.092721 | 2.31E-05 | 0.025518 |
| piR-mmu-29300517 | 6.666858 | 9.155887 | 8.068158 | 0.164895 | 0.26823 | 0.52234 | 2.32E-05 | 0.025568 |
| piR-mmu-10870488 | 6.478389 | 7.931631 | 9.130325 | 0.196437 | 0.431678 | 0.507081 | 2.40E-05 | 0.026542 |
| piR-mmu-48965986 | 6.453257 | 7.290597 | 9.432474 | 0.904904 | 0.51517 | 0.629385 | 2.42E-05 | 0.02669 |
| piR-mmu-29244907 | 8.4554 | 6.409087 | 5.235411 | 1.078937 | 0.312244 | 0.339247 | 2.52E-05 | 0.027829 |
| piR-mmu-10937275 | 8.381393 | 5.992826 | 5.4026 | 0.213211 | 0.523297 | 0.847976 | 2.55E-05 | 0.028159 |
| piR-mmu-12634892 | 12.11505 | 9.550065 | 10.11089 | 0.240477 | 0.421147 | 0.487121 | 2.64E-05 | 0.029159 |
| piR-mmu-50282397 | 6.480228 | 9.467626 | 7.896658 | 0.959233 | 0.249862 | 0.160147 | 2.67E-05 | 0.029524 |
| piR-mmu-26503959 | 6.920697 | 9.831993 | 8.962642 | 0.630943 | 0.387061 | 0.533254 | 2.69E-05 | 0.029644 |
| piR-mmu-50734440 | 6.530278 | 7.480803 | 9.15731 | 0.147064 | 0.380526 | 0.360317 | 2.73E-05 | 0.030166 |
| piR-mmu-16410150 | 8.52871 | 5.901432 | 6.093906 | 0.118258 | 0.520639 | 0.349563 | 2.76E-05 | 0.030499 |
| piR-mmu-51321102 | 7.698202 | 10.34535 | 10.02261 | 0.812441 | 0.452908 | 0.16375 | 2.79E-05 | 0.030813 |
| piR-mmu-49006988 | 5.15005 | 7.469701 | 7.14924 | 0.554877 | 0.265423 | 0.195725 | 2.81E-05 | 0.031017 |
| piR-mmu-49949269 | 10.36916 | 8.924893 | 7.588021 | 0.569138 | 0.602105 | 0.658555 | 2.83E-05 | 0.031266 |
| piR-mmu-23825363 | 7.425108 | 10.63169 | 9.616893 | 0.916632 | 0.995404 | 0.515052 | 2.84E-05 | 0.031383 |
| piR-mmu-30081630 | 7.413435 | 6.14883 | 3.826215 | 1.412211 | 0.472058 | 0.78449 | 2.89E-05 | 0.0319 |
| piR-mmu-631775 | 6.882397 | 9.292891 | 9.09487 | 0.355033 | 0.42956 | 0.331719 | 2.90E-05 | 0.032001 |
| piR-mmu-23721480 | 7.500329 | 10.33714 | 10.08761 | 0.738559 | 0.798473 | 0.664086 | 2.94E-05 | 0.03246 |
| piR-mmu-11066915 | 7.505415 | 5.10677 | 4.768957 | 0.494776 | 0.689188 | 0.476739 | 2.96E-05 | 0.032697 |
| piR-mmu-49709935 | 7.156832 | 4.917646 | 4.418427 | 0.36697 | 0.231155 | 0.312046 | 2.96E-05 | 0.032701 |
| piR-mmu-11461028 | 8.830341 | 6.671914 | 5.647616 | 0.220243 | 0.653043 | 1.093104 | 2.98E-05 | 0.03288 |
| piR-mmu-7489023 | 9.519627 | 6.91477 | 7.395858 | 0.331494 | 0.635051 | 0.576882 | 3.20E-05 | 0.035341 |
| piR-mmu-37314969 | 7.242855 | 9.939862 | 7.207731 | 0.49182 | 0.519279 | 1.058546 | 3.24E-05 | 0.035765 |
| piR-mmu-29936651 | 7.627713 | 6.072867 | 4.740996 | 0.945865 | 0.095773 | 0.229958 | 3.34E-05 | 0.036878 |
| piR-mmu-50194882 | 8.369746 | 11.1464 | 9.522836 | 0.982603 | 0.362325 | 0.113948 | 3.45E-05 | 0.038054 |
| piR-mmu-49122465 | 6.96591 | 3.629663 | 3.698056 | 1.096571 | 0.663394 | 0.986572 | 3.48E-05 | 0.038444 |
| piR-mmu-48823448 | 5.250764 | 8.867406 | 6.195424 | 0.471369 | 0.941887 | 1.69773 | 3.51E-05 | 0.038764 |
| piR-mmu-1451695 | 8.052145 | 10.92301 | 8.525845 | 0.962948 | 0.713495 | 0.417678 | 3.53E-05 | 0.03902 |
| piR-mmu-2033913 | 5.52243 | 8.711603 | 6.404922 | 0.385715 | 0.97034 | 1.371163 | 3.79E-05 | 0.041816 |
| piR-mmu-841407 | 8.105439 | 6.503748 | 9.470858 | 0.957873 | 0.633352 | 0.652964 | 3.81E-05 | 0.042029 |
| piR-mmu-51340600 | 9.030951 | 6.634132 | 7.50968 | 0.149847 | 0.181998 | 0.331545 | 3.84E-05 | 0.042445 |

| | | | | | | | | |
|---|---|---|---|---|---|---|---|---|
| piR-mmu-49253747 | 8.552386 | 11.08933 | 10.1604 | 0.367841 | 0.146947 | 0.261643 | 3.86E-05 | 0.042629 |
| piR-mmu-10925255 | 7.741014 | 5.563985 | 5.06083 | 0.144086 | 0.72206 | 0.406777 | 4.14E-05 | 0.045707 |
| piR-mmu-11087008 | 8.460055 | 6.093725 | 8.101463 | 0.344232 | 0.1507 | 0.333849 | 4.15E-05 | 0.04581 |
| piR-mmu-16155104 | 6.488672 | 8.393035 | 9.206275 | 0.770497 | 0.575781 | 0.179456 | 4.25E-05 | 0.046927 |
| piR-mmu-8132128 | 7.124045 | 10.13585 | 9.032629 | 1.116705 | 0.215488 | 0.330003 | 4.28E-05 | 0.047234 |
| piR-mmu-48943025 | 11.85424 | 9.286701 | 10.06978 | 0.209989 | 0.528864 | 0.560219 | 4.29E-05 | 0.047345 |
| piR-mmu-30055696 | 7.405981 | 5.113643 | 5.058356 | 0.235553 | 0.476881 | 0.224867 | 4.31E-05 | 0.047556 |
| piR-mmu-49363871 | 8.870237 | 10.6913 | 12.20091 | 1.47951 | 0.158474 | 0.370064 | 4.45E-05 | 0.049121 |
| piR-mmu-32397906 | 8.961152 | 11.11719 | 8.8911 | 0.163525 | 0.457803 | 0.471408 | 4.50E-05 | 0.049669 |
| piR-mmu-48803219 | 6.951924 | 7.205359 | 9.652404 | 0.907967 | 0.093193 | 0.476719 | 4.52E-05 | 0.049934 |

adj P: Benjamini-Hochberg adjusted P value.

**Supplementary Table S1b: Differentially expressed piRNAs in oral mucosal wound healing**

| | **Mean** | | | **StDev** | | | | |
|---|---|---|---|---|---|---|---|---|
| **piRNA** | 0 hr | 24 hr | 5 day | 0 hr | 24 hr | 5 day | **pVal** | **adj P** |
| piR-mmu-23632402 | 8.545856 | 6.714704 | 5.822723 | 0.567802 | 1.470847 | 0.448796 | 0.003404 | 0.9999 |
| piR-mmu-37120680 | 6.102041 | 7.876522 | 9.352654 | 1.099071 | 1.133759 | 0.799032 | 0.005566 | 0.9999 |
| piR-mmu-12363372 | 7.149341 | 5.751218 | 4.950101 | 0.709467 | 0.510774 | 0.246961 | 0.008648 | 0.9999 |
| piR-mmu-26877685 | 6.40733 | 8.241614 | 9.161494 | 1.505142 | 0.721752 | 0.802004 | 0.008954 | 0.9999 |
| piR-mmu-16071780 | 7.653443 | 8.946332 | 10.218 | 0.375789 | 0.810963 | 0.868781 | 0.009613 | 0.9999 |

adj P: Benjamini-Hochberg adjusted P value.

**Supplementary Table S2: Baseline tissue-specific differentially expressed piRNAs between skin and oral mucosa epithelium**

| | Mean | | StDev | | | |
|---|---|---|---|---|---|---|
| **piRNA** | Skin | Oral | Skin | Oral | **pVal** | **adj P** |
| **piR-mmu-49382307** | 6.011552 | 10.1937 | 0.450553 | 0.241789 | 7.62E-07 | 0.000841 |
| **piR-mmu-8855247** | 5.742301 | 8.926093 | 0.619377 | 0.3768 | 2.64E-05 | 0.014577 |
| **piR-mmu-1996661** | 8.821273 | 12.9794 | 1.240297 | 0.874027 | 5.06E-05 | 0.018629 |
| **piR-mmu-27200780** | 8.000454 | 5.051215 | 0.274638 | 0.87648 | 0.000216 | 0.039285 |
| **piR-mmu-240378** | 15.68287 | 12.8386 | 0.372211 | 1.05009 | 0.000288 | 0.039285 |
| **piR-mmu-4981268** | 9.643152 | 6.157263 | 1.339592 | 0.774927 | 0.000317 | 0.039285 |
| **piR-mmu-49754607** | 10.80484 | 13.56093 | 0.525127 | 0.284167 | 0.000335 | 0.039285 |
| **piR-mmu-24507210** | 9.979987 | 12.73058 | 0.49342 | 0.26704 | 0.000357 | 0.039285 |
| piR-mmu-11461049 | 7.831382 | 4.679713 | 0.164814 | 1.460871 | 0.000487 | 0.039285 |
| piR-mmu-10912700 | 9.019803 | 6.266817 | 0.150037 | 0.987323 | 0.000525 | 0.039285 |
| piR-mmu-49714631 | 7.39974 | 4.727223 | 0.302992 | 0.73737 | 0.000536 | 0.039285 |
| piR-mmu-30876808 | 5.633238 | 3.365039 | 0.221386 | 0.712874 | 0.000538 | 0.039285 |
| piR-mmu-10916133 | 9.006139 | 5.920146 | 0.181429 | 1.301235 | 0.000547 | 0.039285 |
| piR-mmu-49079980 | 8.016324 | 4.485175 | 0.225311 | 1.993919 | 0.000645 | 0.039285 |
| piR-mmu-30053093 | 6.32531 | 3.752345 | 0.280841 | 0.827534 | 0.000648 | 0.039285 |
| piR-mmu-48824062 | 8.268448 | 5.119166 | 0.982995 | 0.8712 | 0.00074 | 0.039285 |
| piR-mmu-8379514 | 6.622364 | 9.147847 | 0.072181 | 0.359798 | 0.000773 | 0.039285 |
| piR-mmu-10934588 | 8.501352 | 5.865535 | 0.155096 | 0.733998 | 0.000806 | 0.039285 |
| piR-mmu-49251782 | 7.52583 | 4.446159 | 1.254668 | 0.672699 | 0.000814 | 0.039285 |
| piR-mmu-10991144 | 8.439477 | 5.399423 | 0.188807 | 1.450519 | 0.000821 | 0.039285 |
| piR-mmu-48806923 | 7.981486 | 5.238404 | 0.345378 | 1.044538 | 0.000844 | 0.039285 |
| piR-mmu-2692796 | 8.133247 | 5.367479 | 0.256382 | 1.006387 | 0.000853 | 0.039285 |
| piR-mmu-2678950 | 7.919631 | 5.363272 | 0.502653 | 0.638986 | 0.000925 | 0.039285 |
| piR-mmu-29426453 | 7.685471 | 10.65773 | 1.098009 | 0.627713 | 0.000996 | 0.039285 |
| piR-mmu-48882277 | 7.945725 | 5.355019 | 0.214656 | 0.828789 | 0.001 | 0.039285 |
| piR-mmu-23866657 | 5.909041 | 3.647083 | 0.481623 | 0.778792 | 0.001024 | 0.039285 |
| piR-mmu-5842895 | 8.103472 | 5.192 | 1.241467 | 0.449323 | 0.001031 | 0.039285 |
| piR-mmu-10991141 | 8.657402 | 6.129678 | 0.158311 | 0.987354 | 0.001075 | 0.039285 |
| piR-mmu-685550 | 12.71685 | 10.2542 | 0.460456 | 0.489091 | 0.001203 | 0.039285 |
| piR-mmu-12633865 | 7.81997 | 5.197765 | 0.641788 | 0.896935 | 0.001215 | 0.039285 |
| piR-mmu-11031895 | 7.58526 | 4.877849 | 0.223864 | 1.239018 | 0.001229 | 0.039285 |
| piR-mmu-3698999 | 6.637311 | 8.733597 | 0.175804 | 0.31401 | 0.00124 | 0.039285 |
| piR-mmu-8492800 | 6.776737 | 9.21786 | 0.886213 | 0.253547 | 0.001241 | 0.039285 |
| piR-mmu-49913296 | 6.892045 | 9.247607 | 0.263656 | 0.486117 | 0.001279 | 0.039285 |
| piR-mmu-10906591 | 16.71583 | 14.57278 | 0.028771 | 0.726801 | 0.001305 | 0.039285 |

| | | | | | | |
|---|---|---|---|---|---|---|
| piR-mmu-3753105 | 8.049068 | 5.295393 | 0.496475 | 1.057556 | 0.001308 | 0.039285 |
| piR-mmu-10940440 | 8.908801 | 6.385735 | 0.204908 | 0.957164 | 0.001325 | 0.039285 |
| piR-mmu-49197585 | 8.442509 | 5.063956 | 0.294622 | 2.008048 | 0.001352 | 0.039285 |
| piR-mmu-2004883 | 7.476168 | 10.22248 | 0.884439 | 0.896389 | 0.001401 | 0.039672 |
| piR-mmu-7474221 | 10.26116 | 12.39335 | 0.422005 | 0.391351 | 0.001488 | 0.039794 |
| piR-mmu-48954623 | 9.36661 | 11.67674 | 0.489552 | 0.291969 | 0.001505 | 0.039794 |
| piR-mmu-49015749 | 12.86147 | 9.930503 | 0.409896 | 1.092517 | 0.001531 | 0.039794 |
| piR-mmu-33199875 | 14.47334 | 12.35931 | 0.30886 | 0.723957 | 0.001662 | 0.039794 |
| piR-mmu-10911823 | 9.170175 | 6.801243 | 0.173396 | 0.826335 | 0.001673 | 0.039794 |
| piR-mmu-49287809 | 9.095436 | 6.763861 | 0.376088 | 0.758288 | 0.001711 | 0.039794 |
| piR-mmu-49342972 | 10.3054 | 6.986643 | 0.317342 | 1.904491 | 0.001751 | 0.039794 |
| piR-mmu-51362692 | 7.039682 | 4.459206 | 0.892062 | 0.421031 | 0.001751 | 0.039794 |
| piR-mmu-8492825 | 6.508086 | 8.877179 | 0.757607 | 0.333444 | 0.001803 | 0.039794 |
| piR-mmu-49659703 | 9.51909 | 6.815889 | 0.379303 | 1.258486 | 0.001823 | 0.039794 |
| piR-mmu-50207618 | 20.52026 | 18.38622 | 0.151505 | 0.74858 | 0.001837 | 0.039794 |
| piR-mmu-8507752 | 8.867652 | 6.004725 | 1.459146 | 0.333503 | 0.001861 | 0.039794 |
| piR-mmu-48844636 | 8.647022 | 10.98257 | 0.124699 | 0.611232 | 0.00194 | 0.039794 |
| piR-mmu-10932261 | 9.169739 | 6.821275 | 0.144462 | 0.774313 | 0.001947 | 0.039794 |
| piR-mmu-11461014 | 8.787251 | 6.140072 | 0.086673 | 1.32298 | 0.002049 | 0.039794 |
| piR-mmu-10953882 | 8.904071 | 6.532893 | 0.148529 | 0.658735 | 0.002054 | 0.039794 |
| piR-mmu-50670137 | 7.628111 | 5.160135 | 0.845437 | 0.534881 | 0.002075 | 0.039794 |
| piR-mmu-9265278 | 6.416931 | 8.671537 | 0.797145 | 0.366448 | 0.002076 | 0.039794 |
| piR-mmu-2201360 | 7.119097 | 10.16291 | 1.067111 | 1.261019 | 0.002091 | 0.039794 |
| piR-mmu-3213041 | 9.083183 | 6.826825 | 0.57272 | 0.498043 | 0.002156 | 0.039947 |
| piR-mmu-10916134 | 8.326815 | 6.107024 | 0.194624 | 0.72042 | 0.002171 | 0.039947 |
| piR-mmu-11461037 | 13.64706 | 11.11304 | 0.900588 | 0.994997 | 0.002246 | 0.04053 |
| piR-mmu-11031886 | 8.487193 | 6.039109 | 0.055488 | 1.096883 | 0.002276 | 0.04053 |
| piR-mmu-10911100 | 9.254792 | 6.758299 | 0.126515 | 1.237273 | 0.002627 | 0.04604 |
| piR-mmu-48831092 | 12.78064 | 10.35412 | 0.344555 | 1.002409 | 0.002716 | 0.046854 |
| piR-mmu-10911632 | 9.008779 | 6.643823 | 0.063744 | 0.781272 | 0.002895 | 0.04872 |
| piR-mmu-49325513 | 7.482259 | 9.797665 | 0.567873 | 0.259003 | 0.002929 | 0.04872 |
| piR-mmu-31033718 | 7.915551 | 5.885577 | 0.482042 | 0.084427 | 0.002957 | 0.04872 |
| piR-mmu-49609791 | 8.498115 | 6.083831 | 0.843415 | 0.837839 | 0.003114 | 0.049367 |
| piR-mmu-10960276 | 8.086195 | 5.822554 | 0.213635 | 0.817765 | 0.003125 | 0.049367 |
| piR-mmu-23832900 | 7.927196 | 5.972949 | 0.228311 | 0.067763 | 0.00313 | 0.049367 |

adj P: Benjamini-Hochberg adjusted P value.

**Supplementary Table S3: Bioinformatics prediction of molecular pathways regulated by tissue-specific differentially expressed piRNAs**

**Oral-specific piRNAs targeted pathways:**

| Pathway identifier | Pathway name | Count | PValue | Fold Enrichment | Bonferroni | Benjamini |
|---|---|---|---|---|---|---|
| mmu03015 | mRNA surveillance pathway | 10 | 1.92E-05 | 6.513380759 | 0.00312994 | 0.00313 |
| mmu04114 | Oocyte meiosis | 8 | 0.001946781 | 4.466318235 | 0.27213124 | 0.146848 |
| mmu04728 | Dopaminergic synapse | 8 | 0.00530134 | 3.733042107 | 0.5795447 | 0.250842 |
| mmu04152 | AMPK signaling pathway | 7 | 0.015898974 | 3.419524898 | 0.92663843 | 0.479564 |
| mmu03013 | RNA transport | 8 | 0.018232865 | 2.942515543 | 0.95018228 | 0.451121 |
| mmu05033 | Nicotine addiction | 4 | 0.025123112 | 6.252845528 | 0.98419432 | 0.499041 |
| mmu05202 | Transcriptional misregulation in cancer | 7 | 0.046995666 | 2.652722345 | 0.99960874 | 0.674006 |
| mmu04550 | Signaling pathways regulating pluripotency of stem cells | 6 | 0.068006886 | 2.718628491 | 0.99998967 | 0.761887 |
| mmu04723 | Retrograde endocannabinoid signaling | 5 | 0.080677513 | 3.035361907 | 0.99999889 | 0.782047 |
| mmu05031 | Amphetamine addiction | 4 | 0.089923651 | 3.733042107 | 0.99999979 | 0.784737 |

**Skin-specific piRNAs targeted pathways:**

| Pathway identifier | Pathway name | Count | PValue | Fold Enrichment | Bonferroni | Benjamini |
|---|---|---|---|---|---|---|
| mmu05211 | Renal cell carcinoma | 13 | 2.58E-05 | 4.45557041 | 0.00644912 | 0.006449 |
| mmu04660 | T cell receptor signaling pathway | 14 | 3.44E-04 | 3.230543054 | 0.08266099 | 0.042222 |
| mmu04360 | Axon guidance | 14 | 0.003386471 | 2.529339911 | 0.57320302 | 0.247095 |
| mmu04010 | MAPK signaling pathway | 21 | 0.005309027 | 1.949909453 | 0.73713526 | 0.283967 |
| mmu04014 | Ras signaling pathway | 19 | 0.00913617 | 1.933690618 | 0.90011327 | 0.369186 |
| mmu03040 | Spliceosome | 13 | 0.011338142 | 2.278035999 | 0.9428531 | 0.379371 |
| mmu04722 | Neurotrophin signaling pathway | 12 | 0.015080351 | 2.292399404 | 0.97794022 | 0.420075 |
| mmu05030 | Cocaine addiction | 7 | 0.017363952 | 3.329437229 | 0.98768127 | 0.422807 |
| mmu05205 | Proteoglycans in cancer | 16 | 0.027254184 | 1.836930885 | 0.99902761 | 0.537283 |
| mmu04510 | Focal adhesion | 16 | 0.031694782 | 1.801434636 | 0.99969162 | 0.554438 |
| mmu04012 | ErbB signaling pathway | 9 | 0.032064673 | 2.410971787 | 0.99971982 | 0.524622 |
| mmu04730 | Long-term depression | 7 | 0.045189286 | 2.674465971 | 0.9999909 | 0.619866 |
| mmu04713 | Circadian entrainment | 9 | 0.058031972 | 2.140352505 | 0.9999997 | 0.684719 |
| mmu04720 | Long-term potentiation | 7 | 0.06211028 | 2.471854913 | 0.9999999 | 0.683247 |
| mmu04550 | Signaling pathways regulating pluripotency of stem cells | 11 | 0.071188107 | 1.857729469 | 0.99999999 | 0.709381 |
| mmu04024 | cAMP signaling pathway | 14 | 0.079323864 | 1.656268266 | 1 | 0.726519 |
| mmu04974 | Protein digestion and absorption | 8 | 0.081927452 | 2.118732782 | 1 | 0.716933 |
| mmu04520 | Adherens junction | 7 | 0.086749456 | 2.265867003 | 1 | 0.71787 |
| mmu04022 | cGMP-PKG signaling pathway | 12 | 0.089678898 | 1.715783603 | 1 | 0.710972 |
| mmu04144 | Endocytosis | 17 | 0.093086673 | 1.518019273 | 1 | 0.706606 |
| mmu05202 | Transcriptional misregulation in cancer | 12 | 0.09569783 | 1.694986226 | 1 | 0.699501 |

70 tissue-specific differentially expressed piRNAs (including 18-oral specific and 52 skin-specific piRNAs) were used for the analysis.
The piRNA target gene prediction was performed with MR-microT (aggregated score ≥ 0.9).
A total of 492 and 1869 piRNA-target gene pairs (365 and 1023 non-redundant target genes) were predicted for oral- and skin-specific piRNAs.
The pathway analysis was performed with with DAVID (v6.8).

**Supplementary Table S4a: Bioinformatics prediction piRNA-target gene pairs of oral-specific differentially expressed piRNAs**

| piRNA | target gene | MR-microT score |
|---|---|---|
| piR-mmu-49382307 | Ythdf2 | 0.954893 |
| piR-mmu-49382307 | Ccdc150 | 0.92895 |
| piR-mmu-49382307 | Setd5 | 0.919205 |
| piR-mmu-8855247 | Ythdf2 | 0.954893 |
| piR-mmu-8855247 | Ccdc150 | 0.92895 |
| piR-mmu-8855247 | Setd5 | 0.919205 |
| piR-mmu-1996661 | Fam114a2 | 0.990383 |
| piR-mmu-1996661 | Gm8247 | 0.974847 |
| piR-mmu-1996661 | Ahdc1 | 0.973333 |
| piR-mmu-1996661 | Serpini1 | 0.971592 |
| piR-mmu-1996661 | Hnrnpd | 0.963839 |
| piR-mmu-1996661 | Fbxo28 | 0.961341 |
| piR-mmu-1996661 | Prdm6 | 0.958353 |
| piR-mmu-1996661 | Ppp1cb | 0.958094 |
| piR-mmu-1996661 | Kcnh2 | 0.95561 |
| piR-mmu-1996661 | Kdm7a | 0.95059 |
| piR-mmu-1996661 | Anapc2 | 0.948216 |
| piR-mmu-1996661 | E2f7 | 0.94477 |
| piR-mmu-1996661 | D830030K20Rik | 0.93071 |
| piR-mmu-1996661 | Kif5a | 0.929627 |
| piR-mmu-1996661 | Ppp2r5e | 0.925427 |
| piR-mmu-1996661 | Vkorc1l1 | 0.925096 |
| piR-mmu-1996661 | Relb | 0.9248 |
| piR-mmu-1996661 | Sympk | 0.921113 |
| piR-mmu-1996661 | Gm3278 | 0.915405 |
| piR-mmu-1996661 | Ptbp3 | 0.915041 |
| piR-mmu-1996661 | Gm8271 | 0.913931 |
| piR-mmu-1996661 | Gm3532 | 0.912902 |
| piR-mmu-1996661 | Fip1l1 | 0.909043 |
| piR-mmu-1996661 | Celf4 | 0.904977 |
| piR-mmu-1996661 | Ppp2r5c | 0.902804 |
| piR-mmu-1996661 | Ephb1 | 0.901025 |
| piR-mmu-2201360 | 5430402E10Rik | 0.995845 |
| piR-mmu-2201360 | Gm14744 | 0.994925 |
| piR-mmu-2201360 | Ago2 | 0.99272 |
| piR-mmu-2201360 | BC052040 | 0.986589 |
| piR-mmu-2201360 | Serpini1 | 0.968061 |
| piR-mmu-2201360 | Ahdc1 | 0.958687 |
| piR-mmu-2201360 | Ppp1cb | 0.95719 |
| piR-mmu-2201360 | Gm8247 | 0.956703 |
| piR-mmu-2201360 | Abcg8 | 0.953005 |
| piR-mmu-2201360 | Ralgps2 | 0.950137 |
| piR-mmu-2201360 | Bcl9 | 0.946818 |
| piR-mmu-2201360 | Rag2 | 0.942341 |
| piR-mmu-2201360 | Ptbp3 | 0.940046 |
| piR-mmu-2201360 | Nek11 | 0.936471 |
| piR-mmu-2201360 | Clip4 | 0.934483 |
| piR-mmu-2201360 | Cast | 0.934357 |

| | | |
|---|---|---|
| piR-mmu-2201360 | Nckap1l | 0.931753 |
| piR-mmu-2201360 | E2f7 | 0.928199 |
| piR-mmu-2201360 | Btg3 | 0.926763 |
| piR-mmu-2201360 | Mettl3 | 0.925947 |
| piR-mmu-2201360 | Smg1 | 0.925347 |
| piR-mmu-2201360 | Gmeb1 | 0.923545 |
| piR-mmu-2201360 | Upf3b | 0.923513 |
| piR-mmu-2201360 | Zfp160 | 0.923477 |
| piR-mmu-2201360 | Kif5a | 0.922425 |
| piR-mmu-2201360 | Esco1 | 0.922373 |
| piR-mmu-2201360 | Hnrnpd | 0.920936 |
| piR-mmu-2201360 | Ltbp2 | 0.92074 |
| piR-mmu-2201360 | Frmpd4 | 0.92023 |
| piR-mmu-2201360 | Spcs2 | 0.919436 |
| piR-mmu-2201360 | Kdm5c | 0.918879 |
| piR-mmu-2201360 | Esrrg | 0.917279 |
| piR-mmu-2201360 | Rbm15 | 0.915681 |
| piR-mmu-2201360 | Cep350 | 0.915337 |
| piR-mmu-2201360 | Pomt2 | 0.913058 |
| piR-mmu-2201360 | Foxj3 | 0.910785 |
| piR-mmu-2201360 | Ctbs | 0.910095 |
| piR-mmu-2201360 | Kcnh2 | 0.908137 |
| piR-mmu-2201360 | Iffo2 | 0.903367 |
| piR-mmu-2201360 | Chchd3 | 0.902839 |
| piR-mmu-2201360 | Mecp2 | 0.900381 |
| piR-mmu-29426453 | Pnisr | 0.997744 |
| piR-mmu-29426453 | Plppr4 | 0.990007 |
| piR-mmu-29426453 | Cpeb4 | 0.986842 |
| piR-mmu-29426453 | Necab1 | 0.984047 |
| piR-mmu-29426453 | Mmp13 | 0.983959 |
| piR-mmu-29426453 | Dazl | 0.981887 |
| piR-mmu-29426453 | Prpf40a | 0.981398 |
| piR-mmu-29426453 | Crisp3 | 0.981371 |
| piR-mmu-29426453 | Zfp800 | 0.98116 |
| piR-mmu-29426453 | Mars2 | 0.98088 |
| piR-mmu-29426453 | C77370 | 0.980861 |
| piR-mmu-29426453 | Fmr1 | 0.979981 |
| piR-mmu-29426453 | Ptbp3 | 0.978896 |
| piR-mmu-29426453 | Phc3 | 0.978033 |
| piR-mmu-29426453 | Whsc1 | 0.978018 |
| piR-mmu-29426453 | Gch1 | 0.977015 |
| piR-mmu-29426453 | Meioc | 0.976434 |
| piR-mmu-29426453 | Phip | 0.973197 |
| piR-mmu-29426453 | Fermt2 | 0.971877 |
| piR-mmu-29426453 | Foxn2 | 0.967597 |
| piR-mmu-29426453 | Pura | 0.965832 |
| piR-mmu-29426453 | Ube2v2 | 0.964946 |
| piR-mmu-29426453 | Ism1 | 0.963961 |
| piR-mmu-29426453 | Taok1 | 0.963654 |
| piR-mmu-29426453 | Mib1 | 0.963589 |
| piR-mmu-29426453 | Fubp1 | 0.960643 |
| piR-mmu-29426453 | Tead1 | 0.960634 |

| | | |
|---|---|---|
| piR-mmu-29426453 | D1Ertd622e | 0.958517 |
| piR-mmu-29426453 | Syncrip | 0.958444 |
| piR-mmu-29426453 | Prex2 | 0.957806 |
| piR-mmu-29426453 | Gria3 | 0.957119 |
| piR-mmu-29426453 | Ppp4r2 | 0.954231 |
| piR-mmu-29426453 | Zmat1 | 0.954155 |
| piR-mmu-29426453 | Smg1 | 0.954101 |
| piR-mmu-29426453 | Iqch | 0.950594 |
| piR-mmu-29426453 | Lmbrd1 | 0.950446 |
| piR-mmu-29426453 | Zbtb20 | 0.949713 |
| piR-mmu-29426453 | Crebrf | 0.949684 |
| piR-mmu-29426453 | Chrnb4 | 0.949138 |
| piR-mmu-29426453 | Ivns1abp | 0.947193 |
| piR-mmu-29426453 | Fam199x | 0.947102 |
| piR-mmu-29426453 | Gm20537 | 0.944916 |
| piR-mmu-29426453 | 5430402E10Rik | 0.944509 |
| piR-mmu-29426453 | Ect2 | 0.943553 |
| piR-mmu-29426453 | Rlim | 0.939342 |
| piR-mmu-29426453 | Chic2 | 0.938787 |
| piR-mmu-29426453 | Hist2h2be | 0.938729 |
| piR-mmu-29426453 | Nexn | 0.938445 |
| piR-mmu-29426453 | Ptprd | 0.93733 |
| piR-mmu-29426453 | Nsa2 | 0.936769 |
| piR-mmu-29426453 | Cadm2 | 0.936064 |
| piR-mmu-29426453 | 1700010B08Rik | 0.935885 |
| piR-mmu-29426453 | Spred1 | 0.935736 |
| piR-mmu-29426453 | Rabep1 | 0.933335 |
| piR-mmu-29426453 | Acadl | 0.933057 |
| piR-mmu-29426453 | Elavl1 | 0.932677 |
| piR-mmu-29426453 | Myh8 | 0.932655 |
| piR-mmu-29426453 | Lats1 | 0.932408 |
| piR-mmu-29426453 | Srgn | 0.931822 |
| piR-mmu-29426453 | C1galt1 | 0.931777 |
| piR-mmu-29426453 | Arhgap11a | 0.929846 |
| piR-mmu-29426453 | Xpo1 | 0.929497 |
| piR-mmu-29426453 | Tgfbr1 | 0.928897 |
| piR-mmu-29426453 | Fus | 0.927475 |
| piR-mmu-29426453 | Hnrnpr | 0.925989 |
| piR-mmu-29426453 | 1700066M21Rik | 0.925046 |
| piR-mmu-29426453 | Cdkl5 | 0.925045 |
| piR-mmu-29426453 | Rfesd | 0.921883 |
| piR-mmu-29426453 | Srsf3 | 0.920642 |
| piR-mmu-29426453 | Slmap | 0.920134 |
| piR-mmu-29426453 | Slco1a1 | 0.919091 |
| piR-mmu-29426453 | Ptpn12 | 0.918157 |
| piR-mmu-29426453 | Gfral | 0.917667 |
| piR-mmu-29426453 | Fsbp | 0.917211 |
| piR-mmu-29426453 | Spopl | 0.914839 |
| piR-mmu-29426453 | Ildr2 | 0.914724 |
| piR-mmu-29426453 | Kcnh7 | 0.914711 |
| piR-mmu-29426453 | Ak6 | 0.914574 |
| piR-mmu-29426453 | Kdm7a | 0.914098 |

| | | |
|---|---|---|
| piR-mmu-29426453 | Gm19965 | 0.914058 |
| piR-mmu-29426453 | Cdc73 | 0.912351 |
| piR-mmu-29426453 | Rpgrip1l | 0.912189 |
| piR-mmu-29426453 | Dopey1 | 0.911902 |
| piR-mmu-29426453 | March6 | 0.910348 |
| piR-mmu-29426453 | Unk | 0.910099 |
| piR-mmu-29426453 | Mef2d | 0.909795 |
| piR-mmu-29426453 | Dpp10 | 0.909666 |
| piR-mmu-29426453 | Wdr43 | 0.905411 |
| piR-mmu-29426453 | Mgat4c | 0.904272 |
| piR-mmu-29426453 | Tmem215 | 0.904134 |
| piR-mmu-29426453 | St8sia4 | 0.903042 |
| piR-mmu-29426453 | Bend6 | 0.903039 |
| piR-mmu-29426453 | Med13 | 0.902643 |
| piR-mmu-29426453 | Rab30 | 0.902439 |
| piR-mmu-29426453 | App | 0.902297 |
| piR-mmu-29426453 | Sirt1 | 0.901677 |
| piR-mmu-29426453 | Gm5415 | 0.90165 |
| piR-mmu-29426453 | Enah | 0.9003 |
| piR-mmu-29426453 | Mysm1 | 0.900138 |
| piR-mmu-8379514 | Supt16 | 0.9397 |
| piR-mmu-2004883 | Fbxo28 | 0.99105 |
| piR-mmu-2004883 | Hnrnpd | 0.956761 |
| piR-mmu-2004883 | Ppp2r5e | 0.930728 |
| piR-mmu-2004883 | Pcm1 | 0.921105 |
| piR-mmu-2004883 | Eif2ak3 | 0.911102 |
| piR-mmu-2004883 | Eme1 | 0.907139 |
| piR-mmu-8492825 | Zfp704 | 0.979934 |
| piR-mmu-8492825 | Pura | 0.967762 |
| piR-mmu-8492825 | Ccdc120 | 0.958503 |
| piR-mmu-8492825 | Gm10282 | 0.955684 |
| piR-mmu-8492825 | D1Ertd622e | 0.954168 |
| piR-mmu-8492825 | Ctu1 | 0.953616 |
| piR-mmu-8492825 | Megf11 | 0.952288 |
| piR-mmu-8492825 | Zbtb4 | 0.951919 |
| piR-mmu-8492825 | Tmem151a | 0.951816 |
| piR-mmu-8492825 | Fam149b | 0.948346 |
| piR-mmu-8492825 | Ddx6 | 0.946188 |
| piR-mmu-8492825 | Tbc1d12 | 0.943482 |
| piR-mmu-8492825 | Ccnc | 0.943008 |
| piR-mmu-8492825 | Taok1 | 0.94156 |
| piR-mmu-8492825 | Camk1d | 0.937831 |
| piR-mmu-8492825 | Trip4 | 0.934576 |
| piR-mmu-8492825 | Ddx55 | 0.929028 |
| piR-mmu-8492825 | Id4 | 0.923827 |
| piR-mmu-8492825 | Cpeb4 | 0.921971 |
| piR-mmu-8492825 | Mef2d | 0.921487 |
| piR-mmu-8492825 | Phf3 | 0.919968 |
| piR-mmu-8492825 | Plagl1 | 0.918111 |
| piR-mmu-8492825 | Zfp740 | 0.915668 |
| piR-mmu-8492825 | Itga10 | 0.914853 |
| piR-mmu-8492825 | Zeb2 | 0.913046 |

| | | |
|---|---|---|
| piR-mmu-8492825 | 4932438A13Rik | 0.912683 |
| piR-mmu-8492825 | Ube2k | 0.907627 |
| piR-mmu-8492825 | Mtmr4 | 0.905144 |
| piR-mmu-8492825 | 4930452B06Rik | 0.904951 |
| piR-mmu-8492825 | Mier3 | 0.903886 |
| piR-mmu-8492825 | Umad1 | 0.900682 |
| piR-mmu-8492800 | Zfp704 | 0.979985 |
| piR-mmu-8492800 | Pura | 0.967635 |
| piR-mmu-8492800 | Ccdc120 | 0.958057 |
| piR-mmu-8492800 | Gm10282 | 0.955881 |
| piR-mmu-8492800 | D1Ertd622e | 0.954095 |
| piR-mmu-8492800 | Ctu1 | 0.953616 |
| piR-mmu-8492800 | Megf11 | 0.952288 |
| piR-mmu-8492800 | Zbtb4 | 0.951227 |
| piR-mmu-8492800 | Tmem151a | 0.950984 |
| piR-mmu-8492800 | Fam149b | 0.948346 |
| piR-mmu-8492800 | Ddx6 | 0.946277 |
| piR-mmu-8492800 | Tbc1d12 | 0.943482 |
| piR-mmu-8492800 | Ccnc | 0.943008 |
| piR-mmu-8492800 | Taok1 | 0.941571 |
| piR-mmu-8492800 | Camk1d | 0.937859 |
| piR-mmu-8492800 | Trip4 | 0.931963 |
| piR-mmu-8492800 | Ddx55 | 0.929378 |
| piR-mmu-8492800 | Cpeb4 | 0.921971 |
| piR-mmu-8492800 | Mef2d | 0.921525 |
| piR-mmu-8492800 | Id4 | 0.920398 |
| piR-mmu-8492800 | Plagl1 | 0.918111 |
| piR-mmu-8492800 | Zeb2 | 0.917569 |
| piR-mmu-8492800 | Phf3 | 0.91555 |
| piR-mmu-8492800 | Zfp740 | 0.915116 |
| piR-mmu-8492800 | Itga10 | 0.915028 |
| piR-mmu-8492800 | 4932438A13Rik | 0.911072 |
| piR-mmu-8492800 | Ube2k | 0.907504 |
| piR-mmu-8492800 | 4930452B06Rik | 0.904951 |
| piR-mmu-8492800 | Mier3 | 0.901547 |
| piR-mmu-8492800 | Mtmr4 | 0.901232 |
| piR-mmu-8492800 | Umad1 | 0.900682 |
| piR-mmu-9265278 | Papolg | 0.994918 |
| piR-mmu-9265278 | Tmem50a | 0.990819 |
| piR-mmu-9265278 | Bbx | 0.990012 |
| piR-mmu-9265278 | Cbl | 0.988102 |
| piR-mmu-9265278 | Brd1 | 0.987071 |
| piR-mmu-9265278 | Mtf2 | 0.985754 |
| piR-mmu-9265278 | Wasf2 | 0.983942 |
| piR-mmu-9265278 | Sbf1 | 0.982674 |
| piR-mmu-9265278 | Zfyve26 | 0.981194 |
| piR-mmu-9265278 | Arhgef3 | 0.972462 |
| piR-mmu-9265278 | Nfia | 0.969373 |
| piR-mmu-9265278 | Cdkl5 | 0.968797 |
| piR-mmu-9265278 | Abcc5 | 0.968504 |
| piR-mmu-9265278 | Psmf1 | 0.967593 |
| piR-mmu-9265278 | Lce1c | 0.967268 |

| | | |
|---|---|---|
| piR-mmu-9265278 | Zbtb20 | 0.96704 |
| piR-mmu-9265278 | Cisd1 | 0.962676 |
| piR-mmu-9265278 | Hrh2 | 0.962431 |
| piR-mmu-9265278 | Bdnf | 0.962294 |
| piR-mmu-9265278 | Tph1 | 0.959649 |
| piR-mmu-9265278 | Lce1f | 0.956302 |
| piR-mmu-9265278 | Acin1 | 0.956182 |
| piR-mmu-9265278 | Tmem132a | 0.953808 |
| piR-mmu-9265278 | Agps | 0.951753 |
| piR-mmu-9265278 | Lce1a1 | 0.949468 |
| piR-mmu-9265278 | Foxk1 | 0.947535 |
| piR-mmu-9265278 | Mphosph9 | 0.94735 |
| piR-mmu-9265278 | Prkag2 | 0.947298 |
| piR-mmu-9265278 | Slc22a23 | 0.947012 |
| piR-mmu-9265278 | Ppp1r1c | 0.944525 |
| piR-mmu-9265278 | Zzz3 | 0.942763 |
| piR-mmu-9265278 | Dhx40 | 0.942626 |
| piR-mmu-9265278 | Plrg1 | 0.94048 |
| piR-mmu-9265278 | Lce1a2 | 0.940287 |
| piR-mmu-9265278 | Fgfr2 | 0.939736 |
| piR-mmu-9265278 | Cdon | 0.939438 |
| piR-mmu-9265278 | Fosl2 | 0.93484 |
| piR-mmu-9265278 | Thoc1 | 0.933294 |
| piR-mmu-9265278 | Chd9 | 0.932579 |
| piR-mmu-9265278 | Myocd | 0.93084 |
| piR-mmu-9265278 | Lce1g | 0.930125 |
| piR-mmu-9265278 | Susd6 | 0.928422 |
| piR-mmu-9265278 | Slc25a46 | 0.92776 |
| piR-mmu-9265278 | Tnrc6b | 0.927733 |
| piR-mmu-9265278 | Trmt2b | 0.927293 |
| piR-mmu-9265278 | Nol4l | 0.92634 |
| piR-mmu-9265278 | Fam122b | 0.9262 |
| piR-mmu-9265278 | Mier1 | 0.922623 |
| piR-mmu-9265278 | Dcstamp | 0.92204 |
| piR-mmu-9265278 | Krt76 | 0.920829 |
| piR-mmu-9265278 | Reps2 | 0.920275 |
| piR-mmu-9265278 | Mafg | 0.919547 |
| piR-mmu-9265278 | Lce1b | 0.919156 |
| piR-mmu-9265278 | Ppm1g | 0.918733 |
| piR-mmu-9265278 | Adrbk1 | 0.918521 |
| piR-mmu-9265278 | Sdc2 | 0.915272 |
| piR-mmu-9265278 | Cacna1b | 0.915061 |
| piR-mmu-9265278 | Rnf6 | 0.914496 |
| piR-mmu-9265278 | Adamts5 | 0.910052 |
| piR-mmu-9265278 | Entpd3 | 0.90875 |
| piR-mmu-9265278 | Adam9 | 0.906222 |
| piR-mmu-9265278 | Cfap100 | 0.906028 |
| piR-mmu-9265278 | D1Ertd622e | 0.904846 |
| piR-mmu-9265278 | Ppp4c | 0.902119 |
| piR-mmu-9265278 | Nfib | 0.901654 |
| piR-mmu-9265278 | Ptcd1 | 0.901609 |
| piR-mmu-9265278 | Son | 0.900922 |

| | | |
|---|---|---|
| piR-mmu-9265278 | Tanc2 | 0.900243 |
| piR-mmu-49913296 | Gm7293 | 0.994585 |
| piR-mmu-49913296 | Fam120a | 0.986716 |
| piR-mmu-49913296 | Khdc1a | 0.986557 |
| piR-mmu-49913296 | Rnf115 | 0.983542 |
| piR-mmu-49913296 | Gria2 | 0.979262 |
| piR-mmu-49913296 | Cd46 | 0.972232 |
| piR-mmu-49913296 | E130114P18Rik | 0.971622 |
| piR-mmu-49913296 | Kctd8 | 0.96546 |
| piR-mmu-49913296 | Snx7 | 0.964454 |
| piR-mmu-49913296 | Gabrb2 | 0.962885 |
| piR-mmu-49913296 | Inpp4a | 0.953382 |
| piR-mmu-49913296 | Oraov1 | 0.942119 |
| piR-mmu-49913296 | Dip2c | 0.941789 |
| piR-mmu-49913296 | Zfp810 | 0.93942 |
| piR-mmu-49913296 | Ssfa2 | 0.936781 |
| piR-mmu-49913296 | Sumo2 | 0.932753 |
| piR-mmu-49913296 | Ogt | 0.931633 |
| piR-mmu-49913296 | Edn1 | 0.931457 |
| piR-mmu-49913296 | Trim63 | 0.928694 |
| piR-mmu-49913296 | Glis3 | 0.92283 |
| piR-mmu-49913296 | Parp6 | 0.922825 |
| piR-mmu-49913296 | Hmga2 | 0.921754 |
| piR-mmu-49913296 | AF529169 | 0.921189 |
| piR-mmu-49913296 | Tanc2 | 0.919253 |
| piR-mmu-49913296 | Skap1 | 0.919214 |
| piR-mmu-49913296 | Atp2b1 | 0.918548 |
| piR-mmu-49913296 | Dmd | 0.912054 |
| piR-mmu-49913296 | Trpm8 | 0.910737 |
| piR-mmu-49913296 | Cadm2 | 0.90721 |
| piR-mmu-49913296 | Gspt1 | 0.906565 |
| piR-mmu-49913296 | Dagla | 0.906072 |
| piR-mmu-49913296 | Adamts5 | 0.905675 |
| piR-mmu-49913296 | Kif5b | 0.90512 |
| piR-mmu-49913296 | Csmd3 | 0.904569 |
| piR-mmu-3698999 | Magt1 | 0.999311 |
| piR-mmu-3698999 | Dctn2 | 0.99304 |
| piR-mmu-3698999 | Zfand1 | 0.988632 |
| piR-mmu-3698999 | Rdm1 | 0.988223 |
| piR-mmu-3698999 | Tead1 | 0.987853 |
| piR-mmu-3698999 | Gtpbp8 | 0.981519 |
| piR-mmu-3698999 | Crebrf | 0.973687 |
| piR-mmu-3698999 | Kif5b | 0.972618 |
| piR-mmu-3698999 | Psmd9 | 0.963301 |
| piR-mmu-3698999 | Ccdc163 | 0.954861 |
| piR-mmu-3698999 | Abcd4 | 0.95439 |
| piR-mmu-3698999 | Runx1t1 | 0.946914 |
| piR-mmu-3698999 | Zfp141 | 0.946255 |
| piR-mmu-3698999 | Cbfb | 0.943523 |
| piR-mmu-3698999 | Fblim1 | 0.936822 |
| piR-mmu-3698999 | Zfp553 | 0.93565 |
| piR-mmu-3698999 | Ccdc125 | 0.932996 |

| | | |
|---|---|---|
| piR-mmu-3698999 | Zmym5 | 0.929406 |
| piR-mmu-3698999 | Inip | 0.928382 |
| piR-mmu-3698999 | Ropn1 | 0.924617 |
| piR-mmu-3698999 | Mrpl33 | 0.921706 |
| piR-mmu-3698999 | Tprkb | 0.918157 |
| piR-mmu-3698999 | Pdcd5 | 0.91513 |
| piR-mmu-3698999 | Rcsd1 | 0.91171 |
| piR-mmu-3698999 | Fxn | 0.906458 |
| piR-mmu-3698999 | Gm4924 | 0.905104 |
| piR-mmu-3698999 | Ankrd24 | 0.902011 |
| piR-mmu-49325513 | 4930474N05Rik | 0.964632 |
| piR-mmu-49325513 | Zfp521 | 0.960943 |
| piR-mmu-49325513 | Gm4631 | 0.927372 |
| piR-mmu-49325513 | Ttn | 0.903327 |
| piR-mmu-49325513 | Rab21 | 0.903086 |
| piR-mmu-49325513 | Kpna4 | 0.900726 |
| piR-mmu-24507210 | Ddx3x | 0.989052 |
| piR-mmu-24507210 | Ogt | 0.980044 |
| piR-mmu-24507210 | Gm15140 | 0.961388 |
| piR-mmu-24507210 | Pcdhb9 | 0.961295 |
| piR-mmu-24507210 | Etnk1 | 0.960515 |
| piR-mmu-24507210 | Gclm | 0.959233 |
| piR-mmu-24507210 | Dennd5b | 0.957864 |
| piR-mmu-24507210 | Rita1 | 0.949149 |
| piR-mmu-24507210 | Nfat5 | 0.948381 |
| piR-mmu-24507210 | Igf1r | 0.947725 |
| piR-mmu-24507210 | Tmem251 | 0.943091 |
| piR-mmu-24507210 | Plcz1 | 0.937915 |
| piR-mmu-24507210 | Ptprd | 0.937253 |
| piR-mmu-24507210 | Zfand3 | 0.935923 |
| piR-mmu-24507210 | Zak | 0.933998 |
| piR-mmu-24507210 | Ldah | 0.933742 |
| piR-mmu-24507210 | Atp1a2 | 0.933501 |
| piR-mmu-24507210 | Ctdspl2 | 0.931958 |
| piR-mmu-24507210 | Rgs17 | 0.927617 |
| piR-mmu-24507210 | Lcp2 | 0.927267 |
| piR-mmu-24507210 | Mxi1 | 0.925051 |
| piR-mmu-24507210 | Trim36 | 0.921167 |
| piR-mmu-24507210 | Meis1 | 0.918076 |
| piR-mmu-24507210 | Fam134a | 0.918041 |
| piR-mmu-24507210 | Anapc11 | 0.916558 |
| piR-mmu-24507210 | D1Ertd622e | 0.915287 |
| piR-mmu-24507210 | Onecut1 | 0.913933 |
| piR-mmu-24507210 | Rnf217 | 0.910694 |
| piR-mmu-24507210 | Anks4b | 0.909092 |
| piR-mmu-24507210 | Wnt7a | 0.906525 |
| piR-mmu-24507210 | Lrriq4 | 0.904059 |
| piR-mmu-24507210 | Zbtb20 | 0.903393 |
| piR-mmu-24507210 | Strap | 0.903003 |
| piR-mmu-24507210 | Amfr | 0.902131 |
| piR-mmu-48844636 | Ddx3x | 0.988867 |
| piR-mmu-48844636 | Ogt | 0.980247 |

| | | |
|---|---|---|
| piR-mmu-48844636 | Gm15140 | 0.961388 |
| piR-mmu-48844636 | Pcdhb9 | 0.961295 |
| piR-mmu-48844636 | Gclm | 0.961234 |
| piR-mmu-48844636 | Etnk1 | 0.960495 |
| piR-mmu-48844636 | Dennd5b | 0.957863 |
| piR-mmu-48844636 | Rita1 | 0.949149 |
| piR-mmu-48844636 | Nfat5 | 0.948336 |
| piR-mmu-48844636 | Igf1r | 0.947715 |
| piR-mmu-48844636 | Tmem251 | 0.943091 |
| piR-mmu-48844636 | Plcz1 | 0.938602 |
| piR-mmu-48844636 | Ptprd | 0.937152 |
| piR-mmu-48844636 | Zfand3 | 0.935923 |
| piR-mmu-48844636 | Atp1a2 | 0.934381 |
| piR-mmu-48844636 | Zak | 0.933998 |
| piR-mmu-48844636 | Ldah | 0.933742 |
| piR-mmu-48844636 | Ctdspl2 | 0.931958 |
| piR-mmu-48844636 | Rgs17 | 0.927386 |
| piR-mmu-48844636 | Lcp2 | 0.927267 |
| piR-mmu-48844636 | Mxi1 | 0.925051 |
| piR-mmu-48844636 | Trim36 | 0.921167 |
| piR-mmu-48844636 | Meis1 | 0.918076 |
| piR-mmu-48844636 | Fam134a | 0.918041 |
| piR-mmu-48844636 | Anapc11 | 0.916531 |
| piR-mmu-48844636 | D1Ertd622e | 0.915339 |
| piR-mmu-48844636 | Wnt7a | 0.914798 |
| piR-mmu-48844636 | Onecut1 | 0.913933 |
| piR-mmu-48844636 | Rnf217 | 0.910694 |
| piR-mmu-48844636 | Anks4b | 0.909092 |
| piR-mmu-48844636 | Lrriq4 | 0.904059 |
| piR-mmu-48844636 | Zbtb20 | 0.90345 |
| piR-mmu-48844636 | Strap | 0.903003 |
| piR-mmu-48844636 | Amfr | 0.902131 |
| piR-mmu-49754607 | Gm7293 | 0.994491 |
| piR-mmu-49754607 | E130114P18Rik | 0.968933 |
| piR-mmu-49754607 | Fam120a | 0.957455 |
| piR-mmu-49754607 | Gabrb2 | 0.954991 |
| piR-mmu-49754607 | Jph1 | 0.946742 |
| piR-mmu-49754607 | Snx7 | 0.936162 |
| piR-mmu-49754607 | Skap1 | 0.929981 |
| piR-mmu-49754607 | Trpm8 | 0.926452 |
| piR-mmu-49754607 | Gspt1 | 0.921665 |
| piR-mmu-49754607 | Ptprb | 0.919221 |
| piR-mmu-49754607 | Oraov1 | 0.918198 |
| piR-mmu-49754607 | Parp6 | 0.916909 |
| piR-mmu-49754607 | Gria2 | 0.911238 |
| piR-mmu-49754607 | Csmd3 | 0.9075 |
| piR-mmu-49754607 | Speer3 | 0.905922 |
| piR-mmu-49754607 | Cadm2 | 0.905403 |
| piR-mmu-49754607 | Ncaph2 | 0.904701 |
| piR-mmu-48954623 | Gm7293 | 0.994975 |
| piR-mmu-48954623 | F830016B08Rik | 0.98034 |
| piR-mmu-48954623 | Rps6kb1 | 0.977955 |

| | | |
|---|---|---|
| piR-mmu-48954623 | Cadm2 | 0.967846 |
| piR-mmu-48954623 | Kank4 | 0.962409 |
| piR-mmu-48954623 | Nrep | 0.959476 |
| piR-mmu-48954623 | Rab11a | 0.956309 |
| piR-mmu-48954623 | Mbd3l2 | 0.954699 |
| piR-mmu-48954623 | Tanc2 | 0.950601 |
| piR-mmu-48954623 | Rc3h1 | 0.93603 |
| piR-mmu-48954623 | Ulbp1 | 0.92328 |
| piR-mmu-48954623 | Hmga2 | 0.922363 |
| piR-mmu-48954623 | Ap1g2 | 0.921732 |
| piR-mmu-48954623 | 2610001J05Rik | 0.91954 |
| piR-mmu-48954623 | Wtap | 0.916353 |
| piR-mmu-48954623 | Trmt10a | 0.913283 |
| piR-mmu-48954623 | Man1a2 | 0.905456 |
| piR-mmu-48954623 | Gm8898 | 0.904572 |
| piR-mmu-48954623 | Hormad2 | 0.901074 |
| piR-mmu-7474221 | 4930474N05Rik | 0.964933 |
| piR-mmu-7474221 | Hs6st2 | 0.956404 |
| piR-mmu-7474221 | Rsf1 | 0.938396 |
| piR-mmu-7474221 | Zfp521 | 0.933961 |
| piR-mmu-7474221 | Slc5a4b | 0.929418 |
| piR-mmu-7474221 | Gm17359 | 0.927758 |
| piR-mmu-7474221 | Rab21 | 0.927355 |
| piR-mmu-7474221 | Ttn | 0.92542 |
| piR-mmu-7474221 | Gm4631 | 0.924672 |
| piR-mmu-7474221 | 1700019D03Rik | 0.918775 |
| piR-mmu-7474221 | Fst | 0.916383 |
| piR-mmu-7474221 | Esyt2 | 0.901094 |

The piRNA target gene prediction was performed with MR-microT (aggregated score ≥ 0.9).

**Supplementary Table S4b: Bioinformatics prediction target genes of oral-specific differentially expressed piRNAs**

| OFFICIAL_GENE_SYMBOL | Name | Species |
|---|---|---|
| Cdon | cell adhesion molecule-related/down-regulated by oncogenes(Cdon) | Mus musculus |
| Etnk1 | ethanolamine kinase 1(Etnk1) | Mus musculus |
| Fam114a2 | family with sequence similarity 114, member A2(Fam114a2) | Mus musculus |
| Zmym5 | zinc finger, MYM-type 5(Zmym5) | Mus musculus |
| Necab1 | N-terminal EF-hand calcium binding protein 1(Necab1) | Mus musculus |
| Nexn | nexilin(Nexn) | Mus musculus |
| Rita1 | RBPJ interacting and tubulin associated 1(Rita1) | Mus musculus |
| Edn1 | endothelin 1(Edn1) | Mus musculus |
| Tph1 | tryptophan hydroxylase 1(Tph1) | Mus musculus |
| Ppp4c | protein phosphatase 4, catalytic subunit(Ppp4c) | Mus musculus |
| Sirt1 | sirtuin 1(Sirt1) | Mus musculus |
| Strap | serine/threonine kinase receptor associated protein(Strap) | Mus musculus |
| Slmap | sarcolemma associated protein(Slmap) | Mus musculus |
| Pnisr | PNN interacting serine/arginine-rich(Pnisr) | Mus musculus |
| Spcs2 | signal peptidase complex subunit 2 homolog (S. cerevisiae)(Spcs2) | Mus musculus |
| Ect2 | ect2 oncogene(Ect2) | Mus musculus |
| Rab30 | RAB30, member RAS oncogene family(Rab30) | Mus musculus |
| Speer3 | spermatogenesis associated glutamate (E)-rich protein 3(Speer3) | Mus musculus |
| Mphosph9 | M-phase phosphoprotein 9(Mphosph9) | Mus musculus |
| Syncrip | synaptotagmin binding, cytoplasmic RNA interacting protein(Syncrip) | Mus musculus |
| Trip4 | thyroid hormone receptor interactor 4(Trip4) | Mus musculus |
| Hnrnpr | heterogeneous nuclear ribonucleoprotein R(Hnrnpr) | Mus musculus |
| Rsf1 | remodeling and spacing factor 1(Rsf1) | Mus musculus |
| Gm4631 | predicted gene 4631(Gm4631) | Mus musculus |
| Ldah | lipid droplet associated hydrolase(Ldah) | Mus musculus |
| Gtpbp8 | GTP-binding protein 8 (putative)(Gtpbp8) | Mus musculus |
| Lce1c | late cornified envelope 1C(Lce1c) | Mus musculus |
| Lce1a2 | late cornified envelope 1A2(Lce1a2) | Mus musculus |
| Whsc1 | Wolf-Hirschhorn syndrome candidate 1 (human)(Whsc1) | Mus musculus |
| AF529169 | cDNA sequence AF529169(AF529169) | Mus musculus |
| Frmpd4 | FERM and PDZ domain containing 4(Frmpd4) | Mus musculus |
| Chchd3 | coiled-coil-helix-coiled-coil-helix domain containing 3(Chchd3) | Mus musculus |
| Anks4b | ankyrin repeat and sterile alpha motif domain containing 4B(Anks4b) | Mus musculus |
| Kif5a | kinesin family member 5A(Kif5a) | Mus musculus |
| Kif5b | kinesin family member 5B(Kif5b) | Mus musculus |
| Ppp1r1c | protein phosphatase 1, regulatory (inhibitor) subunit 1C(Ppp1r1c) | Mus musculus |

| | | |
|---|---|---|
| Ism1 | isthmin 1, angiogenesis inhibitor(Ism1) | Mus musculus |
| Psmd9 | proteasome (prosome, macropain) 26S subunit, non-ATPase, 9(Psmd9) | Mus musculus |
| Mier3 | MIER family member 3(Mier3) | Mus musculus |
| Csmd3 | CUB and Sushi multiple domains 3(Csmd3) | Mus musculus |
| Gria2 | glutamate receptor, ionotropic, AMPA2 (alpha 2)(Gria2) | Mus musculus |
| Dazl | deleted in azoospermia-like(Dazl) | Mus musculus |
| Gm7293 | glyceraldehyde-3-phosphate dehydrogenase pseudogene(Gm7293) | Mus musculus |
| Nfat5 | nuclear factor of activated T cells 5(Nfat5) | Mus musculus |
| Mafg | v-maf musculoaponeurotic fibrosarcoma oncogene family, protein G (avian)(Maf | Mus musculus |
| Reps2 | RALBP1 associated Eps domain containing protein 2(Reps2) | Mus musculus |
| Kcnh7 | potassium voltage-gated channel, subfamily H (eag-related), member 7(Kcnh7) | Mus musculus |
| Zzz3 | zinc finger, ZZ domain containing 3(Zzz3) | Mus musculus |
| Tmem215 | transmembrane protein 215(Tmem215) | Mus musculus |
| Wnt7a | wingless-type MMTV integration site family, member 7A(Wnt7a) | Mus musculus |
| Nek11 | NIMA (never in mitosis gene a)-related expressed kinase 11(Nek11) | Mus musculus |
| Fam134a | family with sequence similarity 134, member A(Fam134a) | Mus musculus |
| Gm8271 | predicted gene 8271(Gm8271) | Mus musculus |
| Zfp740 | zinc finger protein 740(Zfp740) | Mus musculus |
| Lce1a1 | late cornified envelope 1A1(Lce1a1) | Mus musculus |
| Papolg | poly(A) polymerase gamma(Papolg) | Mus musculus |
| Mtmr4 | myotubularin related protein 4(Mtmr4) | Mus musculus |
| Foxj3 | forkhead box J3(Foxj3) | Mus musculus |
| Ppp1cb | protein phosphatase 1, catalytic subunit, beta isoform(Ppp1cb) | Mus musculus |
| Chic2 | cysteine-rich hydrophobic domain 2(Chic2) | Mus musculus |
| 4932438A13Rik | RIKEN cDNA 4932438A13 gene(4932438A13Rik) | Mus musculus |
| Brd1 | bromodomain containing 1(Brd1) | Mus musculus |
| Eif2ak3 | eukaryotic translation initiation factor 2 alpha kinase 3(Eif2ak3) | Mus musculus |
| Zfp810 | zinc finger protein 810(Zfp810) | Mus musculus |
| Anapc11 | anaphase promoting complex subunit 11(Anapc11) | Mus musculus |
| Trmt10a | tRNA methyltransferase 10A(Trmt10a) | Mus musculus |
| Hnrnpd | heterogeneous nuclear ribonucleoprotein D(Hnrnpd) | Mus musculus |
| Tmem132a | transmembrane protein 132A(Tmem132a) | Mus musculus |
| Gm8247 | predicted gene 8247(Gm8247) | Mus musculus |
| Slco1a1 | solute carrier organic anion transporter family, member 1a1(Slco1a1) | Mus musculus |
| Atp1a2 | ATPase, Na+/K+ transporting, alpha 2 polypeptide(Atp1a2) | Mus musculus |
| Rbm15 | RNA binding motif protein 15(Rbm15) | Mus musculus |
| Bbx | bobby sox homolog (Drosophila)(Bbx) | Mus musculus |
| Ctbs | chitobiase, di-N-acetyl-(Ctbs) | Mus musculus |
| 1700019D03Rik | RIKEN cDNA 1700019D03 gene(1700019D03Rik) | Mus musculus |

| | | |
|---|---|---|
| Mettl3 | methyltransferase like 3(Mettl3) | Mus musculus |
| Pcm1 | pericentriolar material 1(Pcm1) | Mus musculus |
| Lrriq4 | leucine-rich repeats and IQ motif containing 4(Lrriq4) | Mus musculus |
| Krt76 | keratin 76(Krt76) | Mus musculus |
| Anapc2 | anaphase promoting complex subunit 2(Anapc2) | Mus musculus |
| Adam9 | a disintegrin and metallopeptidase domain 9 (meltrin gamma)(Adam9) | Mus musculus |
| Lce1f | late cornified envelope 1F(Lce1f) | Mus musculus |
| Ptbp3 | polypyrimidine tract binding protein 3(Ptbp3) | Mus musculus |
| Fgfr2 | fibroblast growth factor receptor 2(Fgfr2) | Mus musculus |
| Rdm1 | RAD52 motif 1(Rdm1) | Mus musculus |
| Slc22a23 | solute carrier family 22, member 23(Slc22a23) | Mus musculus |
| Gfral | GDNF family receptor alpha like(Gfral) | Mus musculus |
| Magt1 | magnesium transporter 1(Magt1) | Mus musculus |
| Pdcd5 | programmed cell death 5(Pdcd5) | Mus musculus |
| Ncaph2 | non-SMC condensin II complex, subunit H2(Ncaph2) | Mus musculus |
| Upf3b | UPF3 regulator of nonsense transcripts homolog B (yeast)(Upf3b) | Mus musculus |
| Nfib | nuclear factor I/B(Nfib) | Mus musculus |
| Nfia | nuclear factor I/A(Nfia) | Mus musculus |
| Zmat1 | zinc finger, matrin type 1(Zmat1) | Mus musculus |
| Clip4 | CAP-GLY domain containing linker protein family, member 4(Clip4) | Mus musculus |
| Mier1 | MEIR1 treanscription regulator(Mier1) | Mus musculus |
| Foxn2 | forkhead box N2(Foxn2) | Mus musculus |
| E2f7 | E2F transcription factor 7(E2f7) | Mus musculus |
| Trmt2b | TRM2 tRNA methyltransferase 2B(Trmt2b) | Mus musculus |
| App | amyloid beta (A4) precursor protein(App) | Mus musculus |
| Gm14744 | predicted gene 14744(Gm14744) | Mus musculus |
| Ppm1g | protein phosphatase 1G (formerly 2C), magnesium-dependent, gamma isoform( | Mus musculus |
| Pcdhb9 | protocadherin beta 9(Pcdhb9) | Mus musculus |
| Inip | INTS3 and NABP interacting protein(Inip) | Mus musculus |
| Relb | avian reticuloendotheliosis viral (v-rel) oncogene related B(Relb) | Mus musculus |
| Fermt2 | fermitin family member 2(Fermt2) | Mus musculus |
| Ago2 | argonaute RISC catalytic subunit 2(Ago2) | Mus musculus |
| Parp6 | poly (ADP-ribose) polymerase family, member 6(Parp6) | Mus musculus |
| Rps6kb1 | ribosomal protein S6 kinase, polypeptide 1(Rps6kb1) | Mus musculus |
| Rgs17 | regulator of G-protein signaling 17(Rgs17) | Mus musculus |
| Phc3 | polyhomeotic-like 3 (Drosophila)(Phc3) | Mus musculus |
| Fmr1 | fragile X mental retardation syndrome 1(Fmr1) | Mus musculus |
| Lce1g | late cornified envelope 1G(Lce1g) | Mus musculus |
| Rcsd1 | RCSD domain containing 1(Rcsd1) | Mus musculus |

| | | |
|---|---|---|
| Spopl | speckle-type POZ protein-like(Spopl) | Mus musculus |
| Psmf1 | proteasome (prosome, macropain) inhibitor subunit 1(Psmf1) | Mus musculus |
| Ythdf2 | YTH domain family 2(Ythdf2) | Mus musculus |
| Dip2c | disco interacting protein 2 homolog C(Dip2c) | Mus musculus |
| Srgn | serglycin(Srgn) | Mus musculus |
| Inpp4a | inositol polyphosphate-4-phosphatase, type I(Inpp4a) | Mus musculus |
| Ropn1 | ropporin, rhophilin associated protein 1(Ropn1) | Mus musculus |
| Lce1b | late cornified envelope 1B(Lce1b) | Mus musculus |
| Plppr4 | phospholipid phosphatase related 4(Plppr4) | Mus musculus |
| Iqch | IQ motif containing H(Iqch) | Mus musculus |
| Ltbp2 | latent transforming growth factor beta binding protein 2(Ltbp2) | Mus musculus |
| 4930452B06Rik | RIKEN cDNA 4930452B06 gene(4930452B06Rik) | Mus musculus |
| 4930474N05Rik | RIKEN cDNA 4930474N05 gene(4930474N05Rik) | Mus musculus |
| Ddx6 | DEAD (Asp-Glu-Ala-Asp) box polypeptide 6(Ddx6) | Mus musculus |
| Ralgps2 | Ral GEF with PH domain and SH3 binding motif 2(Ralgps2) | Mus musculus |
| Gclm | glutamate-cysteine ligase, modifier subunit(Gclm) | Mus musculus |
| Phf3 | PHD finger protein 3(Phf3) | Mus musculus |
| Dagla | diacylglycerol lipase, alpha(Dagla) | Mus musculus |
| Ddx3x | DEAD/H (Asp-Glu-Ala-Asp/His) box polypeptide 3, X-linked(Ddx3x) | Mus musculus |
| Fxn | frataxin(Fxn) | Mus musculus |
| Tgfbr1 | transforming growth factor, beta receptor I(Tgfbr1) | Mus musculus |
| Khdc1a | KH domain containing 1A(Khdc1a) | Mus musculus |
| Ssfa2 | sperm specific antigen 2(Ssfa2) | Mus musculus |
| Hist2h2be | histone cluster 2, H2be(Hist2h2be) | Mus musculus |
| Itga10 | integrin, alpha 10(Itga10) | Mus musculus |
| Wdr43 | WD repeat domain 43(Wdr43) | Mus musculus |
| Trim63 | tripartite motif-containing 63(Trim63) | Mus musculus |
| Cisd1 | CDGSH iron sulfur domain 1(Cisd1) | Mus musculus |
| Zbtb20 | zinc finger and BTB domain containing 20(Zbtb20) | Mus musculus |
| Esyt2 | extended synaptotagmin-like protein 2(Esyt2) | Mus musculus |
| Ube2v2 | ubiquitin-conjugating enzyme E2 variant 2(Ube2v2) | Mus musculus |
| Megf11 | multiple EGF-like-domains 11(Megf11) | Mus musculus |
| Foxk1 | forkhead box K1(Foxk1) | Mus musculus |
| Fosl2 | fos-like antigen 2(Fosl2) | Mus musculus |
| Ivns1abp | influenza virus NS1A binding protein(Ivns1abp) | Mus musculus |
| Ankrd24 | ankyrin repeat domain 24(Ankrd24) | Mus musculus |
| Enah | enabled homolog (Drosophila)(Enah) | Mus musculus |
| Ppp4r2 | protein phosphatase 4, regulatory subunit 2(Ppp4r2) | Mus musculus |
| Ahdc1 | AT hook, DNA binding motif, containing 1(Ahdc1) | Mus musculus |

| | | |
|---|---|---|
| Chrnb4 | cholinergic receptor, nicotinic, beta polypeptide 4(Chrnb4) | Mus musculus |
| Rab11a | RAB11A, member RAS oncogene family(Rab11a) | Mus musculus |
| Ube2k | ubiquitin-conjugating enzyme E2K(Ube2k) | Mus musculus |
| Ephb1 | Eph receptor B1(Ephb1) | Mus musculus |
| Celf4 | CUGBP, Elav-like family member 4(Celf4) | Mus musculus |
| E130114P18Rik | RIKEN cDNA E130114P18 gene(E130114P18Rik) | Mus musculus |
| Tmem251 | transmembrane protein 251(Tmem251) | Mus musculus |
| Plrg1 | pleiotropic regulator 1(Plrg1) | Mus musculus |
| Fam122b | family with sequence similarity 122, member B(Fam122b) | Mus musculus |
| Xpo1 | exportin 1(Xpo1) | Mus musculus |
| 1700010B08Rik | RIKEN cDNA 1700010B08 gene(1700010B08Rik) | Mus musculus |
| Hormad2 | HORMA domain containing 2(Hormad2) | Mus musculus |
| D830030K20Rik | RIKEN cDNA D830030K20 gene(D830030K20Rik) | Mus musculus |
| Dpp10 | dipeptidylpeptidase 10(Dpp10) | Mus musculus |
| Zfand3 | zinc finger, AN1-type domain 3(Zfand3) | Mus musculus |
| Glis3 | GLIS family zinc finger 3(Glis3) | Mus musculus |
| Fst | follistatin(Fst) | Mus musculus |
| Kcnh2 | potassium voltage-gated channel, subfamily H (eag-related), member 2(Kcnh2) | Mus musculus |
| Sympk | symplekin(Sympk) | Mus musculus |
| Kdm7a | lysine (K)-specific demethylase 7A(Kdm7a) | Mus musculus |
| Acadl | acyl-Coenzyme A dehydrogenase, long-chain(Acadl) | Mus musculus |
| Fsbp | fibrinogen silencer binding protein(Fsbp) | Mus musculus |
| Lats1 | large tumor suppressor(Lats1) | Mus musculus |
| Cdc73 | cell division cycle 73, Paf1/RNA polymerase II complex component(Cdc73) | Mus musculus |
| Nsa2 | NSA2 ribosome biogenesis homolog(Nsa2) | Mus musculus |
| Mysm1 | myb-like, SWIRM and MPN domains 1(Mysm1) | Mus musculus |
| F830016B08Rik | RIKEN cDNA F830016B08 gene(F830016B08Rik) | Mus musculus |
| BC052040 | cDNA sequence BC052040(BC052040) | Mus musculus |
| Ctdspl2 | CTD (carboxy-terminal domain, RNA polymerase II, polypeptide A) small phospha | Mus musculus |
| Bend6 | BEN domain containing 6(Bend6) | Mus musculus |
| Runx1t1 | runt-related transcription factor 1; translocated to, 1 (cyclin D-related)(Runx1t1) | Mus musculus |
| Phip | pleckstrin homology domain interacting protein(Phip) | Mus musculus |
| Prex2 | phosphatidylinositol-3,4,5-trisphosphate-dependent Rac exchange factor 2(Prex | Mus musculus |
| Myh8 | myosin, heavy polypeptide 8, skeletal muscle, perinatal(Myh8) | Mus musculus |
| Mxi1 | MAX interactor 1, dimerization protein(Mxi1) | Mus musculus |
| Kank4 | KN motif and ankyrin repeat domains 4(Kank4) | Mus musculus |
| Ptpn12 | protein tyrosine phosphatase, non-receptor type 12(Ptpn12) | Mus musculus |
| Zbtb4 | zinc finger and BTB domain containing 4(Zbtb4) | Mus musculus |
| Cbfb | core binding factor beta(Cbfb) | Mus musculus |

| | | |
|---|---|---|
| Sumo2 | small ubiquitin-like modifier 2(Sumo2) | Mus musculus |
| Zeb2 | zinc finger E-box binding homeobox 2(Zeb2) | Mus musculus |
| Slc25a46 | solute carrier family 25, member 46(Slc25a46) | Mus musculus |
| Cbl | Casitas B-lineage lymphoma(Cbl) | Mus musculus |
| Cadm2 | cell adhesion molecule 2(Cadm2) | Mus musculus |
| Igf1r | insulin-like growth factor I receptor(Igf1r) | Mus musculus |
| Sdc2 | syndecan 2(Sdc2) | Mus musculus |
| Son | Son DNA binding protein(Son) | Mus musculus |
| Zfand1 | zinc finger, AN1-type domain 1(Zfand1) | Mus musculus |
| Fip1l1 | FIP1 like 1 (S. cerevisiae)(Fip1l1) | Mus musculus |
| Ptprb | protein tyrosine phosphatase, receptor type, B(Ptprb) | Mus musculus |
| Atp2b1 | ATPase, Ca++ transporting, plasma membrane 1(Atp2b1) | Mus musculus |
| Tmem50a | transmembrane protein 50A(Tmem50a) | Mus musculus |
| Gm3278 | predicted gene 3278(Gm3278) | Mus musculus |
| Rlim | ring finger protein, LIM domain interacting(Rlim) | Mus musculus |
| Cep350 | centrosomal protein 350(Cep350) | Mus musculus |
| Dctn2 | dynactin 2(Dctn2) | Mus musculus |
| Lcp2 | lymphocyte cytosolic protein 2(Lcp2) | Mus musculus |
| Plcz1 | phospholipase C, zeta 1(Plcz1) | Mus musculus |
| Ctu1 | cytosolic thiouridylase subunit 1(Ctu1) | Mus musculus |
| Zfp141 | zinc finger protein 141(Zfp141) | Mus musculus |
| Vkorc1l1 | vitamin K epoxide reductase complex, subunit 1-like 1(Vkorc1l1) | Mus musculus |
| Meioc | meiosis specific with coiled-coil domain(Meioc) | Mus musculus |
| Rfesd | Rieske (Fe-S) domain containing(Rfesd) | Mus musculus |
| Prdm6 | PR domain containing 6(Prdm6) | Mus musculus |
| Tbc1d12 | TBC1D12: TBC1 domain family, member 12(Tbc1d12) | Mus musculus |
| Smg1 | SMG1 homolog, phosphatidylinositol 3-kinase-related kinase (C. elegans)(Smg1) | Mus musculus |
| 1700066M21Rik | RIKEN cDNA 1700066M21 gene(1700066M21Rik) | Mus musculus |
| Ildr2 | immunoglobulin-like domain containing receptor 2(Ildr2) | Mus musculus |
| Cast | calpastatin(Cast) | Mus musculus |
| Iffo2 | intermediate filament family orphan 2(Iffo2) | Mus musculus |
| Rabep1 | rabaptin, RAB GTPase binding effector protein 1(Rabep1) | Mus musculus |
| Pomt2 | protein-O-mannosyltransferase 2(Pomt2) | Mus musculus |
| Gch1 | GTP cyclohydrolase 1(Gch1) | Mus musculus |
| Abcd4 | ATP-binding cassette, sub-family D (ALD), member 4(Abcd4) | Mus musculus |
| Serpini1 | serine (or cysteine) peptidase inhibitor, clade I, member 1(Serpini1) | Mus musculus |
| Fam149b | family with sequence similarity 149, member B(Fam149b) | Mus musculus |
| Tead1 | TEA domain family member 1(Tead1) | Mus musculus |
| Ogt | O-linked N-acetylglucosamine (GlcNAc) transferase (UDP-N-acetylglucosamine:p | Mus musculus |

| | | |
|---|---|---|
| Eme1 | essential meiotic structure-specific endonuclease 1(Eme1) | Mus musculus |
| Gm3532 | predicted gene 3532(Gm3532) | Mus musculus |
| Mmp13 | matrix metallopeptidase 13(Mmp13) | Mus musculus |
| Mrpl33 | mitochondrial ribosomal protein L33(Mrpl33) | Mus musculus |
| Plagl1 | pleiomorphic adenoma gene-like 1(Plagl1) | Mus musculus |
| Ccnc | cyclin C(Ccnc) | Mus musculus |
| Setd5 | SET domain containing 5(Setd5) | Mus musculus |
| Cacna1b | calcium channel, voltage-dependent, N type, alpha 1B subunit(Cacna1b) | Mus musculus |
| Gm17359 | predicted gene, 17359(Gm17359) | Mus musculus |
| Esco1 | establishment of sister chromatid cohesion N-acetyltransferase 1(Esco1) | Mus musculus |
| Ulbp1 | UL16 binding protein 1(Ulbp1) | Mus musculus |
| Ptprd | protein tyrosine phosphatase, receptor type, D(Ptprd) | Mus musculus |
| Rab21 | RAB21, member RAS oncogene family(Rab21) | Mus musculus |
| Wtap | Wilms tumour 1-associating protein(Wtap) | Mus musculus |
| Btg3 | B cell translocation gene 3(Btg3) | Mus musculus |
| Mars2 | methionine-tRNA synthetase 2 (mitochondrial)(Mars2) | Mus musculus |
| Elavl1 | ELAV (embryonic lethal, abnormal vision)-like 1 (Hu antigen R)(Elavl1) | Mus musculus |
| Kctd8 | potassium channel tetramerisation domain containing 8(Kctd8) | Mus musculus |
| Tnrc6b | trinucleotide repeat containing 6b(Tnrc6b) | Mus musculus |
| Nol4l | nucleolar protein 4-like(Nol4l) | Mus musculus |
| Unk | unkempt family zinc finger(Unk) | Mus musculus |
| Ap1g2 | adaptor protein complex AP-1, gamma 2 subunit(Ap1g2) | Mus musculus |
| Prkag2 | protein kinase, AMP-activated, gamma 2 non-catalytic subunit(Prkag2) | Mus musculus |
| Cfap100 | cilia and flagella associated protein 100(Cfap100) | Mus musculus |
| Zfp160 | zinc finger protein 160(Zfp160) | Mus musculus |
| Ppp2r5e | protein phosphatase 2, regulatory subunit B', epsilon(Ppp2r5e) | Mus musculus |
| Dmd | dystrophin, muscular dystrophy(Dmd) | Mus musculus |
| Ppp2r5c | protein phosphatase 2, regulatory subunit B', gamma(Ppp2r5c) | Mus musculus |
| Med13 | mediator complex subunit 13(Med13) | Mus musculus |
| Abcc5 | ATP-binding cassette, sub-family C (CFTR/MRP), member 5(Abcc5) | Mus musculus |
| Gm15140 | predicted gene 15140(Gm15140) | Mus musculus |
| Pura | purine rich element binding protein A(Pura) | Mus musculus |
| Prpf40a | pre-mRNA processing factor 40A(Prpf40a) | Mus musculus |
| Gmeb1 | glucocorticoid modulatory element binding protein 1(Gmeb1) | Mus musculus |
| St8sia4 | ST8 alpha-N-acetyl-neuraminide alpha-2,8-sialyltransferase 4(St8sia4) | Mus musculus |
| Adamts5 | a disintegrin-like and metallopeptidase (reprolysin type) with thrombospondin t | Mus musculus |
| Zfyve26 | zinc finger, FYVE domain containing 26(Zfyve26) | Mus musculus |
| Slc5a4b | solute carrier family 5 (neutral amino acid transporters, system A), member 4b(S | Mus musculus |
| Hs6st2 | heparan sulfate 6-O-sulfotransferase 2(Hs6st2) | Mus musculus |

| | | |
|---|---|---|
| Gm19965 | zinc finger protein 234-like(Gm19965) | Mus musculus |
| C77370 | expressed sequence C77370(C77370) | Mus musculus |
| Rag2 | recombination activating gene 2(Rag2) | Mus musculus |
| Arhgap11a | Rho GTPase activating protein 11A(Arhgap11a) | Mus musculus |
| Spred1 | sprouty protein with EVH-1 domain 1, related sequence(Spred1) | Mus musculus |
| Ccdc120 | coiled-coil domain containing 120(Ccdc120) | Mus musculus |
| Amfr | autocrine motility factor receptor(Amfr) | Mus musculus |
| Nckap1l | NCK associated protein 1 like(Nckap1l) | Mus musculus |
| Cdkl5 | cyclin-dependent kinase-like 5(Cdkl5) | Mus musculus |
| Bdnf | brain derived neurotrophic factor(Bdnf) | Mus musculus |
| Mgat4c | MGAT4 family, member C(Mgat4c) | Mus musculus |
| Rpgrip1l | Rpgrip1-like(Rpgrip1l) | Mus musculus |
| Acin1 | apoptotic chromatin condensation inducer 1(Acin1) | Mus musculus |
| Rc3h1 | RING CCCH (C3H) domains 1(Rc3h1) | Mus musculus |
| Rnf115 | ring finger protein 115(Rnf115) | Mus musculus |
| Gm4924 | predicted gene 4924(Gm4924) | Mus musculus |
| Fblim1 | filamin binding LIM protein 1(Fblim1) | Mus musculus |
| Cpeb4 | cytoplasmic polyadenylation element binding protein 4(Cpeb4) | Mus musculus |
| Thoc1 | THO complex 1(Thoc1) | Mus musculus |
| Myocd | myocardin(Myocd) | Mus musculus |
| Ddx55 | DEAD (Asp-Glu-Ala-Asp) box polypeptide 55(Ddx55) | Mus musculus |
| Fubp1 | far upstream element (FUSE) binding protein 1(Fubp1) | Mus musculus |
| Camk1d | calcium/calmodulin-dependent protein kinase ID(Camk1d) | Mus musculus |
| Mib1 | mindbomb E3 ubiquitin protein ligase 1(Mib1) | Mus musculus |
| Kpna4 | karyopherin (importin) alpha 4(Kpna4) | Mus musculus |
| Ccdc163 | coiled-coil domain containing 163(Ccdc163) | Mus musculus |
| Wasf2 | WAS protein family, member 2(Wasf2) | Mus musculus |
| Zfp553 | zinc finger protein 553(Zfp553) | Mus musculus |
| Tprkb | Tp53rk binding protein(Tprkb) | Mus musculus |
| Dopey1 | dopey family member 1(Dopey1) | Mus musculus |
| Mbd3l2 | methyl-CpG binding domain protein 3-like 2(Mbd3l2) | Mus musculus |
| Zak | sterile alpha motif and leucine zipper containing kinase AZK(Zak) | Mus musculus |
| Fam120a | family with sequence similarity 120, member A(Fam120a) | Mus musculus |
| Zfp521 | zinc finger protein 521(Zfp521) | Mus musculus |
| Onecut1 | one cut domain, family member 1(Onecut1) | Mus musculus |
| Entpd3 | ectonucleoside triphosphate diphosphohydrolase 3(Entpd3) | Mus musculus |
| Supt16 | suppressor of Ty 16(Supt16) | Mus musculus |
| Zfp704 | zinc finger protein 704(Zfp704) | Mus musculus |
| Trpm8 | transient receptor potential cation channel, subfamily M, member 8(Trpm8) | Mus musculus |

| | | |
|---|---|---|
| Agps | alkylglycerone phosphate synthase(Agps) | Mus musculus |
| Tanc2 | tetratricopeptide repeat, ankyrin repeat and coiled-coil containing 2(Tanc2) | Mus musculus |
| Ttn | titin(Ttn) | Mus musculus |
| Esrrg | estrogen-related receptor gamma(Esrrg) | Mus musculus |
| Chd9 | chromodomain helicase DNA binding protein 9(Chd9) | Mus musculus |
| Jph1 | junctophilin 1(Jph1) | Mus musculus |
| Gabrb2 | gamma-aminobutyric acid (GABA) A receptor, subunit beta 2(Gabrb2) | Mus musculus |
| Man1a2 | mannosidase, alpha, class 1A, member 2(Man1a2) | Mus musculus |
| Fus | fused in sarcoma(Fus) | Mus musculus |
| Bcl9 | B cell CLL/lymphoma 9(Bcl9) | Mus musculus |
| Gm10282 | high mobility group nucleosomal binding domain 2 pseudogene(Gm10282) | Mus musculus |
| Arhgef3 | Rho guanine nucleotide exchange factor (GEF) 3(Arhgef3) | Mus musculus |
| Mtf2 | metal response element binding transcription factor 2(Mtf2) | Mus musculus |
| Id4 | inhibitor of DNA binding 4(Id4) | Mus musculus |
| Hmga2 | high mobility group AT-hook 2(Hmga2) | Mus musculus |
| Crisp3 | cysteine-rich secretory protein 3(Crisp3) | Mus musculus |
| Ccdc150 | coiled-coil domain containing 150(Ccdc150) | Mus musculus |
| Meis1 | Meis homeobox 1(Meis1) | Mus musculus |
| C1galt1 | core 1 synthase, glycoprotein-N-acetylgalactosamine 3-beta-galactosyltransferas | Mus musculus |
| Fam199x | family with sequence similarity 199, X-linked(Fam199x) | Mus musculus |
| Trim36 | tripartite motif-containing 36(Trim36) | Mus musculus |
| Gm5415 | predicted gene 5415(Gm5415) | Mus musculus |
| Kdm5c | lysine (K)-specific demethylase 5C(Kdm5c) | Mus musculus |
| Zfp800 | zinc finger protein 800(Zfp800) | Mus musculus |
| Gria3 | glutamate receptor, ionotropic, AMPA3 (alpha 3)(Gria3) | Mus musculus |
| Ptcd1 | pentatricopeptide repeat domain 1(Ptcd1) | Mus musculus |
| Gspt1 | G1 to S phase transition 1(Gspt1) | Mus musculus |
| Dcstamp | dendrocyte expressed seven transmembrane protein(Dcstamp) | Mus musculus |
| Crebrf | CREB3 regulatory factor(Crebrf) | Mus musculus |
| Taok1 | TAO kinase 1(Taok1) | Mus musculus |
| Skap1 | src family associated phosphoprotein 1(Skap1) | Mus musculus |
| Hrh2 | histamine receptor H2(Hrh2) | Mus musculus |
| Ak6 | adenylate kinase 6(Ak6) | Mus musculus |
| Ccdc125 | coiled-coil domain containing 125(Ccdc125) | Mus musculus |
| Mef2d | myocyte enhancer factor 2D(Mef2d) | Mus musculus |
| Oraov1 | oral cancer overexpressed 1(Oraov1) | Mus musculus |
| Mecp2 | methyl CpG binding protein 2(Mecp2) | Mus musculus |
| Rnf6 | ring finger protein (C3H2C3 type) 6(Rnf6) | Mus musculus |
| 5430402E10Rik | RIKEN cDNA 5430402E10 gene(5430402E10Rik) | Mus musculus |

| | | |
|---|---|---|
| Nrep | neuronal regeneration related protein(Nrep) | Mus musculus |
| Susd6 | sushi domain containing 6(Susd6) | Mus musculus |
| Snx7 | sorting nexin 7(Snx7) | Mus musculus |
| Tmem151a | transmembrane protein 151A(Tmem151a) | Mus musculus |
| Sbf1 | SET binding factor 1(Sbf1) | Mus musculus |
| Abcg8 | ATP-binding cassette, sub-family G (WHITE), member 8(Abcg8) | Mus musculus |
| Dennd5b | DENN/MADD domain containing 5B(Dennd5b) | Mus musculus |
| Cd46 | CD46 antigen, complement regulatory protein(Cd46) | Mus musculus |
| 2610001J05Rik | RIKEN cDNA 2610001J05 gene(2610001J05Rik) | Mus musculus |
| Rnf217 | ring finger protein 217(Rnf217) | Mus musculus |
| Lmbrd1 | LMBR1 domain containing 1(Lmbrd1) | Mus musculus |
| D1Ertd622e | DNA segment, Chr 1, ERATO Doi 622, expressed(D1Ertd622e) | Mus musculus |
| Umad1 | UMAP1-MVP12 associated (UMA) domain containing 1(Umad1) | Mus musculus |
| Dhx40 | DEAH (Asp-Glu-Ala-His) box polypeptide 40(Dhx40) | Mus musculus |
| Fbxo28 | F-box protein 28(Fbxo28) | Mus musculus |
| Srsf3 | serine/arginine-rich splicing factor 3(Srsf3) | Mus musculus |
| March6 | Membrane Associated Ring-CH-Type Finger 9 (March6) | Mus musculus |
| Unmapped Id | Gm8898 | |
| Unmapped Id | Gm20537 | |

Mapped genes were used for pathway analysis.

**Supplementary Table S5a: Bioinformatics prediction piRNA-target gene pairs of skin-specific differentially expressed piRNAs**

| piRNA | target gene | MR-microT score |
|---|---|---|
| piR-mmu-49079980 | Slc5a4b | 0.971082 |
| piR-mmu-49079980 | Esyt2 | 0.969098 |
| piR-mmu-49079980 | 4930474N05Rik | 0.965645 |
| piR-mmu-49079980 | Hs6st2 | 0.960036 |
| piR-mmu-49079980 | Zfp521 | 0.935732 |
| piR-mmu-49079980 | Rsf1 | 0.934158 |
| piR-mmu-49079980 | Rab21 | 0.930514 |
| piR-mmu-49079980 | Gm4631 | 0.927044 |
| piR-mmu-49079980 | Fst | 0.922388 |
| piR-mmu-49079980 | Ttn | 0.911675 |
| piR-mmu-49079980 | Cmah | 0.909939 |
| piR-mmu-49251782 | Slc10a7 | 0.999828 |
| piR-mmu-49251782 | Tmsb4x | 0.998987 |
| piR-mmu-49251782 | Msantd2 | 0.998726 |
| piR-mmu-49251782 | Pcsk5 | 0.998053 |
| piR-mmu-49251782 | Ets1 | 0.989109 |
| piR-mmu-49251782 | Cabyr | 0.98892 |
| piR-mmu-49251782 | Snx14 | 0.982791 |
| piR-mmu-49251782 | Cdkl5 | 0.972971 |
| piR-mmu-49251782 | Gpbp1 | 0.972788 |
| piR-mmu-49251782 | Tshz1 | 0.965739 |
| piR-mmu-49251782 | Jarid2 | 0.961579 |
| piR-mmu-49251782 | Fgf14 | 0.956232 |
| piR-mmu-49251782 | Hnrnpa3 | 0.952339 |
| piR-mmu-49251782 | Bdnf | 0.947977 |
| piR-mmu-49251782 | Zfp964 | 0.946726 |
| piR-mmu-49251782 | Ccdc166 | 0.945772 |
| piR-mmu-49251782 | Adprm | 0.943203 |
| piR-mmu-49251782 | Pcdhb14 | 0.942583 |
| piR-mmu-49251782 | Plppr4 | 0.942327 |
| piR-mmu-49251782 | Fbxo33 | 0.938642 |
| piR-mmu-49251782 | Gm3371 | 0.93843 |
| piR-mmu-49251782 | Pik3r1 | 0.937646 |
| piR-mmu-49251782 | Ywhaz | 0.936198 |
| piR-mmu-49251782 | Wipf2 | 0.935729 |
| piR-mmu-49251782 | Rgs2 | 0.935421 |
| piR-mmu-49251782 | St6galnac5 | 0.934992 |
| piR-mmu-49251782 | Pigx | 0.930007 |
| piR-mmu-49251782 | Kctd13 | 0.929246 |
| piR-mmu-49251782 | Zbtb39 | 0.92902 |
| piR-mmu-49251782 | Slc25a53 | 0.928153 |
| piR-mmu-49251782 | Abhd2 | 0.928051 |
| piR-mmu-49251782 | Gm2832 | 0.927476 |
| piR-mmu-49251782 | Nsdhl | 0.926109 |
| piR-mmu-49251782 | Gm5798 | 0.925213 |
| piR-mmu-49251782 | Cand1 | 0.924763 |
| piR-mmu-49251782 | Srsf1 | 0.922216 |
| piR-mmu-49251782 | Wwc2 | 0.918671 |
| piR-mmu-49251782 | Ss18 | 0.918643 |

| | | |
|---|---|---|
| piR-mmu-49251782 | Gm8011 | 0.917939 |
| piR-mmu-49251782 | Map3k1 | 0.917475 |
| piR-mmu-49251782 | Trim2 | 0.915858 |
| piR-mmu-49251782 | Zfp160 | 0.913734 |
| piR-mmu-49251782 | Hmcn1 | 0.913458 |
| piR-mmu-49251782 | Glyctk | 0.913196 |
| piR-mmu-49251782 | Cnksr3 | 0.912792 |
| piR-mmu-49251782 | Pianp | 0.911204 |
| piR-mmu-49251782 | Frs2 | 0.910488 |
| piR-mmu-49251782 | Ptpn14 | 0.908596 |
| piR-mmu-49251782 | Adar | 0.907511 |
| piR-mmu-49251782 | Gm7233 | 0.90725 |
| piR-mmu-49251782 | Atf2 | 0.906255 |
| piR-mmu-49251782 | Sri | 0.905209 |
| piR-mmu-49251782 | Rfesd | 0.900928 |
| piR-mmu-30053093 | Meioc | 0.991041 |
| piR-mmu-30053093 | Ldb2 | 0.988517 |
| piR-mmu-30053093 | Galnt3 | 0.97797 |
| piR-mmu-30053093 | Fign | 0.958555 |
| piR-mmu-30053093 | Eif4g2 | 0.956022 |
| piR-mmu-30053093 | Nfib | 0.954769 |
| piR-mmu-30053093 | Dopey1 | 0.950393 |
| piR-mmu-30053093 | Zbtb20 | 0.948826 |
| piR-mmu-30053093 | Onecut2 | 0.946709 |
| piR-mmu-30053093 | Epb42 | 0.936723 |
| piR-mmu-30053093 | Zfp644 | 0.936206 |
| piR-mmu-30053093 | Pak2 | 0.935901 |
| piR-mmu-30053093 | Rsf1 | 0.931239 |
| piR-mmu-30053093 | Stpg2 | 0.927312 |
| piR-mmu-30053093 | Tspan5 | 0.925403 |
| piR-mmu-30053093 | Fbxo41 | 0.920682 |
| piR-mmu-30053093 | Col5a2 | 0.920069 |
| piR-mmu-30053093 | Enah | 0.918931 |
| piR-mmu-30053093 | Wac | 0.914051 |
| piR-mmu-30053093 | Purb | 0.91297 |
| piR-mmu-30053093 | Matr3 | 0.911603 |
| piR-mmu-30053093 | Ntrk3 | 0.908445 |
| piR-mmu-30053093 | Zmpste24 | 0.906514 |
| piR-mmu-30053093 | Uty | 0.905462 |
| piR-mmu-30053093 | Zyg11b | 0.900537 |
| piR-mmu-30876808 | Meioc | 0.990879 |
| piR-mmu-30876808 | Ldb2 | 0.979519 |
| piR-mmu-30876808 | Galnt3 | 0.977886 |
| piR-mmu-30876808 | Fign | 0.963889 |
| piR-mmu-30876808 | Nfib | 0.956467 |
| piR-mmu-30876808 | Dopey1 | 0.948686 |
| piR-mmu-30876808 | Onecut2 | 0.947444 |
| piR-mmu-30876808 | Zbtb20 | 0.944991 |
| piR-mmu-30876808 | Stpg2 | 0.933436 |
| piR-mmu-30876808 | Zfp644 | 0.928619 |
| piR-mmu-30876808 | Cd47 | 0.92086 |
| piR-mmu-30876808 | Pak2 | 0.920776 |
| piR-mmu-30876808 | Col5a2 | 0.920453 |

| | | |
|---|---|---|
| piR-mmu-30876808 | Fbxo41 | 0.919709 |
| piR-mmu-30876808 | Tspan5 | 0.919518 |
| piR-mmu-30876808 | Wac | 0.912581 |
| piR-mmu-30876808 | Purb | 0.912072 |
| piR-mmu-30876808 | Matr3 | 0.907525 |
| piR-mmu-30876808 | Nfat5 | 0.907259 |
| piR-mmu-30876808 | Usp9x | 0.907167 |
| piR-mmu-30876808 | Ttn | 0.90328 |
| piR-mmu-30876808 | Uty | 0.901149 |
| piR-mmu-30876808 | Zyg11b | 0.900448 |
| piR-mmu-30876808 | Plch1 | 0.900024 |
| piR-mmu-11461049 | 4930474N05Rik | 0.964529 |
| piR-mmu-11461049 | Hs6st2 | 0.956585 |
| piR-mmu-11461049 | Rsf1 | 0.935302 |
| piR-mmu-11461049 | Zfp521 | 0.932021 |
| piR-mmu-11461049 | Rab21 | 0.928738 |
| piR-mmu-11461049 | Esyt2 | 0.928691 |
| piR-mmu-11461049 | Gm4631 | 0.924599 |
| piR-mmu-11461049 | Ttn | 0.921338 |
| piR-mmu-11461049 | Fst | 0.921159 |
| piR-mmu-11461049 | Slc5a4b | 0.902128 |
| piR-mmu-49197585 | Slc5a4b | 0.973539 |
| piR-mmu-49197585 | Esyt2 | 0.969098 |
| piR-mmu-49197585 | 4930474N05Rik | 0.965645 |
| piR-mmu-49197585 | Hs6st2 | 0.95989 |
| piR-mmu-49197585 | Zfp521 | 0.935732 |
| piR-mmu-49197585 | Rsf1 | 0.934158 |
| piR-mmu-49197585 | Rab21 | 0.930514 |
| piR-mmu-49197585 | Gm4631 | 0.927025 |
| piR-mmu-49197585 | Fst | 0.922388 |
| piR-mmu-49197585 | Cmah | 0.920229 |
| piR-mmu-49197585 | Ttn | 0.910353 |
| piR-mmu-23866657 | Meioc | 0.991336 |
| piR-mmu-23866657 | Ldb2 | 0.988502 |
| piR-mmu-23866657 | Galnt3 | 0.978195 |
| piR-mmu-23866657 | Fign | 0.958411 |
| piR-mmu-23866657 | Nfib | 0.955338 |
| piR-mmu-23866657 | Dopey1 | 0.950272 |
| piR-mmu-23866657 | Zbtb20 | 0.949818 |
| piR-mmu-23866657 | Onecut2 | 0.946723 |
| piR-mmu-23866657 | Pak2 | 0.943341 |
| piR-mmu-23866657 | Epb42 | 0.937412 |
| piR-mmu-23866657 | Zfp644 | 0.936582 |
| piR-mmu-23866657 | Tspan5 | 0.933662 |
| piR-mmu-23866657 | Rsf1 | 0.931238 |
| piR-mmu-23866657 | Stpg2 | 0.927275 |
| piR-mmu-23866657 | Fbxo41 | 0.922652 |
| piR-mmu-23866657 | Enah | 0.921516 |
| piR-mmu-23866657 | Col5a2 | 0.919856 |
| piR-mmu-23866657 | Wac | 0.915322 |
| piR-mmu-23866657 | Purb | 0.913821 |
| piR-mmu-23866657 | Matr3 | 0.911134 |
| piR-mmu-23866657 | Ntrk3 | 0.908672 |

| | | |
|---|---|---|
| piR-mmu-23866657 | Uty | 0.908287 |
| piR-mmu-23866657 | Zmpste24 | 0.907485 |
| piR-mmu-23866657 | Cd47 | 0.905447 |
| piR-mmu-23866657 | Spopl | 0.902738 |
| piR-mmu-23866657 | Acan | 0.902035 |
| piR-mmu-23866657 | Ccdc50 | 0.901781 |
| piR-mmu-23866657 | Zyg11b | 0.901142 |
| piR-mmu-23866657 | Ttn | 0.900843 |
| piR-mmu-48824062 | Nrn1 | 0.988051 |
| piR-mmu-48824062 | Zbtb34 | 0.976681 |
| piR-mmu-48824062 | Cbfb | 0.926076 |
| piR-mmu-48824062 | Enho | 0.917577 |
| piR-mmu-48824062 | Flt1 | 0.910782 |
| piR-mmu-48824062 | Eif4b | 0.906451 |
| piR-mmu-27200780 | Xlr5c | 1 |
| piR-mmu-27200780 | Xlr5b | 1 |
| piR-mmu-27200780 | Xlr5a | 0.999998 |
| piR-mmu-27200780 | Gm9936 | 0.99997 |
| piR-mmu-27200780 | Celf2 | 0.999937 |
| piR-mmu-27200780 | Ppp4c | 0.999857 |
| piR-mmu-27200780 | Lmx1a | 0.998007 |
| piR-mmu-27200780 | Unc5c | 0.998003 |
| piR-mmu-27200780 | Kbtbd2 | 0.997098 |
| piR-mmu-27200780 | Gstk1 | 0.996951 |
| piR-mmu-27200780 | Pcdh20 | 0.995121 |
| piR-mmu-27200780 | Vstm2b | 0.994766 |
| piR-mmu-27200780 | Zdhhc15 | 0.99464 |
| piR-mmu-27200780 | Adgre4 | 0.994337 |
| piR-mmu-27200780 | Tmem67 | 0.994264 |
| piR-mmu-27200780 | Ddx3y | 0.993682 |
| piR-mmu-27200780 | Trub2 | 0.991777 |
| piR-mmu-27200780 | Sh3pxd2a | 0.991087 |
| piR-mmu-27200780 | Ccbe1 | 0.990706 |
| piR-mmu-27200780 | Tomm70a | 0.990522 |
| piR-mmu-27200780 | Pdzd4 | 0.989907 |
| piR-mmu-27200780 | Dusp12 | 0.989899 |
| piR-mmu-27200780 | Dmtf1 | 0.989836 |
| piR-mmu-27200780 | Trmt12 | 0.989688 |
| piR-mmu-27200780 | Tmprss12 | 0.989408 |
| piR-mmu-27200780 | Ywhab | 0.988875 |
| piR-mmu-27200780 | Srek1 | 0.984927 |
| piR-mmu-27200780 | Inpp5j | 0.9848 |
| piR-mmu-27200780 | Gm8882 | 0.984429 |
| piR-mmu-27200780 | Vegfb | 0.983391 |
| piR-mmu-27200780 | Pak3 | 0.9824 |
| piR-mmu-27200780 | Fam134c | 0.981919 |
| piR-mmu-27200780 | Hectd2 | 0.98032 |
| piR-mmu-27200780 | Zbtb20 | 0.980134 |
| piR-mmu-27200780 | Wdr38 | 0.97856 |
| piR-mmu-27200780 | Ddx5 | 0.978321 |
| piR-mmu-27200780 | Boc | 0.976826 |
| piR-mmu-27200780 | P2rx1 | 0.976568 |
| piR-mmu-27200780 | Wnt7a | 0.976249 |

| | | |
|---|---|---|
| piR-mmu-27200780 | Clcn1 | 0.975823 |
| piR-mmu-27200780 | Fbxl21 | 0.975379 |
| piR-mmu-27200780 | Btbd1 | 0.973766 |
| piR-mmu-27200780 | Ackr2 | 0.973727 |
| piR-mmu-27200780 | Rsl1d1 | 0.973694 |
| piR-mmu-27200780 | Usp7 | 0.972716 |
| piR-mmu-27200780 | Senp1 | 0.971833 |
| piR-mmu-27200780 | Pak1 | 0.970633 |
| piR-mmu-27200780 | Ip6k2 | 0.970524 |
| piR-mmu-27200780 | Mtf1 | 0.970005 |
| piR-mmu-27200780 | Chek1 | 0.969183 |
| piR-mmu-27200780 | D1Ertd622e | 0.967916 |
| piR-mmu-27200780 | Obox2 | 0.967621 |
| piR-mmu-27200780 | Nhlrc3 | 0.966994 |
| piR-mmu-27200780 | Tifab | 0.966351 |
| piR-mmu-27200780 | Zfp9 | 0.965596 |
| piR-mmu-27200780 | Pde6d | 0.965483 |
| piR-mmu-27200780 | Lin9 | 0.964552 |
| piR-mmu-27200780 | Prdm9 | 0.964152 |
| piR-mmu-27200780 | Ddhd2 | 0.96348 |
| piR-mmu-27200780 | Armc1 | 0.962953 |
| piR-mmu-27200780 | Anxa7 | 0.960673 |
| piR-mmu-27200780 | Fam214b | 0.960189 |
| piR-mmu-27200780 | P4ha3 | 0.958344 |
| piR-mmu-27200780 | Cxxc4 | 0.958044 |
| piR-mmu-27200780 | Arhgef40 | 0.956121 |
| piR-mmu-27200780 | Clmn | 0.955007 |
| piR-mmu-27200780 | Gpsm1 | 0.954684 |
| piR-mmu-27200780 | Tead1 | 0.954467 |
| piR-mmu-27200780 | Zdhhc8 | 0.953864 |
| piR-mmu-27200780 | Gm156 | 0.953153 |
| piR-mmu-27200780 | Lingo1 | 0.9531 |
| piR-mmu-27200780 | Kcna2 | 0.950525 |
| piR-mmu-27200780 | Iqub | 0.948972 |
| piR-mmu-27200780 | 4930503B20Rik | 0.948445 |
| piR-mmu-27200780 | Ano2 | 0.94778 |
| piR-mmu-27200780 | Elf4 | 0.947226 |
| piR-mmu-27200780 | Rgs20 | 0.946779 |
| piR-mmu-27200780 | Onecut3 | 0.946149 |
| piR-mmu-27200780 | Gm28551 | 0.945212 |
| piR-mmu-27200780 | Arrb2 | 0.944697 |
| piR-mmu-27200780 | Tbc1d19 | 0.944548 |
| piR-mmu-27200780 | Gtf2h1 | 0.944325 |
| piR-mmu-27200780 | Maml3 | 0.943988 |
| piR-mmu-27200780 | Pgr15l | 0.943579 |
| piR-mmu-27200780 | Cnfn | 0.943549 |
| piR-mmu-27200780 | Slc16a6 | 0.943443 |
| piR-mmu-27200780 | Ros1 | 0.943199 |
| piR-mmu-27200780 | Hyi | 0.9431 |
| piR-mmu-27200780 | 9930022D16Rik | 0.941926 |
| piR-mmu-27200780 | Tox | 0.941796 |
| piR-mmu-27200780 | Dhrs3 | 0.941774 |
| piR-mmu-27200780 | Cdkl5 | 0.940779 |

| | | |
|---|---|---|
| piR-mmu-27200780 | Daam1 | 0.939458 |
| piR-mmu-27200780 | Cyp2c37 | 0.938025 |
| piR-mmu-27200780 | Cyp2c54 | 0.937739 |
| piR-mmu-27200780 | Onecut2 | 0.937676 |
| piR-mmu-27200780 | Elavl4 | 0.937596 |
| piR-mmu-27200780 | Urgcp | 0.936885 |
| piR-mmu-27200780 | Tsr2 | 0.935913 |
| piR-mmu-27200780 | Myo7a | 0.935368 |
| piR-mmu-27200780 | Trpm3 | 0.933137 |
| piR-mmu-27200780 | Esrrg | 0.932706 |
| piR-mmu-27200780 | Trim36 | 0.931657 |
| piR-mmu-27200780 | Xrcc2 | 0.931609 |
| piR-mmu-27200780 | Zfp872 | 0.93147 |
| piR-mmu-27200780 | Cyp20a1 | 0.930963 |
| piR-mmu-27200780 | Slc39a2 | 0.930818 |
| piR-mmu-27200780 | Ifnar2 | 0.930813 |
| piR-mmu-27200780 | Slc37a2 | 0.930099 |
| piR-mmu-27200780 | Pter | 0.930074 |
| piR-mmu-27200780 | Ebf4 | 0.929851 |
| piR-mmu-27200780 | Chst10 | 0.929648 |
| piR-mmu-27200780 | Flt1 | 0.929489 |
| piR-mmu-27200780 | Crocc2 | 0.927885 |
| piR-mmu-27200780 | Rabl3 | 0.927297 |
| piR-mmu-27200780 | Rad54l2 | 0.926196 |
| piR-mmu-27200780 | Eda | 0.92564 |
| piR-mmu-27200780 | Slc16a13 | 0.92519 |
| piR-mmu-27200780 | A730049H05Rik | 0.925128 |
| piR-mmu-27200780 | Vwc2 | 0.924515 |
| piR-mmu-27200780 | Rhog | 0.924378 |
| piR-mmu-27200780 | Rsrp1 | 0.923577 |
| piR-mmu-27200780 | 2010005H15Rik | 0.923543 |
| piR-mmu-27200780 | Ebf1 | 0.923433 |
| piR-mmu-27200780 | Pank2 | 0.920579 |
| piR-mmu-27200780 | Cluh | 0.913915 |
| piR-mmu-27200780 | Atp1b3 | 0.913453 |
| piR-mmu-27200780 | Trpv3 | 0.912009 |
| piR-mmu-27200780 | Ssx2ip | 0.911956 |
| piR-mmu-27200780 | Arhgdig | 0.911354 |
| piR-mmu-27200780 | Wbscr16 | 0.910908 |
| piR-mmu-27200780 | Hid1 | 0.909527 |
| piR-mmu-27200780 | Kdm4b | 0.907706 |
| piR-mmu-27200780 | Mark4 | 0.907001 |
| piR-mmu-27200780 | Defb1 | 0.906051 |
| piR-mmu-27200780 | Fam90a1a | 0.90538 |
| piR-mmu-27200780 | Unc13d | 0.905256 |
| piR-mmu-27200780 | Fam117b | 0.904657 |
| piR-mmu-27200780 | Siae | 0.903771 |
| piR-mmu-27200780 | Tgfa | 0.901905 |
| piR-mmu-27200780 | Arhgef10l | 0.901132 |
| piR-mmu-27200780 | Spdye4b | 0.900557 |
| piR-mmu-27200780 | Efna5 | 0.900521 |
| piR-mmu-27200780 | Lyn | 0.900396 |
| piR-mmu-51362692 | Ptbp2 | 0.999699 |

| | | |
|---|---|---|
| piR-mmu-51362692 | Trim44 | 0.999414 |
| piR-mmu-51362692 | Arel1 | 0.998125 |
| piR-mmu-51362692 | Ctu1 | 0.998121 |
| piR-mmu-51362692 | B3galt2 | 0.997369 |
| piR-mmu-51362692 | Pcgf3 | 0.994857 |
| piR-mmu-51362692 | Esp1 | 0.992033 |
| piR-mmu-51362692 | Bcl2l2 | 0.991507 |
| piR-mmu-51362692 | Cpne9 | 0.990838 |
| piR-mmu-51362692 | Akap9 | 0.986022 |
| piR-mmu-51362692 | Eif2ak3 | 0.98464 |
| piR-mmu-51362692 | Plaur | 0.977936 |
| piR-mmu-51362692 | Luzp1 | 0.977278 |
| piR-mmu-51362692 | Pcnp | 0.975068 |
| piR-mmu-51362692 | Celf4 | 0.9671 |
| piR-mmu-51362692 | Slbp | 0.966931 |
| piR-mmu-51362692 | Ttn | 0.964351 |
| piR-mmu-51362692 | 6430550D23Rik | 0.962872 |
| piR-mmu-51362692 | Lyrm7 | 0.958159 |
| piR-mmu-51362692 | S100pbp | 0.954224 |
| piR-mmu-51362692 | Mtdh | 0.950626 |
| piR-mmu-51362692 | Srxn1 | 0.94743 |
| piR-mmu-51362692 | Zbtb20 | 0.94505 |
| piR-mmu-51362692 | Ahnak | 0.942885 |
| piR-mmu-51362692 | Grm5 | 0.93921 |
| piR-mmu-51362692 | Wnt4 | 0.937652 |
| piR-mmu-51362692 | Zcchc16 | 0.93645 |
| piR-mmu-51362692 | Kif3b | 0.935492 |
| piR-mmu-51362692 | Ptpdc1 | 0.931487 |
| piR-mmu-51362692 | Tnfrsf22 | 0.93022 |
| piR-mmu-51362692 | Rap2c | 0.925069 |
| piR-mmu-51362692 | Sort1 | 0.918444 |
| piR-mmu-51362692 | Xylb | 0.915127 |
| piR-mmu-51362692 | Gabpb2 | 0.910781 |
| piR-mmu-51362692 | Gda | 0.909671 |
| piR-mmu-51362692 | Gm42715 | 0.909067 |
| piR-mmu-51362692 | Nptxr | 0.908253 |
| piR-mmu-51362692 | Ip6k1 | 0.908204 |
| piR-mmu-51362692 | Pcgf2 | 0.906364 |
| piR-mmu-51362692 | 4921507P07Rik | 0.901564 |
| piR-mmu-4981268 | Gm17296 | 0.952552 |
| piR-mmu-4981268 | Clasrp | 0.927632 |
| piR-mmu-4981268 | Gm20683 | 0.906116 |
| piR-mmu-49714631 | Ddx5 | 0.999984 |
| piR-mmu-49714631 | Rbm46 | 0.999791 |
| piR-mmu-49714631 | Pank2 | 0.998941 |
| piR-mmu-49714631 | Nova1 | 0.99852 |
| piR-mmu-49714631 | D1Ertd622e | 0.997434 |
| piR-mmu-49714631 | Rab23 | 0.997281 |
| piR-mmu-49714631 | Eda | 0.996945 |
| piR-mmu-49714631 | Zfp672 | 0.996262 |
| piR-mmu-49714631 | Pde6d | 0.994929 |
| piR-mmu-49714631 | Wdr72 | 0.994493 |
| piR-mmu-49714631 | Tmem170 | 0.99364 |

| | | |
|---|---|---|
| piR-mmu-49714631 | Fyttd1 | 0.993189 |
| piR-mmu-49714631 | Gss | 0.992754 |
| piR-mmu-49714631 | Kcnmb4 | 0.991793 |
| piR-mmu-49714631 | Nrxn1 | 0.991366 |
| piR-mmu-49714631 | Ap1g1 | 0.988879 |
| piR-mmu-49714631 | A730049H05Rik | 0.987569 |
| piR-mmu-49714631 | Uvrag | 0.987543 |
| piR-mmu-49714631 | Tbck | 0.987328 |
| piR-mmu-49714631 | Tmem39b | 0.987327 |
| piR-mmu-49714631 | Dcaf5 | 0.986984 |
| piR-mmu-49714631 | Nfrkb | 0.985259 |
| piR-mmu-49714631 | Erbb4 | 0.983843 |
| piR-mmu-49714631 | Rassf3 | 0.983272 |
| piR-mmu-49714631 | Ankrd52 | 0.981524 |
| piR-mmu-49714631 | Senp1 | 0.980647 |
| piR-mmu-49714631 | Lancl3 | 0.976498 |
| piR-mmu-49714631 | C2cd3 | 0.97587 |
| piR-mmu-49714631 | Haus2 | 0.974621 |
| piR-mmu-49714631 | Tifab | 0.971987 |
| piR-mmu-49714631 | Brdt | 0.971449 |
| piR-mmu-49714631 | Gucy1b3 | 0.969355 |
| piR-mmu-49714631 | Chd2 | 0.968534 |
| piR-mmu-49714631 | Hnrnpa1 | 0.966438 |
| piR-mmu-49714631 | Poldip3 | 0.96602 |
| piR-mmu-49714631 | Krt80 | 0.965905 |
| piR-mmu-49714631 | Prdm10 | 0.964742 |
| piR-mmu-49714631 | Hells | 0.963979 |
| piR-mmu-49714631 | Cdc42bpg | 0.963426 |
| piR-mmu-49714631 | Xlr5c | 0.961553 |
| piR-mmu-49714631 | Ttc26 | 0.961402 |
| piR-mmu-49714631 | Usp7 | 0.960006 |
| piR-mmu-49714631 | Pcyt1a | 0.958738 |
| piR-mmu-49714631 | Rimkla | 0.957699 |
| piR-mmu-49714631 | Fam172a | 0.954955 |
| piR-mmu-49714631 | Egln3 | 0.953034 |
| piR-mmu-49714631 | Xlr5b | 0.952402 |
| piR-mmu-49714631 | Nphp3 | 0.949212 |
| piR-mmu-49714631 | Sobp | 0.948901 |
| piR-mmu-49714631 | Fbxl21 | 0.94866 |
| piR-mmu-49714631 | Phc1 | 0.944714 |
| piR-mmu-49714631 | Tead1 | 0.943241 |
| piR-mmu-49714631 | Ctgf | 0.943004 |
| piR-mmu-49714631 | Fam168a | 0.942938 |
| piR-mmu-49714631 | Fam35a | 0.939089 |
| piR-mmu-49714631 | Cadm4 | 0.937681 |
| piR-mmu-49714631 | Zhx2 | 0.937619 |
| piR-mmu-49714631 | Traf6 | 0.937307 |
| piR-mmu-49714631 | Kansl1l | 0.937146 |
| piR-mmu-49714631 | Pcdh20 | 0.935955 |
| piR-mmu-49714631 | Papola | 0.935754 |
| piR-mmu-49714631 | Bace1 | 0.935581 |
| piR-mmu-49714631 | Mfsd12 | 0.933297 |
| piR-mmu-49714631 | Pnn | 0.932339 |

| | | |
|---|---|---|
| piR-mmu-49714631 | Lhx2 | 0.932035 |
| piR-mmu-49714631 | Mapre1 | 0.927797 |
| piR-mmu-49714631 | Cxxc4 | 0.927727 |
| piR-mmu-49714631 | Tsr2 | 0.924254 |
| piR-mmu-49714631 | E330009J07Rik | 0.922849 |
| piR-mmu-49714631 | Col4a6 | 0.921772 |
| piR-mmu-49714631 | Pcdh17 | 0.921507 |
| piR-mmu-49714631 | Xlr5a | 0.921329 |
| piR-mmu-49714631 | Pdzd4 | 0.919901 |
| piR-mmu-49714631 | Rpl27 | 0.919686 |
| piR-mmu-49714631 | Taok1 | 0.919317 |
| piR-mmu-49714631 | Wdr38 | 0.918969 |
| piR-mmu-49714631 | Mettl20 | 0.917731 |
| piR-mmu-49714631 | Scaf4 | 0.916895 |
| piR-mmu-49714631 | D10Wsu102e | 0.914802 |
| piR-mmu-49714631 | Ermp1 | 0.914153 |
| piR-mmu-49714631 | Srgn | 0.913991 |
| piR-mmu-49714631 | Ypel4 | 0.913225 |
| piR-mmu-49714631 | Ascc2 | 0.912071 |
| piR-mmu-49714631 | Vangl1 | 0.911189 |
| piR-mmu-49714631 | Gk5 | 0.910855 |
| piR-mmu-49714631 | Ntmt1 | 0.90986 |
| piR-mmu-49714631 | Erp29 | 0.909838 |
| piR-mmu-49714631 | Mbtps2 | 0.909768 |
| piR-mmu-49714631 | Abi3 | 0.909553 |
| piR-mmu-49714631 | Ltbp1 | 0.909242 |
| piR-mmu-49714631 | Mafk | 0.909096 |
| piR-mmu-49714631 | Cluh | 0.907549 |
| piR-mmu-49714631 | Adamts16 | 0.906889 |
| piR-mmu-49714631 | Yy2 | 0.906341 |
| piR-mmu-49714631 | Mrps25 | 0.905782 |
| piR-mmu-49714631 | Defb1 | 0.905655 |
| piR-mmu-49714631 | Unc5c | 0.904208 |
| piR-mmu-49714631 | Naa20 | 0.902867 |
| piR-mmu-49714631 | Celf2 | 0.902096 |
| piR-mmu-49714631 | Tmprss11a | 0.901801 |
| piR-mmu-10991144 | 4930474N05Rik | 0.964658 |
| piR-mmu-10991144 | Hs6st2 | 0.955977 |
| piR-mmu-10991144 | Rab21 | 0.937803 |
| piR-mmu-10991144 | Rsf1 | 0.933653 |
| piR-mmu-10991144 | Zfp521 | 0.930989 |
| piR-mmu-10991144 | Fst | 0.927226 |
| piR-mmu-10991144 | Slc5a4b | 0.926282 |
| piR-mmu-10991144 | Esyt2 | 0.925877 |
| piR-mmu-10991144 | Gm4631 | 0.925451 |
| piR-mmu-10991144 | Ttn | 0.912427 |
| piR-mmu-10991144 | Gm17359 | 0.904966 |
| piR-mmu-5842895 | Gm17296 | 0.952552 |
| piR-mmu-5842895 | Clasrp | 0.928337 |
| piR-mmu-5842895 | Gm20683 | 0.906116 |
| piR-mmu-11031895 | 4930474N05Rik | 0.964711 |
| piR-mmu-11031895 | Hs6st2 | 0.954936 |
| piR-mmu-11031895 | Rab21 | 0.939913 |

| | | |
|---|---|---|
| piR-mmu-11031895 | Zfp521 | 0.93099 |
| piR-mmu-11031895 | Rsf1 | 0.929683 |
| piR-mmu-11031895 | Esyt2 | 0.927791 |
| piR-mmu-11031895 | Gm4631 | 0.92748 |
| piR-mmu-11031895 | Fst | 0.921751 |
| piR-mmu-11031895 | Ttn | 0.919292 |
| piR-mmu-11031895 | Slc5a4b | 0.911186 |
| piR-mmu-48806923 | Cstad | 0.999976 |
| piR-mmu-48806923 | Dok4 | 0.999965 |
| piR-mmu-48806923 | Neurod1 | 0.999846 |
| piR-mmu-48806923 | Nap1l3 | 0.999428 |
| piR-mmu-48806923 | Plpp7 | 0.998884 |
| piR-mmu-48806923 | Cml1 | 0.998821 |
| piR-mmu-48806923 | Fam134c | 0.997874 |
| piR-mmu-48806923 | Maob | 0.997814 |
| piR-mmu-48806923 | Cnih4 | 0.99683 |
| piR-mmu-48806923 | Smarce1 | 0.993794 |
| piR-mmu-48806923 | Opn1sw | 0.992157 |
| piR-mmu-48806923 | Cdk13 | 0.990467 |
| piR-mmu-48806923 | Pgr15l | 0.985202 |
| piR-mmu-48806923 | Ackr2 | 0.98378 |
| piR-mmu-48806923 | Ankrd13c | 0.983754 |
| piR-mmu-48806923 | Gm28551 | 0.981107 |
| piR-mmu-48806923 | Cnot4 | 0.980372 |
| piR-mmu-48806923 | Pim1 | 0.974604 |
| piR-mmu-48806923 | Thoc1 | 0.973756 |
| piR-mmu-48806923 | Tomm70a | 0.972967 |
| piR-mmu-48806923 | Lrp11 | 0.969201 |
| piR-mmu-48806923 | Cd300ld | 0.968258 |
| piR-mmu-48806923 | P4ha3 | 0.968061 |
| piR-mmu-48806923 | Zfand4 | 0.967918 |
| piR-mmu-48806923 | Gucy1a3 | 0.966591 |
| piR-mmu-48806923 | Pi15 | 0.965833 |
| piR-mmu-48806923 | Arid3a | 0.965208 |
| piR-mmu-48806923 | Gtf3c1 | 0.964513 |
| piR-mmu-48806923 | Pcdh7 | 0.963007 |
| piR-mmu-48806923 | Snx12 | 0.962962 |
| piR-mmu-48806923 | Cnot2 | 0.95914 |
| piR-mmu-48806923 | Cebpz | 0.958237 |
| piR-mmu-48806923 | Eda2r | 0.956388 |
| piR-mmu-48806923 | Gm12830 | 0.951931 |
| piR-mmu-48806923 | Ier5 | 0.95143 |
| piR-mmu-48806923 | Cdca4 | 0.947065 |
| piR-mmu-48806923 | Col6a6 | 0.946843 |
| piR-mmu-48806923 | Fam46a | 0.9464 |
| piR-mmu-48806923 | Arrb1 | 0.946293 |
| piR-mmu-48806923 | Gnao1 | 0.943226 |
| piR-mmu-48806923 | Fndc3b | 0.941373 |
| piR-mmu-48806923 | Thtpa | 0.938942 |
| piR-mmu-48806923 | Wfdc8 | 0.938931 |
| piR-mmu-48806923 | Evx1 | 0.936799 |
| piR-mmu-48806923 | Pdc | 0.935862 |
| piR-mmu-48806923 | Tox | 0.934487 |

| | | |
|---|---|---|
| piR-mmu-48806923 | Ankrd61 | 0.934391 |
| piR-mmu-48806923 | Chuk | 0.933936 |
| piR-mmu-48806923 | Spcs1 | 0.932329 |
| piR-mmu-48806923 | Nt5c1b | 0.93208 |
| piR-mmu-48806923 | Zfhx4 | 0.932017 |
| piR-mmu-48806923 | Tasp1 | 0.930568 |
| piR-mmu-48806923 | Zfp9 | 0.928029 |
| piR-mmu-48806923 | Rab31 | 0.924676 |
| piR-mmu-48806923 | Chka | 0.924502 |
| piR-mmu-48806923 | Ctnna1 | 0.924205 |
| piR-mmu-48806923 | Tmem184b | 0.922621 |
| piR-mmu-48806923 | Prtg | 0.921599 |
| piR-mmu-48806923 | Klf3 | 0.921372 |
| piR-mmu-48806923 | Gm5464 | 0.921059 |
| piR-mmu-48806923 | Zfp449 | 0.920564 |
| piR-mmu-48806923 | Insm1 | 0.918685 |
| piR-mmu-48806923 | Zranb2 | 0.915832 |
| piR-mmu-48806923 | Snai2 | 0.915033 |
| piR-mmu-48806923 | Patl1 | 0.914356 |
| piR-mmu-48806923 | Tubgcp3 | 0.913772 |
| piR-mmu-48806923 | Cdc6 | 0.912824 |
| piR-mmu-48806923 | Flt1 | 0.910826 |
| piR-mmu-48806923 | Slc23a1 | 0.90801 |
| piR-mmu-48806923 | Arhgap18 | 0.907856 |
| piR-mmu-48806923 | D1Ertd622e | 0.906527 |
| piR-mmu-48806923 | Abhd17b | 0.905802 |
| piR-mmu-48806923 | Mfsd7b | 0.903439 |
| piR-mmu-48806923 | Slc3a1 | 0.903227 |
| piR-mmu-48806923 | Tmem260 | 0.901858 |
| piR-mmu-48806923 | Gm10032 | 0.90057 |
| piR-mmu-10916133 | 4930474N05Rik | 0.96526 |
| piR-mmu-10916133 | Hs6st2 | 0.960285 |
| piR-mmu-10916133 | Gm17359 | 0.942076 |
| piR-mmu-10916133 | Rsf1 | 0.93921 |
| piR-mmu-10916133 | Ttn | 0.930116 |
| piR-mmu-10916133 | Zfp521 | 0.929307 |
| piR-mmu-10916133 | 1700019D03Rik | 0.926183 |
| piR-mmu-10916133 | Gm4631 | 0.92331 |
| piR-mmu-10916133 | Fst | 0.919237 |
| piR-mmu-10916133 | Esyt2 | 0.902416 |
| piR-mmu-3753105 | Marcks | 0.999999 |
| piR-mmu-3753105 | Ip6k2 | 0.999924 |
| piR-mmu-3753105 | Dab1 | 0.999724 |
| piR-mmu-3753105 | Atp5c1 | 0.999573 |
| piR-mmu-3753105 | Kcnd3 | 0.998683 |
| piR-mmu-3753105 | Bbx | 0.998524 |
| piR-mmu-3753105 | Dmd | 0.997627 |
| piR-mmu-3753105 | Gsx2 | 0.997048 |
| piR-mmu-3753105 | Gm9936 | 0.996766 |
| piR-mmu-3753105 | Stx5a | 0.994134 |
| piR-mmu-3753105 | Jph2 | 0.992976 |
| piR-mmu-3753105 | Phf21a | 0.991559 |
| piR-mmu-3753105 | Gm6793 | 0.991398 |

| | | |
|---|---|---|
| piR-mmu-3753105 | Xaf1 | 0.990855 |
| piR-mmu-3753105 | Snx4 | 0.988596 |
| piR-mmu-3753105 | Shb | 0.988363 |
| piR-mmu-3753105 | Kcnj6 | 0.986018 |
| piR-mmu-3753105 | Per1 | 0.984913 |
| piR-mmu-3753105 | Jchain | 0.984835 |
| piR-mmu-3753105 | Sav1 | 0.984479 |
| piR-mmu-3753105 | Mier3 | 0.984444 |
| piR-mmu-3753105 | Trp53inp1 | 0.982748 |
| piR-mmu-3753105 | Veph1 | 0.98222 |
| piR-mmu-3753105 | Il31ra | 0.981676 |
| piR-mmu-3753105 | Msi1 | 0.981043 |
| piR-mmu-3753105 | Lgi2 | 0.981 |
| piR-mmu-3753105 | Hcrtr2 | 0.980582 |
| piR-mmu-3753105 | Rcn2 | 0.980384 |
| piR-mmu-3753105 | Nelfa | 0.979441 |
| piR-mmu-3753105 | Pcmtd2 | 0.97926 |
| piR-mmu-3753105 | Ctage5 | 0.978253 |
| piR-mmu-3753105 | Gm4841 | 0.976135 |
| piR-mmu-3753105 | Olfr862 | 0.974037 |
| piR-mmu-3753105 | Ywhab | 0.973941 |
| piR-mmu-3753105 | Taf4a | 0.96821 |
| piR-mmu-3753105 | Fat1 | 0.967769 |
| piR-mmu-3753105 | Nlgn1 | 0.966645 |
| piR-mmu-3753105 | Cbln2 | 0.964817 |
| piR-mmu-3753105 | Tnfrsf22 | 0.964227 |
| piR-mmu-3753105 | Duxbl3 | 0.96327 |
| piR-mmu-3753105 | Duxbl2 | 0.96327 |
| piR-mmu-3753105 | Duxbl1 | 0.960473 |
| piR-mmu-3753105 | Nol4l | 0.960378 |
| piR-mmu-3753105 | Cpne1 | 0.959567 |
| piR-mmu-3753105 | Atg7 | 0.959365 |
| piR-mmu-3753105 | Creb1 | 0.958924 |
| piR-mmu-3753105 | 4930568D16Rik | 0.957942 |
| piR-mmu-3753105 | 1700034J05Rik | 0.95608 |
| piR-mmu-3753105 | Zfp652 | 0.955073 |
| piR-mmu-3753105 | Otud7b | 0.950845 |
| piR-mmu-3753105 | Smoc2 | 0.950802 |
| piR-mmu-3753105 | Gm10392 | 0.945049 |
| piR-mmu-3753105 | Hoxc9 | 0.943608 |
| piR-mmu-3753105 | Samd4b | 0.943144 |
| piR-mmu-3753105 | Mapk1ip1l | 0.942263 |
| piR-mmu-3753105 | Phlpp1 | 0.941662 |
| piR-mmu-3753105 | Mga | 0.940309 |
| piR-mmu-3753105 | Ces1h | 0.940024 |
| piR-mmu-3753105 | Sox1 | 0.938151 |
| piR-mmu-3753105 | Col16a1 | 0.936471 |
| piR-mmu-3753105 | Ifitm10 | 0.936019 |
| piR-mmu-3753105 | Btla | 0.93577 |
| piR-mmu-3753105 | Gps1 | 0.934299 |
| piR-mmu-3753105 | Ipcef1 | 0.931129 |
| piR-mmu-3753105 | Henmt1 | 0.929815 |
| piR-mmu-3753105 | Noct | 0.929324 |

| | | |
|---|---|---|
| piR-mmu-3753105 | Kcnb1 | 0.927377 |
| piR-mmu-3753105 | Apba1 | 0.927356 |
| piR-mmu-3753105 | Styk1 | 0.927115 |
| piR-mmu-3753105 | Timm21 | 0.926077 |
| piR-mmu-3753105 | Ap1m2 | 0.925835 |
| piR-mmu-3753105 | Gramd1b | 0.924519 |
| piR-mmu-3753105 | Etf1 | 0.92368 |
| piR-mmu-3753105 | Ggcx | 0.923354 |
| piR-mmu-3753105 | Dld | 0.921223 |
| piR-mmu-3753105 | Rps15a | 0.921084 |
| piR-mmu-3753105 | Pla2r1 | 0.919068 |
| piR-mmu-3753105 | Psat1 | 0.918838 |
| piR-mmu-3753105 | Gak | 0.918585 |
| piR-mmu-3753105 | Vps37a | 0.917308 |
| piR-mmu-3753105 | Rnf217 | 0.916865 |
| piR-mmu-3753105 | Pycard | 0.916589 |
| piR-mmu-3753105 | Bahcc1 | 0.916253 |
| piR-mmu-3753105 | Cdc27 | 0.916107 |
| piR-mmu-3753105 | Glipr2 | 0.916007 |
| piR-mmu-3753105 | Edc3 | 0.913592 |
| piR-mmu-3753105 | Ptch1 | 0.911664 |
| piR-mmu-3753105 | Adam19 | 0.911364 |
| piR-mmu-3753105 | 5330417C22Rik | 0.910947 |
| piR-mmu-3753105 | Ephb2 | 0.910385 |
| piR-mmu-3753105 | 1700019D03Rik | 0.909934 |
| piR-mmu-3753105 | Iffo1 | 0.909222 |
| piR-mmu-3753105 | Il10 | 0.908856 |
| piR-mmu-3753105 | A830005F24Rik | 0.908779 |
| piR-mmu-3753105 | Nudt8 | 0.908737 |
| piR-mmu-3753105 | Ndufa10 | 0.908227 |
| piR-mmu-3753105 | Cilp | 0.906448 |
| piR-mmu-3753105 | Sp4 | 0.903745 |
| piR-mmu-3753105 | Sfr1 | 0.902508 |
| piR-mmu-2692796 | Atxn1 | 0.999548 |
| piR-mmu-2692796 | Pknox2 | 0.99949 |
| piR-mmu-2692796 | Kbtbd7 | 0.999445 |
| piR-mmu-2692796 | Aldh1a7 | 0.994952 |
| piR-mmu-2692796 | Osbp | 0.992683 |
| piR-mmu-2692796 | Trpm6 | 0.989434 |
| piR-mmu-2692796 | Sema3a | 0.988851 |
| piR-mmu-2692796 | Sirt1 | 0.988841 |
| piR-mmu-2692796 | Rbm25 | 0.983647 |
| piR-mmu-2692796 | Ldhb | 0.982995 |
| piR-mmu-2692796 | Gm15262 | 0.979064 |
| piR-mmu-2692796 | Atrn | 0.972107 |
| piR-mmu-2692796 | Carf | 0.969356 |
| piR-mmu-2692796 | Kdm3b | 0.967188 |
| piR-mmu-2692796 | D1Ertd622e | 0.966198 |
| piR-mmu-2692796 | Rpa1 | 0.962014 |
| piR-mmu-2692796 | Lilr4b | 0.958205 |
| piR-mmu-2692796 | Maml3 | 0.957547 |
| piR-mmu-2692796 | Zfp563 | 0.955701 |
| piR-mmu-2692796 | Rps6ka3 | 0.951426 |

| | | |
|---|---|---|
| piR-mmu-2692796 | Eid1 | 0.950784 |
| piR-mmu-2692796 | Vapa | 0.947063 |
| piR-mmu-2692796 | Ip6k1 | 0.944148 |
| piR-mmu-2692796 | Ephb1 | 0.940758 |
| piR-mmu-2692796 | Klrk1 | 0.940064 |
| piR-mmu-2692796 | Srsf10 | 0.939897 |
| piR-mmu-2692796 | 3110043O21Rik | 0.927635 |
| piR-mmu-2692796 | Soga1 | 0.927247 |
| piR-mmu-2692796 | Olfr20 | 0.924793 |
| piR-mmu-2692796 | Scgb2b19 | 0.92263 |
| piR-mmu-2692796 | Scgb2b12 | 0.92263 |
| piR-mmu-2692796 | Plppr3 | 0.915125 |
| piR-mmu-2692796 | Enam | 0.910283 |
| piR-mmu-2692796 | Rasgef1b | 0.908846 |
| piR-mmu-2692796 | Ddi2 | 0.907968 |
| piR-mmu-2692796 | Fgl2 | 0.906623 |
| piR-mmu-2692796 | Reg3a | 0.905848 |
| piR-mmu-2692796 | Atp6v1h | 0.90348 |
| piR-mmu-2692796 | Cttnbp2 | 0.903213 |
| piR-mmu-2692796 | C330011M18Rik | 0.900693 |
| piR-mmu-12633865 | Ddx3x | 0.990541 |
| piR-mmu-12633865 | Ogt | 0.983662 |
| piR-mmu-12633865 | Etnk1 | 0.95763 |
| piR-mmu-12633865 | Dennd5b | 0.957621 |
| piR-mmu-12633865 | Gclm | 0.956544 |
| piR-mmu-12633865 | Nfat5 | 0.956436 |
| piR-mmu-12633865 | Gm15140 | 0.955634 |
| piR-mmu-12633865 | Igf1r | 0.947746 |
| piR-mmu-12633865 | Myh3 | 0.94207 |
| piR-mmu-12633865 | Rita1 | 0.938069 |
| piR-mmu-12633865 | Zfand3 | 0.93805 |
| piR-mmu-12633865 | Anapc11 | 0.936925 |
| piR-mmu-12633865 | Pcdhb9 | 0.936582 |
| piR-mmu-12633865 | Mxi1 | 0.936326 |
| piR-mmu-12633865 | Ldah | 0.934387 |
| piR-mmu-12633865 | Rgs17 | 0.931993 |
| piR-mmu-12633865 | Ptprd | 0.931007 |
| piR-mmu-12633865 | Zak | 0.930291 |
| piR-mmu-12633865 | Ctdspl2 | 0.93025 |
| piR-mmu-12633865 | Tmem251 | 0.93022 |
| piR-mmu-12633865 | Hibadh | 0.928289 |
| piR-mmu-12633865 | Meis1 | 0.920848 |
| piR-mmu-12633865 | Onecut1 | 0.920426 |
| piR-mmu-12633865 | D1Ertd622e | 0.918748 |
| piR-mmu-12633865 | Fam134a | 0.917391 |
| piR-mmu-12633865 | Lcp2 | 0.912072 |
| piR-mmu-12633865 | Zbtb20 | 0.908065 |
| piR-mmu-12633865 | Plcz1 | 0.902677 |
| piR-mmu-12633865 | Rnf217 | 0.900176 |
| piR-mmu-48882277 | Ddx5 | 0.999989 |
| piR-mmu-48882277 | Tmem170 | 0.999821 |
| piR-mmu-48882277 | Nova1 | 0.999708 |
| piR-mmu-48882277 | Eda | 0.999224 |

| | | |
|---|---|---|
| piR-mmu-48882277 | Fyttd1 | 0.998952 |
| piR-mmu-48882277 | Brdt | 0.998925 |
| piR-mmu-48882277 | Rab23 | 0.998838 |
| piR-mmu-48882277 | Wdr72 | 0.998742 |
| piR-mmu-48882277 | Pank2 | 0.998428 |
| piR-mmu-48882277 | Zfp672 | 0.99833 |
| piR-mmu-48882277 | D1Ertd622e | 0.998108 |
| piR-mmu-48882277 | Gss | 0.997769 |
| piR-mmu-48882277 | Pde6d | 0.995714 |
| piR-mmu-48882277 | C2cd3 | 0.995074 |
| piR-mmu-48882277 | Zhx2 | 0.994869 |
| piR-mmu-48882277 | Nrxn1 | 0.994735 |
| piR-mmu-48882277 | Ap1g1 | 0.99369 |
| piR-mmu-48882277 | Uvrag | 0.991617 |
| piR-mmu-48882277 | Brinp3 | 0.988877 |
| piR-mmu-48882277 | Dcaf5 | 0.988537 |
| piR-mmu-48882277 | Cacna1h | 0.988491 |
| piR-mmu-48882277 | Nfrkb | 0.988127 |
| piR-mmu-48882277 | Rimkla | 0.98777 |
| piR-mmu-48882277 | Ankrd52 | 0.987583 |
| piR-mmu-48882277 | A730049H05Rik | 0.986148 |
| piR-mmu-48882277 | Erbb4 | 0.985718 |
| piR-mmu-48882277 | Ttc26 | 0.985347 |
| piR-mmu-48882277 | Tifab | 0.983906 |
| piR-mmu-48882277 | Rassf3 | 0.983852 |
| piR-mmu-48882277 | Senp1 | 0.983257 |
| piR-mmu-48882277 | Maob | 0.982621 |
| piR-mmu-48882277 | Smc3 | 0.982401 |
| piR-mmu-48882277 | Gucy1b3 | 0.977714 |
| piR-mmu-48882277 | Nyap2 | 0.977508 |
| piR-mmu-48882277 | Kcnmb4 | 0.976363 |
| piR-mmu-48882277 | Cyp20a1 | 0.975147 |
| piR-mmu-48882277 | Tbck | 0.973956 |
| piR-mmu-48882277 | Mapre1 | 0.973559 |
| piR-mmu-48882277 | Nphp3 | 0.972268 |
| piR-mmu-48882277 | Phc1 | 0.971042 |
| piR-mmu-48882277 | Pcyt1a | 0.970422 |
| piR-mmu-48882277 | Cadm4 | 0.969573 |
| piR-mmu-48882277 | Egln3 | 0.969278 |
| piR-mmu-48882277 | Yy2 | 0.968039 |
| piR-mmu-48882277 | Mbtps2 | 0.967611 |
| piR-mmu-48882277 | Usp7 | 0.966406 |
| piR-mmu-48882277 | Bace1 | 0.965188 |
| piR-mmu-48882277 | Man2a2 | 0.96438 |
| piR-mmu-48882277 | Hnrnpa1 | 0.964108 |
| piR-mmu-48882277 | Prdm10 | 0.964085 |
| piR-mmu-48882277 | Haus2 | 0.961888 |
| piR-mmu-48882277 | Ypel4 | 0.961836 |
| piR-mmu-48882277 | Hells | 0.961616 |
| piR-mmu-48882277 | Fbxl21 | 0.96084 |
| piR-mmu-48882277 | Fam168a | 0.959735 |
| piR-mmu-48882277 | Zfp398 | 0.959157 |
| piR-mmu-48882277 | Ip6k2 | 0.958618 |

| | | |
|---|---|---|
| piR-mmu-48882277 | Fam172a | 0.958424 |
| piR-mmu-48882277 | Tmem39b | 0.953937 |
| piR-mmu-48882277 | Slc15a1 | 0.950638 |
| piR-mmu-48882277 | Xlr5c | 0.95037 |
| piR-mmu-48882277 | Traf6 | 0.95009 |
| piR-mmu-48882277 | Ntmt1 | 0.949501 |
| piR-mmu-48882277 | Rpl27 | 0.949231 |
| piR-mmu-48882277 | Hrnr | 0.949066 |
| piR-mmu-48882277 | Zbtb34 | 0.948819 |
| piR-mmu-48882277 | Poldip3 | 0.948069 |
| piR-mmu-48882277 | Lancl3 | 0.9474 |
| piR-mmu-48882277 | Sobp | 0.946969 |
| piR-mmu-48882277 | Fgfr1 | 0.946925 |
| piR-mmu-48882277 | Map3k7 | 0.945109 |
| piR-mmu-48882277 | Cdc42bpg | 0.943946 |
| piR-mmu-48882277 | Unc5d | 0.943646 |
| piR-mmu-48882277 | Abi3 | 0.942492 |
| piR-mmu-48882277 | Wdr38 | 0.94192 |
| piR-mmu-48882277 | Tead1 | 0.9417 |
| piR-mmu-48882277 | Mafk | 0.941395 |
| piR-mmu-48882277 | Lhx2 | 0.941334 |
| piR-mmu-48882277 | Ccpg1 | 0.940944 |
| piR-mmu-48882277 | Scaf4 | 0.940387 |
| piR-mmu-48882277 | Prtg | 0.939653 |
| piR-mmu-48882277 | Xlr5b | 0.939473 |
| piR-mmu-48882277 | Pgr15l | 0.939157 |
| piR-mmu-48882277 | Shpk | 0.937834 |
| piR-mmu-48882277 | Adamtsl3 | 0.935626 |
| piR-mmu-48882277 | Ocln | 0.934619 |
| piR-mmu-48882277 | Ermp1 | 0.93446 |
| piR-mmu-48882277 | Pcdh20 | 0.930862 |
| piR-mmu-48882277 | C2cd2 | 0.929688 |
| piR-mmu-48882277 | Map4k2 | 0.929666 |
| piR-mmu-48882277 | Cxxc4 | 0.926782 |
| piR-mmu-48882277 | Taok1 | 0.926322 |
| piR-mmu-48882277 | Sfxn4 | 0.925786 |
| piR-mmu-48882277 | Pcdh17 | 0.92541 |
| piR-mmu-48882277 | Gm10447 | 0.925283 |
| piR-mmu-48882277 | Papola | 0.924884 |
| piR-mmu-48882277 | Col4a6 | 0.924175 |
| piR-mmu-48882277 | Dip2b | 0.924002 |
| piR-mmu-48882277 | Rbm46 | 0.923745 |
| piR-mmu-48882277 | Mettl20 | 0.92303 |
| piR-mmu-48882277 | RP23-95K12.13 | 0.92219 |
| piR-mmu-48882277 | Daam2 | 0.922057 |
| piR-mmu-48882277 | Mfsd12 | 0.921424 |
| piR-mmu-48882277 | Homer2 | 0.921337 |
| piR-mmu-48882277 | Kbtbd2 | 0.921122 |
| piR-mmu-48882277 | Tasp1 | 0.917544 |
| piR-mmu-48882277 | Tox4 | 0.915544 |
| piR-mmu-48882277 | Tsr2 | 0.915294 |
| piR-mmu-48882277 | Srp72 | 0.91509 |
| piR-mmu-48882277 | Mecp2 | 0.914881 |

| | | |
|---|---|---|
| piR-mmu-48882277 | Pdzd4 | 0.914863 |
| piR-mmu-48882277 | Nop58 | 0.91393 |
| piR-mmu-48882277 | Erp29 | 0.91317 |
| piR-mmu-48882277 | Fam35a | 0.913153 |
| piR-mmu-48882277 | Unc5c | 0.912396 |
| piR-mmu-48882277 | Ctgf | 0.911934 |
| piR-mmu-48882277 | Vmn2r34 | 0.910549 |
| piR-mmu-48882277 | E330009J07Rik | 0.910468 |
| piR-mmu-48882277 | Bpnt1 | 0.909242 |
| piR-mmu-48882277 | Vmn2r45 | 0.909161 |
| piR-mmu-48882277 | Tmem251 | 0.909066 |
| piR-mmu-48882277 | Pak6 | 0.907907 |
| piR-mmu-48882277 | Tmprss11a | 0.905509 |
| piR-mmu-48882277 | D17H6S53E | 0.904789 |
| piR-mmu-48882277 | Defb1 | 0.904475 |
| piR-mmu-48882277 | Xlr5a | 0.903662 |
| piR-mmu-48882277 | Rpl27-ps3 | 0.903312 |
| piR-mmu-48882277 | Pex13 | 0.902965 |
| piR-mmu-48882277 | Necab2 | 0.902637 |
| piR-mmu-48882277 | Txndc11 | 0.901476 |
| piR-mmu-48882277 | Baz1b | 0.901317 |
| piR-mmu-48882277 | Pnn | 0.901087 |
| piR-mmu-48882277 | Cluh | 0.900384 |
| piR-mmu-48882277 | Onecut2 | 0.900204 |
| piR-mmu-8507752 | Gm17296 | 0.952552 |
| piR-mmu-8507752 | Clasrp | 0.928337 |
| piR-mmu-8507752 | Gm20683 | 0.906116 |
| piR-mmu-2678950 | Ip6k2 | 0.999991 |
| piR-mmu-2678950 | Marcks | 0.999908 |
| piR-mmu-2678950 | Bbx | 0.999531 |
| piR-mmu-2678950 | Dab1 | 0.997139 |
| piR-mmu-2678950 | Stx5a | 0.996774 |
| piR-mmu-2678950 | Gm9936 | 0.996663 |
| piR-mmu-2678950 | Veph1 | 0.994281 |
| piR-mmu-2678950 | Nelfa | 0.994272 |
| piR-mmu-2678950 | Ctage5 | 0.994095 |
| piR-mmu-2678950 | Pcmtd2 | 0.99375 |
| piR-mmu-2678950 | Trp53inp1 | 0.993688 |
| piR-mmu-2678950 | Atp5c1 | 0.992928 |
| piR-mmu-2678950 | Sav1 | 0.991424 |
| piR-mmu-2678950 | Xaf1 | 0.991362 |
| piR-mmu-2678950 | Kcnd3 | 0.990989 |
| piR-mmu-2678950 | Gsx2 | 0.988386 |
| piR-mmu-2678950 | Gm4841 | 0.98786 |
| piR-mmu-2678950 | Per1 | 0.984677 |
| piR-mmu-2678950 | Kcnj6 | 0.984461 |
| piR-mmu-2678950 | 4930568D16Rik | 0.982656 |
| piR-mmu-2678950 | Jph2 | 0.982047 |
| piR-mmu-2678950 | Duxbl3 | 0.979692 |
| piR-mmu-2678950 | Duxbl2 | 0.979692 |
| piR-mmu-2678950 | Rbm24 | 0.978867 |
| piR-mmu-2678950 | Mga | 0.978843 |
| piR-mmu-2678950 | Duxbl1 | 0.977886 |

| | | |
|---|---|---|
| piR-mmu-2678950 | Med26 | 0.972048 |
| piR-mmu-2678950 | Nlgn1 | 0.968285 |
| piR-mmu-2678950 | Kcnb1 | 0.968119 |
| piR-mmu-2678950 | Atg7 | 0.967758 |
| piR-mmu-2678950 | Mier3 | 0.967683 |
| piR-mmu-2678950 | Il31ra | 0.963965 |
| piR-mmu-2678950 | Apoa2 | 0.95879 |
| piR-mmu-2678950 | Sox1 | 0.957951 |
| piR-mmu-2678950 | Apba1 | 0.957154 |
| piR-mmu-2678950 | Snx4 | 0.956588 |
| piR-mmu-2678950 | Dld | 0.956223 |
| piR-mmu-2678950 | Rap1a | 0.955732 |
| piR-mmu-2678950 | Nol4l | 0.953005 |
| piR-mmu-2678950 | Ldoc1l | 0.952467 |
| piR-mmu-2678950 | Ubtf | 0.948601 |
| piR-mmu-2678950 | Nr2c2 | 0.947488 |
| piR-mmu-2678950 | Phf21a | 0.947267 |
| piR-mmu-2678950 | Ipcef1 | 0.945656 |
| piR-mmu-2678950 | Gm10392 | 0.945375 |
| piR-mmu-2678950 | 1700017B05Rik | 0.94469 |
| piR-mmu-2678950 | Msi1 | 0.942478 |
| piR-mmu-2678950 | Zbtb4 | 0.941064 |
| piR-mmu-2678950 | Lgi2 | 0.940021 |
| piR-mmu-2678950 | Col16a1 | 0.939401 |
| piR-mmu-2678950 | Abcb9 | 0.938735 |
| piR-mmu-2678950 | Olfr862 | 0.937269 |
| piR-mmu-2678950 | Ifitm10 | 0.937187 |
| piR-mmu-2678950 | Zbed4 | 0.93649 |
| piR-mmu-2678950 | Smoc2 | 0.935357 |
| piR-mmu-2678950 | A330050F15Rik | 0.933149 |
| piR-mmu-2678950 | Gramd1b | 0.93221 |
| piR-mmu-2678950 | Kcnk13 | 0.93051 |
| piR-mmu-2678950 | Slc4a3 | 0.929635 |
| piR-mmu-2678950 | Gps2 | 0.928614 |
| piR-mmu-2678950 | Edc3 | 0.927676 |
| piR-mmu-2678950 | Galk2 | 0.925769 |
| piR-mmu-2678950 | Spag9 | 0.925583 |
| piR-mmu-2678950 | Etf1 | 0.92481 |
| piR-mmu-2678950 | Rnf34 | 0.924778 |
| piR-mmu-2678950 | Samd4b | 0.921632 |
| piR-mmu-2678950 | Cilp | 0.921439 |
| piR-mmu-2678950 | Styk1 | 0.919481 |
| piR-mmu-2678950 | Cpne1 | 0.918356 |
| piR-mmu-2678950 | Cbln2 | 0.917746 |
| piR-mmu-2678950 | Hcrtr2 | 0.915609 |
| piR-mmu-2678950 | Bahcc1 | 0.914674 |
| piR-mmu-2678950 | Cd40lg | 0.913623 |
| piR-mmu-2678950 | Otud7b | 0.913273 |
| piR-mmu-2678950 | Ralgps2 | 0.911479 |
| piR-mmu-2678950 | Fkbp7 | 0.910822 |
| piR-mmu-2678950 | Elf5 | 0.910454 |
| piR-mmu-2678950 | Creb1 | 0.909443 |
| piR-mmu-2678950 | Tnfrsf22 | 0.909094 |

| | | |
|---|---|---|
| piR-mmu-2678950 | A830005F24Rik | 0.908779 |
| piR-mmu-2678950 | Rcn2 | 0.908286 |
| piR-mmu-2678950 | Dmd | 0.905903 |
| piR-mmu-2678950 | Wnk1 | 0.904599 |
| piR-mmu-2678950 | BC106179 | 0.904346 |
| piR-mmu-2678950 | Nt5e | 0.90412 |
| piR-mmu-2678950 | E2f8 | 0.904014 |
| piR-mmu-2678950 | Sp4 | 0.903745 |
| piR-mmu-2678950 | Hoxc9 | 0.902644 |
| piR-mmu-2678950 | Hspa1b | 0.902251 |
| piR-mmu-2678950 | Lars2 | 0.901629 |
| piR-mmu-2678950 | Timm21 | 0.901625 |
| piR-mmu-2678950 | Tmem8b | 0.901185 |
| piR-mmu-2678950 | Ddx4 | 0.900926 |
| piR-mmu-2678950 | Gpr85 | 0.900593 |
| piR-mmu-2678950 | Gps1 | 0.900476 |
| piR-mmu-49342972 | Slc5a4b | 0.975837 |
| piR-mmu-49342972 | 4930474N05Rik | 0.965746 |
| piR-mmu-49342972 | Hs6st2 | 0.961401 |
| piR-mmu-49342972 | Esyt2 | 0.958955 |
| piR-mmu-49342972 | Rsf1 | 0.935427 |
| piR-mmu-49342972 | Rab21 | 0.933889 |
| piR-mmu-49342972 | Zfp521 | 0.932789 |
| piR-mmu-49342972 | Gm4631 | 0.92408 |
| piR-mmu-49342972 | Fst | 0.922605 |
| piR-mmu-49342972 | Cmah | 0.91936 |
| piR-mmu-49342972 | Ttn | 0.918987 |
| piR-mmu-49342972 | Gm17359 | 0.904966 |
| piR-mmu-10934588 | Slc5a4b | 0.977283 |
| piR-mmu-10934588 | 4930474N05Rik | 0.965579 |
| piR-mmu-10934588 | Esyt2 | 0.962716 |
| piR-mmu-10934588 | Hs6st2 | 0.956221 |
| piR-mmu-10934588 | Rab21 | 0.944848 |
| piR-mmu-10934588 | Rsf1 | 0.934418 |
| piR-mmu-10934588 | Zfp521 | 0.930588 |
| piR-mmu-10934588 | Gm4631 | 0.926415 |
| piR-mmu-10934588 | Fst | 0.921106 |
| piR-mmu-10934588 | Cmah | 0.920454 |
| piR-mmu-10934588 | 1700019D03Rik | 0.9165 |
| piR-mmu-10934588 | Ttn | 0.912102 |
| piR-mmu-10934588 | Chka | 0.90841 |
| piR-mmu-10934588 | Gm17359 | 0.902237 |
| piR-mmu-10912700 | 4930474N05Rik | 0.964606 |
| piR-mmu-10912700 | Hs6st2 | 0.957198 |
| piR-mmu-10912700 | Rsf1 | 0.936375 |
| piR-mmu-10912700 | Zfp521 | 0.933009 |
| piR-mmu-10912700 | Rab21 | 0.929145 |
| piR-mmu-10912700 | 1700019D03Rik | 0.928145 |
| piR-mmu-10912700 | Slc5a4b | 0.926282 |
| piR-mmu-10912700 | Gm4631 | 0.924724 |
| piR-mmu-10912700 | Ttn | 0.924376 |
| piR-mmu-10912700 | Fst | 0.922547 |
| piR-mmu-10912700 | Chka | 0.916729 |

| | | |
|---|---|---|
| piR-mmu-10912700 | Esyt2 | 0.907793 |
| piR-mmu-10912700 | Gm17359 | 0.906987 |
| piR-mmu-11461014 | 4930474N05Rik | 0.964431 |
| piR-mmu-11461014 | Hs6st2 | 0.955028 |
| piR-mmu-11461014 | Rab21 | 0.936214 |
| piR-mmu-11461014 | 1700019D03Rik | 0.932658 |
| piR-mmu-11461014 | Rsf1 | 0.929049 |
| piR-mmu-11461014 | Zfp521 | 0.927983 |
| piR-mmu-11461014 | Slc5a4b | 0.926282 |
| piR-mmu-11461014 | Fst | 0.924896 |
| piR-mmu-11461014 | Gm4631 | 0.923684 |
| piR-mmu-11461014 | Ttn | 0.923388 |
| piR-mmu-11461014 | Chka | 0.913143 |
| piR-mmu-11461014 | Gm17359 | 0.90086 |
| piR-mmu-10991141 | 4930474N05Rik | 0.964431 |
| piR-mmu-10991141 | Hs6st2 | 0.956247 |
| piR-mmu-10991141 | Rsf1 | 0.936713 |
| piR-mmu-10991141 | Zfp521 | 0.931272 |
| piR-mmu-10991141 | 1700019D03Rik | 0.929688 |
| piR-mmu-10991141 | Ttn | 0.928505 |
| piR-mmu-10991141 | Rab21 | 0.927637 |
| piR-mmu-10991141 | Slc5a4b | 0.926282 |
| piR-mmu-10991141 | Gm4631 | 0.923458 |
| piR-mmu-10991141 | Fst | 0.922547 |
| piR-mmu-10991141 | Chka | 0.913091 |
| piR-mmu-10991141 | Gm17359 | 0.908324 |
| piR-mmu-11031886 | 4930474N05Rik | 0.964793 |
| piR-mmu-11031886 | Hs6st2 | 0.957279 |
| piR-mmu-11031886 | Rsf1 | 0.937353 |
| piR-mmu-11031886 | Zfp521 | 0.930111 |
| piR-mmu-11031886 | Rab21 | 0.929954 |
| piR-mmu-11031886 | Slc5a4b | 0.926282 |
| piR-mmu-11031886 | Ttn | 0.924288 |
| piR-mmu-11031886 | Fst | 0.924255 |
| piR-mmu-11031886 | Gm4631 | 0.923247 |
| piR-mmu-11031886 | Esyt2 | 0.922309 |
| piR-mmu-11031886 | Chka | 0.913632 |
| piR-mmu-11031886 | Gm17359 | 0.904966 |
| piR-mmu-49609791 | Ctla4 | 0.992971 |
| piR-mmu-49609791 | Kbtbd7 | 0.987621 |
| piR-mmu-49609791 | Pgm2l1 | 0.986001 |
| piR-mmu-49609791 | Fstl1 | 0.984275 |
| piR-mmu-49609791 | Fam175a | 0.965769 |
| piR-mmu-49609791 | Tnfrsf26 | 0.956257 |
| piR-mmu-49609791 | Vipr2 | 0.954048 |
| piR-mmu-49609791 | Ubfd1 | 0.951074 |
| piR-mmu-49609791 | Elavl4 | 0.947465 |
| piR-mmu-49609791 | Rnf128 | 0.947073 |
| piR-mmu-49609791 | Macrod2 | 0.94592 |
| piR-mmu-49609791 | Cd276 | 0.944693 |
| piR-mmu-49609791 | Alg10b | 0.938607 |
| piR-mmu-49609791 | Trip11 | 0.938588 |
| piR-mmu-49609791 | S100a14 | 0.933324 |

| | | |
|---|---|---|
| piR-mmu-49609791 | Kras | 0.930639 |
| piR-mmu-49609791 | Runx2 | 0.9306 |
| piR-mmu-49609791 | Fer | 0.930343 |
| piR-mmu-49609791 | Asah2 | 0.928413 |
| piR-mmu-49609791 | Smim7 | 0.927938 |
| piR-mmu-49609791 | Grin2b | 0.925851 |
| piR-mmu-49609791 | Fmo6 | 0.925493 |
| piR-mmu-49609791 | Bend3 | 0.923208 |
| piR-mmu-49609791 | Zfp106 | 0.922686 |
| piR-mmu-49609791 | Zfp746 | 0.919535 |
| piR-mmu-49609791 | Gm4847 | 0.919469 |
| piR-mmu-49609791 | Sorl1 | 0.918157 |
| piR-mmu-49609791 | Lipn | 0.915828 |
| piR-mmu-49609791 | Pou2f1 | 0.914618 |
| piR-mmu-49609791 | Oas1f | 0.912813 |
| piR-mmu-49609791 | Rnf141 | 0.910337 |
| piR-mmu-49609791 | D330045A20Rik | 0.906262 |
| piR-mmu-49609791 | Epha7 | 0.905559 |
| piR-mmu-49609791 | Gm498 | 0.903676 |
| piR-mmu-49659703 | 4930474N05Rik | 0.965417 |
| piR-mmu-49659703 | Hs6st2 | 0.959074 |
| piR-mmu-49659703 | Rsf1 | 0.937641 |
| piR-mmu-49659703 | Gm17359 | 0.932927 |
| piR-mmu-49659703 | Zfp521 | 0.932469 |
| piR-mmu-49659703 | Spink8 | 0.930354 |
| piR-mmu-49659703 | Ttn | 0.926219 |
| piR-mmu-49659703 | Gm4631 | 0.925974 |
| piR-mmu-49659703 | Esyt2 | 0.923495 |
| piR-mmu-49659703 | Fst | 0.921816 |
| piR-mmu-49659703 | Ccr2 | 0.916336 |
| piR-mmu-49659703 | Rab21 | 0.900839 |
| piR-mmu-10940440 | 4930474N05Rik | 0.964711 |
| piR-mmu-10940440 | Hs6st2 | 0.95515 |
| piR-mmu-10940440 | 1700019D03Rik | 0.947226 |
| piR-mmu-10940440 | Esyt2 | 0.939808 |
| piR-mmu-10940440 | Rsf1 | 0.934093 |
| piR-mmu-10940440 | Rab21 | 0.934003 |
| piR-mmu-10940440 | Zfp521 | 0.930792 |
| piR-mmu-10940440 | Fst | 0.92012 |
| piR-mmu-10940440 | Gm4631 | 0.919306 |
| piR-mmu-10940440 | Ttn | 0.909108 |
| piR-mmu-10940440 | Pcca | 0.905983 |
| piR-mmu-10940440 | Slc5a4b | 0.900261 |
| piR-mmu-10960276 | 4930474N05Rik | 0.96526 |
| piR-mmu-10960276 | Hs6st2 | 0.957634 |
| piR-mmu-10960276 | Esyt2 | 0.938336 |
| piR-mmu-10960276 | Rsf1 | 0.934533 |
| piR-mmu-10960276 | Slc5a4b | 0.930956 |
| piR-mmu-10960276 | Rab21 | 0.930505 |
| piR-mmu-10960276 | Zfp521 | 0.929733 |
| piR-mmu-10960276 | Gm4631 | 0.925558 |
| piR-mmu-10960276 | Fst | 0.92186 |
| piR-mmu-10960276 | 1700019D03Rik | 0.920857 |

| | | |
|---|---|---|
| piR-mmu-10960276 | Chka | 0.914617 |
| piR-mmu-10960276 | Ttn | 0.914221 |
| piR-mmu-10911100 | 4930474N05Rik | 0.965353 |
| piR-mmu-10911100 | Hs6st2 | 0.955944 |
| piR-mmu-10911100 | Rab21 | 0.935948 |
| piR-mmu-10911100 | Rsf1 | 0.934902 |
| piR-mmu-10911100 | Zfp521 | 0.933796 |
| piR-mmu-10911100 | Esyt2 | 0.931586 |
| piR-mmu-10911100 | Gm4631 | 0.925821 |
| piR-mmu-10911100 | Chka | 0.925384 |
| piR-mmu-10911100 | Fst | 0.920628 |
| piR-mmu-10911100 | Ttn | 0.918183 |
| piR-mmu-10911100 | Slc5a4b | 0.912941 |
| piR-mmu-10911100 | 1700019D03Rik | 0.91087 |
| piR-mmu-10911100 | Gm17359 | 0.902237 |
| piR-mmu-10916134 | Rnf115 | 0.996363 |
| piR-mmu-10916134 | Gm7293 | 0.996051 |
| piR-mmu-10916134 | Fam120a | 0.98968 |
| piR-mmu-10916134 | Khdc1a | 0.986546 |
| piR-mmu-10916134 | Gria2 | 0.985582 |
| piR-mmu-10916134 | E130114P18Rik | 0.981587 |
| piR-mmu-10916134 | Dip2c | 0.974722 |
| piR-mmu-10916134 | Cd46 | 0.969067 |
| piR-mmu-10916134 | Snx7 | 0.968274 |
| piR-mmu-10916134 | Kif5b | 0.964012 |
| piR-mmu-10916134 | Parp6 | 0.963692 |
| piR-mmu-10916134 | Ssfa2 | 0.958597 |
| piR-mmu-10916134 | Ogt | 0.957271 |
| piR-mmu-10916134 | Gabrb2 | 0.954803 |
| piR-mmu-10916134 | Trim63 | 0.953541 |
| piR-mmu-10916134 | Atp2b1 | 0.952864 |
| piR-mmu-10916134 | Itgae | 0.952398 |
| piR-mmu-10916134 | Wtap | 0.951748 |
| piR-mmu-10916134 | Zfp810 | 0.94986 |
| piR-mmu-10916134 | Inpp4a | 0.949597 |
| piR-mmu-10916134 | Zeb2 | 0.945821 |
| piR-mmu-10916134 | Hmga2 | 0.941135 |
| piR-mmu-10916134 | Tanc2 | 0.934547 |
| piR-mmu-10916134 | Edn1 | 0.933666 |
| piR-mmu-10916134 | Skap1 | 0.933377 |
| piR-mmu-10916134 | Kctd8 | 0.929103 |
| piR-mmu-10916134 | Adamts5 | 0.927853 |
| piR-mmu-10916134 | Dsg4 | 0.925268 |
| piR-mmu-10916134 | Sumo2 | 0.921627 |
| piR-mmu-10916134 | Glis3 | 0.916447 |
| piR-mmu-10916134 | Zdhhc15 | 0.914163 |
| piR-mmu-10916134 | Csmd3 | 0.91243 |
| piR-mmu-10916134 | Gspt1 | 0.909657 |
| piR-mmu-10916134 | Speer3 | 0.907381 |
| piR-mmu-10916134 | Dmd | 0.904811 |
| piR-mmu-10916134 | Exoc8 | 0.904503 |
| piR-mmu-10916134 | Dennd1b | 0.904069 |
| piR-mmu-10916134 | Dclk3 | 0.900007 |

| | | |
|---|---|---|
| piR-mmu-10953882 | 4930474N05Rik | 0.964431 |
| piR-mmu-10953882 | Hs6st2 | 0.957198 |
| piR-mmu-10953882 | Rsf1 | 0.937304 |
| piR-mmu-10953882 | Slc5a4b | 0.932473 |
| piR-mmu-10953882 | Zfp521 | 0.931741 |
| piR-mmu-10953882 | Rab21 | 0.931315 |
| piR-mmu-10953882 | 1700019D03Rik | 0.930182 |
| piR-mmu-10953882 | Ttn | 0.926464 |
| piR-mmu-10953882 | Gm4631 | 0.923247 |
| piR-mmu-10953882 | Fst | 0.9226 |
| piR-mmu-10953882 | Chka | 0.913472 |
| piR-mmu-10953882 | Gm17359 | 0.90965 |
| piR-mmu-10953882 | Esyt2 | 0.905671 |
| piR-mmu-10911632 | 4930474N05Rik | 0.965849 |
| piR-mmu-10911632 | Hs6st2 | 0.956906 |
| piR-mmu-10911632 | Rsf1 | 0.933862 |
| piR-mmu-10911632 | Zfp521 | 0.931634 |
| piR-mmu-10911632 | Rab21 | 0.92832 |
| piR-mmu-10911632 | Slc5a4b | 0.926282 |
| piR-mmu-10911632 | Gm4631 | 0.922173 |
| piR-mmu-10911632 | Fst | 0.921054 |
| piR-mmu-10911632 | Ttn | 0.920185 |
| piR-mmu-10911632 | Chka | 0.913472 |
| piR-mmu-10911632 | Cpt1a | 0.903908 |
| piR-mmu-10911823 | Lrrtm3 | 0.951583 |
| piR-mmu-10911823 | Arhgap29 | 0.907845 |
| piR-mmu-31033718 | Eif4g2 | 0.999983 |
| piR-mmu-31033718 | Nfat5 | 0.998628 |
| piR-mmu-31033718 | Ranbp2 | 0.997541 |
| piR-mmu-31033718 | Samd10 | 0.986525 |
| piR-mmu-31033718 | Sgce | 0.985228 |
| piR-mmu-31033718 | Adgrb3 | 0.985088 |
| piR-mmu-31033718 | Zbtb20 | 0.983943 |
| piR-mmu-31033718 | Nova1 | 0.983798 |
| piR-mmu-31033718 | Il31 | 0.983604 |
| piR-mmu-31033718 | Arnt | 0.981902 |
| piR-mmu-31033718 | Sclt1 | 0.979788 |
| piR-mmu-31033718 | Stc1 | 0.979479 |
| piR-mmu-31033718 | Sbno1 | 0.977607 |
| piR-mmu-31033718 | Gm10797 | 0.977531 |
| piR-mmu-31033718 | Gm7293 | 0.977264 |
| piR-mmu-31033718 | D030056L22Rik | 0.976615 |
| piR-mmu-31033718 | Fopnl | 0.976602 |
| piR-mmu-31033718 | Rnf38 | 0.976505 |
| piR-mmu-31033718 | Btg1 | 0.974734 |
| piR-mmu-31033718 | Chic1 | 0.972183 |
| piR-mmu-31033718 | Rev3l | 0.970514 |
| piR-mmu-31033718 | Runx1 | 0.963399 |
| piR-mmu-31033718 | Ccdc171 | 0.962169 |
| piR-mmu-31033718 | Ube3b | 0.961208 |
| piR-mmu-31033718 | Rel | 0.961151 |
| piR-mmu-31033718 | D1Ertd622e | 0.960897 |
| piR-mmu-31033718 | Traf6 | 0.956629 |

| | | |
|---|---|---|
| piR-mmu-31033718 | Nebl | 0.9562 |
| piR-mmu-31033718 | Dgkk | 0.955118 |
| piR-mmu-31033718 | Mlf2 | 0.951315 |
| piR-mmu-31033718 | Strbp | 0.950261 |
| piR-mmu-31033718 | Ttc3 | 0.949948 |
| piR-mmu-31033718 | Hnrnpa2b1 | 0.949467 |
| piR-mmu-31033718 | Parp1 | 0.949181 |
| piR-mmu-31033718 | Armc9 | 0.948661 |
| piR-mmu-31033718 | Arhgap11a | 0.947748 |
| piR-mmu-31033718 | Rnf103 | 0.947234 |
| piR-mmu-31033718 | Ncaph | 0.944404 |
| piR-mmu-31033718 | Nlgn1 | 0.944087 |
| piR-mmu-31033718 | Sobp | 0.943185 |
| piR-mmu-31033718 | Dtna | 0.942591 |
| piR-mmu-31033718 | Bcorl1 | 0.939547 |
| piR-mmu-31033718 | Fam96a | 0.939463 |
| piR-mmu-31033718 | Cdc42bpb | 0.937959 |
| piR-mmu-31033718 | Tasp1 | 0.937842 |
| piR-mmu-31033718 | Rab30 | 0.937193 |
| piR-mmu-31033718 | Vstm2a | 0.936833 |
| piR-mmu-31033718 | Cnpy2 | 0.936791 |
| piR-mmu-31033718 | Esyt2 | 0.936334 |
| piR-mmu-31033718 | Adgrf5 | 0.93599 |
| piR-mmu-31033718 | Nprl3 | 0.935674 |
| piR-mmu-31033718 | Ccl25 | 0.934322 |
| piR-mmu-31033718 | Cnot6l | 0.932215 |
| piR-mmu-31033718 | Map1b | 0.931854 |
| piR-mmu-31033718 | Nuak1 | 0.931639 |
| piR-mmu-31033718 | Sdc2 | 0.931596 |
| piR-mmu-31033718 | Clasp1 | 0.929532 |
| piR-mmu-31033718 | Rab11a | 0.928811 |
| piR-mmu-31033718 | Megf10 | 0.928806 |
| piR-mmu-31033718 | Ubn2 | 0.928365 |
| piR-mmu-31033718 | Mri1 | 0.926605 |
| piR-mmu-31033718 | Rbm38 | 0.926191 |
| piR-mmu-31033718 | Dazap2 | 0.923821 |
| piR-mmu-31033718 | Sbspon | 0.923778 |
| piR-mmu-31033718 | Prox1 | 0.919549 |
| piR-mmu-31033718 | Zscan29 | 0.914364 |
| piR-mmu-31033718 | Elf1 | 0.913112 |
| piR-mmu-31033718 | Sub1 | 0.912637 |
| piR-mmu-31033718 | Nhsl2 | 0.911926 |
| piR-mmu-31033718 | Rps6ka3 | 0.908095 |
| piR-mmu-31033718 | Susd6 | 0.90766 |
| piR-mmu-31033718 | Sdha | 0.90428 |
| piR-mmu-31033718 | Ank2 | 0.900837 |
| piR-mmu-31033718 | Numb | 0.900165 |
| piR-mmu-49287809 | Ube2l3 | 0.989411 |
| piR-mmu-49287809 | Csmd3 | 0.987726 |
| piR-mmu-49287809 | Dgcr2 | 0.987159 |
| piR-mmu-49287809 | Arel1 | 0.982468 |
| piR-mmu-49287809 | Rnf224 | 0.975654 |
| piR-mmu-49287809 | Slc5a4b | 0.975071 |

| | | |
|---|---|---|
| piR-mmu-49287809 | Cpt1a | 0.961046 |
| piR-mmu-49287809 | Zfp521 | 0.957159 |
| piR-mmu-49287809 | Mbd2 | 0.954616 |
| piR-mmu-49287809 | Esyt2 | 0.943733 |
| piR-mmu-49287809 | Ccr2 | 0.942246 |
| piR-mmu-49287809 | Cdh4 | 0.941088 |
| piR-mmu-49287809 | Spink8 | 0.939565 |
| piR-mmu-49287809 | Chd9 | 0.93867 |
| piR-mmu-49287809 | Zfyve27 | 0.937524 |
| piR-mmu-49287809 | Gramd2 | 0.936208 |
| piR-mmu-49287809 | Abhd2 | 0.935704 |
| piR-mmu-49287809 | 4933402J07Rik | 0.925557 |
| piR-mmu-49287809 | Chka | 0.924912 |
| piR-mmu-49287809 | Tshz1 | 0.923252 |
| piR-mmu-49287809 | Mup1 | 0.917184 |
| piR-mmu-49287809 | Mup5 | 0.914278 |
| piR-mmu-49287809 | Scg5 | 0.907534 |
| piR-mmu-49287809 | Fam78b | 0.905207 |
| piR-mmu-49287809 | Tob1 | 0.900567 |
| piR-mmu-10932261 | 4930474N05Rik | 0.964431 |
| piR-mmu-10932261 | Hs6st2 | 0.954338 |
| piR-mmu-10932261 | Rab21 | 0.95064 |
| piR-mmu-10932261 | Rsf1 | 0.937182 |
| piR-mmu-10932261 | Zfp521 | 0.931571 |
| piR-mmu-10932261 | Fst | 0.930162 |
| piR-mmu-10932261 | Slc5a4b | 0.92786 |
| piR-mmu-10932261 | Ttn | 0.927576 |
| piR-mmu-10932261 | 1700019D03Rik | 0.92513 |
| piR-mmu-10932261 | Gm4631 | 0.924239 |
| piR-mmu-10932261 | Esyt2 | 0.916619 |
| piR-mmu-10932261 | Chka | 0.913472 |
| piR-mmu-10932261 | Gm17359 | 0.90965 |
| piR-mmu-3213041 | Nrep | 0.999824 |
| piR-mmu-3213041 | Brinp3 | 0.997974 |
| piR-mmu-3213041 | Rc3h2 | 0.997765 |
| piR-mmu-3213041 | Eif1 | 0.995998 |
| piR-mmu-3213041 | C1galt1 | 0.994058 |
| piR-mmu-3213041 | Hnrnpu | 0.992475 |
| piR-mmu-3213041 | 2310035C23Rik | 0.991635 |
| piR-mmu-3213041 | Hivep2 | 0.989907 |
| piR-mmu-3213041 | Atxn7 | 0.986983 |
| piR-mmu-3213041 | Cap2 | 0.986484 |
| piR-mmu-3213041 | Rqcd1 | 0.984655 |
| piR-mmu-3213041 | Mbl2 | 0.984577 |
| piR-mmu-3213041 | Eif4a2 | 0.9739 |
| piR-mmu-3213041 | Nrn1 | 0.9737 |
| piR-mmu-3213041 | Pank3 | 0.973451 |
| piR-mmu-3213041 | Pou3f2 | 0.972184 |
| piR-mmu-3213041 | Rbm41 | 0.971685 |
| piR-mmu-3213041 | Tpp2 | 0.97112 |
| piR-mmu-3213041 | Cwc15 | 0.970411 |
| piR-mmu-3213041 | Shox2 | 0.967567 |
| piR-mmu-3213041 | Vps54 | 0.965507 |

| | | |
|---|---|---|
| piR-mmu-3213041 | Zfp617 | 0.965451 |
| piR-mmu-3213041 | Arntl | 0.963779 |
| piR-mmu-3213041 | Cers6 | 0.961739 |
| piR-mmu-3213041 | Zfp420 | 0.961403 |
| piR-mmu-3213041 | Rgag4 | 0.960231 |
| piR-mmu-3213041 | Dnajc3 | 0.959739 |
| piR-mmu-3213041 | Hrnr | 0.953537 |
| piR-mmu-3213041 | Zfp811 | 0.950772 |
| piR-mmu-3213041 | Chd8 | 0.949925 |
| piR-mmu-3213041 | A130051J06Rik | 0.948269 |
| piR-mmu-3213041 | Ythdc1 | 0.948185 |
| piR-mmu-3213041 | Rap1b | 0.947775 |
| piR-mmu-3213041 | Ssr3 | 0.946436 |
| piR-mmu-3213041 | Myo10 | 0.946047 |
| piR-mmu-3213041 | Nmral1 | 0.945742 |
| piR-mmu-3213041 | Zc3h11a | 0.944826 |
| piR-mmu-3213041 | Kdm4c | 0.943499 |
| piR-mmu-3213041 | Mgat5 | 0.94316 |
| piR-mmu-3213041 | B020004C17Rik | 0.937373 |
| piR-mmu-3213041 | Amot | 0.936437 |
| piR-mmu-3213041 | Car13 | 0.935888 |
| piR-mmu-3213041 | Ppp1r14c | 0.935353 |
| piR-mmu-3213041 | Pgm3 | 0.93467 |
| piR-mmu-3213041 | Dcaf12l1 | 0.931208 |
| piR-mmu-3213041 | Wbp5 | 0.929883 |
| piR-mmu-3213041 | Endod1 | 0.929479 |
| piR-mmu-3213041 | Meis2 | 0.924186 |
| piR-mmu-3213041 | Piezo2 | 0.922288 |
| piR-mmu-3213041 | Cdk14 | 0.917968 |
| piR-mmu-3213041 | Rbm8a2 | 0.917858 |
| piR-mmu-3213041 | Gad2 | 0.906728 |
| piR-mmu-3213041 | Pum3 | 0.906719 |
| piR-mmu-3213041 | Arhgap44 | 0.906377 |
| piR-mmu-3213041 | Mob3b | 0.905532 |
| piR-mmu-3213041 | Esrrg | 0.901567 |
| piR-mmu-23832900 | Stx8 | 0.995826 |
| piR-mmu-23832900 | Mr1 | 0.981602 |
| piR-mmu-23832900 | Fign | 0.978314 |
| piR-mmu-23832900 | Zranb2 | 0.976484 |
| piR-mmu-23832900 | Mob3b | 0.973341 |
| piR-mmu-23832900 | Usp25 | 0.970149 |
| piR-mmu-23832900 | Nemf | 0.966023 |
| piR-mmu-23832900 | Lrrc7 | 0.959031 |
| piR-mmu-23832900 | Aak1 | 0.957994 |
| piR-mmu-23832900 | Ypel5 | 0.957395 |
| piR-mmu-23832900 | Gpr22 | 0.955842 |
| piR-mmu-23832900 | RP23-281H4.10 | 0.9468 |
| piR-mmu-23832900 | Dnajc3 | 0.939652 |
| piR-mmu-23832900 | Spag9 | 0.933865 |
| piR-mmu-23832900 | Clhc1 | 0.927338 |
| piR-mmu-23832900 | Fam69a | 0.915759 |
| piR-mmu-23832900 | Stap1 | 0.914869 |
| piR-mmu-23832900 | Gm94 | 0.910418 |

| | | |
|---|---|---|
| piR-mmu-23832900 | Dnajc19 | 0.910236 |
| piR-mmu-23832900 | Rmnd5a | 0.907867 |
| piR-mmu-23832900 | Hmga2 | 0.906575 |
| piR-mmu-23832900 | Zfp280d | 0.904641 |
| piR-mmu-23832900 | Tnpo3 | 0.904362 |
| piR-mmu-23832900 | Trim44 | 0.901273 |
| piR-mmu-49015749 | Xlr5c | 0.999998 |
| piR-mmu-49015749 | Xlr5b | 0.999997 |
| piR-mmu-49015749 | Xlr5a | 0.999984 |
| piR-mmu-49015749 | Gm9936 | 0.999679 |
| piR-mmu-49015749 | Celf2 | 0.999667 |
| piR-mmu-49015749 | Gstk1 | 0.998242 |
| piR-mmu-49015749 | Pcdh20 | 0.996821 |
| piR-mmu-49015749 | Pdzd4 | 0.996326 |
| piR-mmu-49015749 | Hectd2 | 0.995472 |
| piR-mmu-49015749 | Cyp20a1 | 0.994803 |
| piR-mmu-49015749 | Dok4 | 0.994478 |
| piR-mmu-49015749 | Trub2 | 0.994428 |
| piR-mmu-49015749 | Ip6k2 | 0.994301 |
| piR-mmu-49015749 | Sh3pxd2a | 0.99427 |
| piR-mmu-49015749 | Dusp12 | 0.993525 |
| piR-mmu-49015749 | Unc5c | 0.993355 |
| piR-mmu-49015749 | Vstm2b | 0.99189 |
| piR-mmu-49015749 | Srek1 | 0.989659 |
| piR-mmu-49015749 | Lmx1a | 0.98958 |
| piR-mmu-49015749 | Ddx3y | 0.989389 |
| piR-mmu-49015749 | Gm8882 | 0.987769 |
| piR-mmu-49015749 | Kbtbd2 | 0.986599 |
| piR-mmu-49015749 | Trmt12 | 0.985482 |
| piR-mmu-49015749 | Clcn1 | 0.985459 |
| piR-mmu-49015749 | Vegfb | 0.985359 |
| piR-mmu-49015749 | Ccbe1 | 0.985292 |
| piR-mmu-49015749 | Tmem67 | 0.985235 |
| piR-mmu-49015749 | Zdhhc15 | 0.984047 |
| piR-mmu-49015749 | Tmprss12 | 0.984014 |
| piR-mmu-49015749 | Rsl1d1 | 0.981891 |
| piR-mmu-49015749 | Ano2 | 0.981839 |
| piR-mmu-49015749 | Wdr38 | 0.981603 |
| piR-mmu-49015749 | Cdk13 | 0.979506 |
| piR-mmu-49015749 | Chst10 | 0.977207 |
| piR-mmu-49015749 | Wnt7a | 0.975967 |
| piR-mmu-49015749 | Ywhab | 0.97574 |
| piR-mmu-49015749 | Senp1 | 0.973878 |
| piR-mmu-49015749 | Tifab | 0.971379 |
| piR-mmu-49015749 | Ppp4c | 0.970313 |
| piR-mmu-49015749 | Boc | 0.966354 |
| piR-mmu-49015749 | Chek1 | 0.966223 |
| piR-mmu-49015749 | Pde6d | 0.965612 |
| piR-mmu-49015749 | Cnfn | 0.965359 |
| piR-mmu-49015749 | Dmtf1 | 0.963632 |
| piR-mmu-49015749 | Anxa7 | 0.963413 |
| piR-mmu-49015749 | Ssbp1 | 0.962644 |
| piR-mmu-49015749 | 4930503B20Rik | 0.962518 |

| | | |
|---|---|---|
| piR-mmu-49015749 | Zbtb20 | 0.960432 |
| piR-mmu-49015749 | Mtf1 | 0.959222 |
| piR-mmu-49015749 | Cxxc4 | 0.958428 |
| piR-mmu-49015749 | Btbd1 | 0.957483 |
| piR-mmu-49015749 | Dhrs3 | 0.957226 |
| piR-mmu-49015749 | Lingo1 | 0.956284 |
| piR-mmu-49015749 | Ddx5 | 0.955887 |
| piR-mmu-49015749 | Rabl3 | 0.955657 |
| piR-mmu-49015749 | Pak3 | 0.954058 |
| piR-mmu-49015749 | D1Ertd622e | 0.95339 |
| piR-mmu-49015749 | Tead1 | 0.952824 |
| piR-mmu-49015749 | Clmn | 0.952562 |
| piR-mmu-49015749 | Fbxl21 | 0.950592 |
| piR-mmu-49015749 | Ros1 | 0.948092 |
| piR-mmu-49015749 | Tbc1d19 | 0.947741 |
| piR-mmu-49015749 | Tsr2 | 0.947255 |
| piR-mmu-49015749 | Dsg1a | 0.94447 |
| piR-mmu-49015749 | Arhgef10l | 0.94274 |
| piR-mmu-49015749 | Onecut2 | 0.942323 |
| piR-mmu-49015749 | 9930022D16Rik | 0.942021 |
| piR-mmu-49015749 | Vwc2 | 0.939118 |
| piR-mmu-49015749 | Rgs20 | 0.938553 |
| piR-mmu-49015749 | 2010005H15Rik | 0.938248 |
| piR-mmu-49015749 | Slc39a2 | 0.938085 |
| piR-mmu-49015749 | Cnot4 | 0.937156 |
| piR-mmu-49015749 | Hyi | 0.936801 |
| piR-mmu-49015749 | Pak1 | 0.934075 |
| piR-mmu-49015749 | Lin9 | 0.933368 |
| piR-mmu-49015749 | Prdm9 | 0.932903 |
| piR-mmu-49015749 | Pank2 | 0.930444 |
| piR-mmu-49015749 | Gm5591 | 0.929384 |
| piR-mmu-49015749 | Gm28551 | 0.928088 |
| piR-mmu-49015749 | Vma21 | 0.927715 |
| piR-mmu-49015749 | Efna5 | 0.927302 |
| piR-mmu-49015749 | Gtf2h1 | 0.926903 |
| piR-mmu-49015749 | Usp7 | 0.926718 |
| piR-mmu-49015749 | Arhgdig | 0.926497 |
| piR-mmu-49015749 | Fam117b | 0.926184 |
| piR-mmu-49015749 | Armc1 | 0.925886 |
| piR-mmu-49015749 | Taf12 | 0.924985 |
| piR-mmu-49015749 | Rad54l2 | 0.924556 |
| piR-mmu-49015749 | Eda | 0.923833 |
| piR-mmu-49015749 | Rbm46 | 0.922315 |
| piR-mmu-49015749 | Col6a2 | 0.921781 |
| piR-mmu-49015749 | Daam1 | 0.921045 |
| piR-mmu-49015749 | Urgcp | 0.919147 |
| piR-mmu-49015749 | Ebf4 | 0.918959 |
| piR-mmu-49015749 | Myo7a | 0.916905 |
| piR-mmu-49015749 | Onecut3 | 0.916448 |
| piR-mmu-49015749 | Clcf1 | 0.916353 |
| piR-mmu-49015749 | Prkce | 0.915759 |
| piR-mmu-49015749 | Elf4 | 0.915326 |
| piR-mmu-49015749 | A730049H05Rik | 0.91494 |

| | | |
|---|---|---|
| piR-mmu-49015749 | 4933434E20Rik | 0.914583 |
| piR-mmu-49015749 | Trpv3 | 0.914126 |
| piR-mmu-49015749 | Lyn | 0.914034 |
| piR-mmu-49015749 | Defb1 | 0.913777 |
| piR-mmu-49015749 | March9 | 0.91223 |
| piR-mmu-49015749 | Rab43 | 0.911346 |
| piR-mmu-49015749 | Cyp2c37 | 0.91098 |
| piR-mmu-49015749 | Cyp2c54 | 0.910637 |
| piR-mmu-49015749 | Fign | 0.908119 |
| piR-mmu-49015749 | Elavl4 | 0.907551 |
| piR-mmu-49015749 | Cluh | 0.907456 |
| piR-mmu-49015749 | Ddhd2 | 0.907134 |
| piR-mmu-49015749 | Slc12a2 | 0.906476 |
| piR-mmu-49015749 | Xrcc2 | 0.903051 |
| piR-mmu-49015749 | Smek1 | 0.902697 |
| piR-mmu-49015749 | Crocc2 | 0.902003 |
| piR-mmu-49015749 | Fhl1 | 0.901909 |
| piR-mmu-49015749 | BC024978 | 0.901532 |
| piR-mmu-685550 | Nrn1 | 0.999974 |
| piR-mmu-685550 | Zfp184 | 0.999951 |
| piR-mmu-685550 | Ptpn12 | 0.999871 |
| piR-mmu-685550 | Tmed9 | 0.999832 |
| piR-mmu-685550 | Ptbp2 | 0.999416 |
| piR-mmu-685550 | Gm26965 | 0.999285 |
| piR-mmu-685550 | Cdk17 | 0.998867 |
| piR-mmu-685550 | Ikzf2 | 0.998329 |
| piR-mmu-685550 | Tshz3 | 0.998251 |
| piR-mmu-685550 | Tmem170b | 0.998226 |
| piR-mmu-685550 | Ogt | 0.997791 |
| piR-mmu-685550 | Fbrs | 0.997742 |
| piR-mmu-685550 | Gm28168 | 0.997142 |
| piR-mmu-685550 | Rap1b | 0.99231 |
| piR-mmu-685550 | Pcf11 | 0.991979 |
| piR-mmu-685550 | Xpot | 0.991316 |
| piR-mmu-685550 | Zfp735 | 0.991064 |
| piR-mmu-685550 | Zfp9 | 0.990147 |
| piR-mmu-685550 | Slco6d1 | 0.989546 |
| piR-mmu-685550 | Gbp7 | 0.988374 |
| piR-mmu-685550 | Frrs1 | 0.987066 |
| piR-mmu-685550 | Zfp426 | 0.987016 |
| piR-mmu-685550 | Agbl1 | 0.986217 |
| piR-mmu-685550 | Ogdh | 0.986034 |
| piR-mmu-685550 | Fbxo27 | 0.984537 |
| piR-mmu-685550 | Zfp846 | 0.984494 |
| piR-mmu-685550 | Zfp182 | 0.981111 |
| piR-mmu-685550 | Zfp266 | 0.980412 |
| piR-mmu-685550 | Gatsl2 | 0.979822 |
| piR-mmu-685550 | Zfp383 | 0.97922 |
| piR-mmu-685550 | 2410141K09Rik | 0.978207 |
| piR-mmu-685550 | Zfp59 | 0.978189 |
| piR-mmu-685550 | Gm26920 | 0.978067 |
| piR-mmu-685550 | Ugt2b5 | 0.976711 |
| piR-mmu-685550 | Zfp709 | 0.976484 |

| | | |
|---|---|---|
| piR-mmu-685550 | Dnajc3 | 0.976483 |
| piR-mmu-685550 | Arf3 | 0.975875 |
| piR-mmu-685550 | Zfp641 | 0.975519 |
| piR-mmu-685550 | Fgd5 | 0.974558 |
| piR-mmu-685550 | Phrf1 | 0.972361 |
| piR-mmu-685550 | Cdk16 | 0.970689 |
| piR-mmu-685550 | Zfp715 | 0.970308 |
| piR-mmu-685550 | Zfp617 | 0.969576 |
| piR-mmu-685550 | Pde7a | 0.969172 |
| piR-mmu-685550 | Hmgxb4 | 0.968669 |
| piR-mmu-685550 | Zfp24 | 0.968156 |
| piR-mmu-685550 | Huwe1 | 0.967804 |
| piR-mmu-685550 | Zfp472 | 0.966204 |
| piR-mmu-685550 | Zfp60 | 0.966047 |
| piR-mmu-685550 | Nufip2 | 0.965909 |
| piR-mmu-685550 | Zfp850 | 0.965801 |
| piR-mmu-685550 | Tle3 | 0.965665 |
| piR-mmu-685550 | Zfp286 | 0.965219 |
| piR-mmu-685550 | Zfp568 | 0.963597 |
| piR-mmu-685550 | Zfp626 | 0.963259 |
| piR-mmu-685550 | B020011L13Rik | 0.962373 |
| piR-mmu-685550 | Zfp81 | 0.962266 |
| piR-mmu-685550 | 1700049G17Rik | 0.961892 |
| piR-mmu-685550 | Zfp763 | 0.961322 |
| piR-mmu-685550 | Nhp2 | 0.959631 |
| piR-mmu-685550 | Zik1 | 0.959543 |
| piR-mmu-685550 | Zfp563 | 0.959131 |
| piR-mmu-685550 | Mtap | 0.959122 |
| piR-mmu-685550 | Csnk1g1 | 0.959039 |
| piR-mmu-685550 | Zfp961 | 0.959 |
| piR-mmu-685550 | C030039L03Rik | 0.958202 |
| piR-mmu-685550 | Zfp599 | 0.957301 |
| piR-mmu-685550 | Zfp418 | 0.957282 |
| piR-mmu-685550 | Tra2a | 0.957037 |
| piR-mmu-685550 | 4930503E14Rik | 0.956889 |
| piR-mmu-685550 | Macrod2 | 0.956621 |
| piR-mmu-685550 | Surf4 | 0.956436 |
| piR-mmu-685550 | Zfp58 | 0.956218 |
| piR-mmu-685550 | Zfp146 | 0.956025 |
| piR-mmu-685550 | Rbak | 0.955242 |
| piR-mmu-685550 | Zfp85 | 0.955211 |
| piR-mmu-685550 | Mblac2 | 0.955033 |
| piR-mmu-685550 | Bcl2l2 | 0.9547 |
| piR-mmu-685550 | Ammecr1l | 0.95397 |
| piR-mmu-685550 | Fbxo42 | 0.95314 |
| piR-mmu-685550 | Ythdf3 | 0.953113 |
| piR-mmu-685550 | Zfp397 | 0.952051 |
| piR-mmu-685550 | Fam188a | 0.949787 |
| piR-mmu-685550 | Zfp260 | 0.949699 |
| piR-mmu-685550 | Peg3 | 0.949469 |
| piR-mmu-685550 | Zfp37 | 0.94844 |
| piR-mmu-685550 | Parg | 0.948129 |
| piR-mmu-685550 | Stx6 | 0.948094 |

| | | |
|---|---|---|
| piR-mmu-685550 | Zfp658 | 0.947979 |
| piR-mmu-685550 | Zfp26 | 0.947934 |
| piR-mmu-685550 | Fut10 | 0.94742 |
| piR-mmu-685550 | Rnf14 | 0.947027 |
| piR-mmu-685550 | Lypla1 | 0.944872 |
| piR-mmu-685550 | Zfp74 | 0.944674 |
| piR-mmu-685550 | Zfp62 | 0.9438 |
| piR-mmu-685550 | Lmnb1 | 0.943718 |
| piR-mmu-685550 | Zfp882 | 0.943279 |
| piR-mmu-685550 | Zfp949 | 0.942077 |
| piR-mmu-685550 | Zfp607 | 0.941957 |
| piR-mmu-685550 | Zfp799 | 0.941607 |
| piR-mmu-685550 | Mbp | 0.941059 |
| piR-mmu-685550 | Scn4b | 0.939628 |
| piR-mmu-685550 | Zfp354a | 0.938643 |
| piR-mmu-685550 | Zfp874a | 0.938247 |
| piR-mmu-685550 | Phactr2 | 0.937508 |
| piR-mmu-685550 | Arpc4 | 0.936762 |
| piR-mmu-685550 | Zfp35 | 0.935222 |
| piR-mmu-685550 | Zfp87 | 0.934333 |
| piR-mmu-685550 | Snx2 | 0.934012 |
| piR-mmu-685550 | Zfp872 | 0.931607 |
| piR-mmu-685550 | Zkscan16 | 0.931099 |
| piR-mmu-685550 | Arid2 | 0.930836 |
| piR-mmu-685550 | Zfp449 | 0.930369 |
| piR-mmu-685550 | Abi1 | 0.930264 |
| piR-mmu-685550 | Zfp874b | 0.929264 |
| piR-mmu-685550 | Rnf182 | 0.92893 |
| piR-mmu-685550 | Zkscan8 | 0.927506 |
| piR-mmu-685550 | Zfp189 | 0.927008 |
| piR-mmu-685550 | Zfp667 | 0.926676 |
| piR-mmu-685550 | Arfgef2 | 0.926356 |
| piR-mmu-685550 | Zfp560 | 0.926353 |
| piR-mmu-685550 | Rock1 | 0.924665 |
| piR-mmu-685550 | Gm21987 | 0.924514 |
| piR-mmu-685550 | Actr10 | 0.924168 |
| piR-mmu-685550 | Cpsf7 | 0.92341 |
| piR-mmu-685550 | Zfp473 | 0.921306 |
| piR-mmu-685550 | Mark4 | 0.921006 |
| piR-mmu-685550 | Tmem174 | 0.920988 |
| piR-mmu-685550 | Slfn3 | 0.919952 |
| piR-mmu-685550 | Rabl6 | 0.919287 |
| piR-mmu-685550 | Gm28557 | 0.91855 |
| piR-mmu-685550 | Ccdc30 | 0.917939 |
| piR-mmu-685550 | Zfp719 | 0.917633 |
| piR-mmu-685550 | Ubxn7 | 0.916807 |
| piR-mmu-685550 | Sirt1 | 0.915923 |
| piR-mmu-685550 | Zfp180 | 0.915762 |
| piR-mmu-685550 | Tlr1 | 0.914238 |
| piR-mmu-685550 | Icosl | 0.913195 |
| piR-mmu-685550 | Tardbp | 0.911282 |
| piR-mmu-685550 | Adamts16 | 0.911282 |
| piR-mmu-685550 | R3hcc1l | 0.910499 |

| | | |
|---|---|---|
| piR-mmu-685550 | Wfikkn2 | 0.910438 |
| piR-mmu-685550 | Esco2 | 0.909201 |
| piR-mmu-685550 | Zfp248 | 0.909142 |
| piR-mmu-685550 | Tor1b | 0.908568 |
| piR-mmu-685550 | Ran | 0.907506 |
| piR-mmu-685550 | Klra9 | 0.906847 |
| piR-mmu-685550 | Cd2bp2 | 0.906558 |
| piR-mmu-685550 | Tec | 0.905314 |
| piR-mmu-685550 | Zfp583 | 0.904502 |
| piR-mmu-685550 | Whsc1l1 | 0.904201 |
| piR-mmu-685550 | Gm7145 | 0.903494 |
| piR-mmu-685550 | Corin | 0.90309 |
| piR-mmu-685550 | Mtmr4 | 0.902469 |
| piR-mmu-685550 | Ube3c | 0.901877 |
| piR-mmu-685550 | Wbp11 | 0.901451 |
| piR-mmu-48831092 | Ip6k2 | 0.999961 |
| piR-mmu-48831092 | Marcks | 0.999866 |
| piR-mmu-48831092 | Gm9936 | 0.99956 |
| piR-mmu-48831092 | Bbx | 0.998947 |
| piR-mmu-48831092 | Xaf1 | 0.998325 |
| piR-mmu-48831092 | Stx5a | 0.996159 |
| piR-mmu-48831092 | Atp5c1 | 0.995532 |
| piR-mmu-48831092 | Gsx2 | 0.995462 |
| piR-mmu-48831092 | Dab1 | 0.995455 |
| piR-mmu-48831092 | Tnfrsf22 | 0.994769 |
| piR-mmu-48831092 | Per1 | 0.987355 |
| piR-mmu-48831092 | Kcnd3 | 0.986965 |
| piR-mmu-48831092 | Pcmtd2 | 0.985861 |
| piR-mmu-48831092 | Trp53inp1 | 0.983198 |
| piR-mmu-48831092 | Nol4l | 0.98154 |
| piR-mmu-48831092 | Veph1 | 0.981494 |
| piR-mmu-48831092 | Ctage5 | 0.981398 |
| piR-mmu-48831092 | Rbm24 | 0.980414 |
| piR-mmu-48831092 | Mier3 | 0.979343 |
| piR-mmu-48831092 | Sav1 | 0.977135 |
| piR-mmu-48831092 | Nelfa | 0.973619 |
| piR-mmu-48831092 | Bahcc1 | 0.968742 |
| piR-mmu-48831092 | Nlgn1 | 0.968283 |
| piR-mmu-48831092 | Atg7 | 0.96757 |
| piR-mmu-48831092 | Jph2 | 0.9621 |
| piR-mmu-48831092 | Col16a1 | 0.961097 |
| piR-mmu-48831092 | Gm10392 | 0.960514 |
| piR-mmu-48831092 | Gm4841 | 0.958079 |
| piR-mmu-48831092 | Cbln2 | 0.954644 |
| piR-mmu-48831092 | Duxbl3 | 0.954178 |
| piR-mmu-48831092 | Duxbl2 | 0.954178 |
| piR-mmu-48831092 | Il31ra | 0.951713 |
| piR-mmu-48831092 | Duxbl1 | 0.95056 |
| piR-mmu-48831092 | Snx4 | 0.949888 |
| piR-mmu-48831092 | Lgi2 | 0.949196 |
| piR-mmu-48831092 | Gramd1b | 0.946203 |
| piR-mmu-48831092 | Apba1 | 0.943418 |
| piR-mmu-48831092 | Mga | 0.943307 |

| | | |
|---|---|---|
| piR-mmu-48831092 | Phf21a | 0.943263 |
| piR-mmu-48831092 | Dld | 0.942564 |
| piR-mmu-48831092 | Ncoa2 | 0.941732 |
| piR-mmu-48831092 | Gps1 | 0.940407 |
| piR-mmu-48831092 | Kcnj6 | 0.938484 |
| piR-mmu-48831092 | Msi1 | 0.938093 |
| piR-mmu-48831092 | 4930568D16Rik | 0.937852 |
| piR-mmu-48831092 | Prpf40a | 0.93118 |
| piR-mmu-48831092 | Pak7 | 0.929511 |
| piR-mmu-48831092 | Smoc2 | 0.926721 |
| piR-mmu-48831092 | Kcnb1 | 0.926308 |
| piR-mmu-48831092 | Fam160b2 | 0.925926 |
| piR-mmu-48831092 | Samd4b | 0.920697 |
| piR-mmu-48831092 | Ifitm10 | 0.920633 |
| piR-mmu-48831092 | Creb1 | 0.915624 |
| piR-mmu-48831092 | Kcnip2 | 0.915453 |
| piR-mmu-48831092 | Olfr862 | 0.914635 |
| piR-mmu-48831092 | Rcn2 | 0.914619 |
| piR-mmu-48831092 | A830005F24Rik | 0.914359 |
| piR-mmu-48831092 | A330050F15Rik | 0.911595 |
| piR-mmu-48831092 | Ptch1 | 0.909213 |
| piR-mmu-48831092 | Atxn1 | 0.909033 |
| piR-mmu-48831092 | Styk1 | 0.906671 |
| piR-mmu-48831092 | Etf1 | 0.905253 |
| piR-mmu-48831092 | Cpne1 | 0.904645 |
| piR-mmu-48831092 | Ldoc1l | 0.904149 |
| piR-mmu-48831092 | Hoxc9 | 0.904105 |
| piR-mmu-48831092 | Ralgps2 | 0.902615 |
| piR-mmu-48831092 | Cilp | 0.900998 |
| piR-mmu-11461037 | 4930474N05Rik | 0.96526 |
| piR-mmu-11461037 | Hs6st2 | 0.961067 |
| piR-mmu-11461037 | Rsf1 | 0.934684 |
| piR-mmu-11461037 | 1700019D03Rik | 0.933436 |
| piR-mmu-11461037 | Esyt2 | 0.931364 |
| piR-mmu-11461037 | Zfp521 | 0.931069 |
| piR-mmu-11461037 | Rab21 | 0.929283 |
| piR-mmu-11461037 | Gm4631 | 0.924955 |
| piR-mmu-11461037 | Fst | 0.922547 |
| piR-mmu-11461037 | Ttn | 0.921627 |
| piR-mmu-11461037 | Gm17359 | 0.906316 |
| piR-mmu-240378 | Ip6k2 | 0.999927 |
| piR-mmu-240378 | Marcks | 0.999908 |
| piR-mmu-240378 | Dab1 | 0.997894 |
| piR-mmu-240378 | Bbx | 0.997554 |
| piR-mmu-240378 | Gm9936 | 0.996785 |
| piR-mmu-240378 | Stx5a | 0.996697 |
| piR-mmu-240378 | Atp5c1 | 0.995532 |
| piR-mmu-240378 | Per1 | 0.993562 |
| piR-mmu-240378 | Veph1 | 0.991206 |
| piR-mmu-240378 | Xaf1 | 0.991124 |
| piR-mmu-240378 | Kcnd3 | 0.990468 |
| piR-mmu-240378 | Gsx2 | 0.988976 |
| piR-mmu-240378 | Trp53inp1 | 0.988612 |

| | | |
|---|---|---|
| piR-mmu-240378 | Nelfa | 0.988047 |
| piR-mmu-240378 | Pcmtd2 | 0.987037 |
| piR-mmu-240378 | Jph2 | 0.983869 |
| piR-mmu-240378 | Atg7 | 0.983314 |
| piR-mmu-240378 | Ctage5 | 0.983122 |
| piR-mmu-240378 | Sav1 | 0.982927 |
| piR-mmu-240378 | Rbm24 | 0.982915 |
| piR-mmu-240378 | Gm4841 | 0.981851 |
| piR-mmu-240378 | Kcnj6 | 0.981205 |
| piR-mmu-240378 | 4930568D16Rik | 0.975124 |
| piR-mmu-240378 | Duxbl3 | 0.97034 |
| piR-mmu-240378 | Duxbl2 | 0.97034 |
| piR-mmu-240378 | Nlgn1 | 0.968658 |
| piR-mmu-240378 | Duxbl1 | 0.967926 |
| piR-mmu-240378 | Olfr862 | 0.967071 |
| piR-mmu-240378 | Il31ra | 0.963753 |
| piR-mmu-240378 | Snx4 | 0.956591 |
| piR-mmu-240378 | Gm10392 | 0.953676 |
| piR-mmu-240378 | Mga | 0.95271 |
| piR-mmu-240378 | Msi1 | 0.951681 |
| piR-mmu-240378 | Lgi2 | 0.949876 |
| piR-mmu-240378 | Kcnb1 | 0.949469 |
| piR-mmu-240378 | Phf21a | 0.949146 |
| piR-mmu-240378 | Apba1 | 0.946501 |
| piR-mmu-240378 | Nol4l | 0.946399 |
| piR-mmu-240378 | Ifitm10 | 0.943621 |
| piR-mmu-240378 | Dld | 0.941421 |
| piR-mmu-240378 | Col16a1 | 0.940893 |
| piR-mmu-240378 | Bahcc1 | 0.936708 |
| piR-mmu-240378 | A330050F15Rik | 0.935409 |
| piR-mmu-240378 | Gramd1b | 0.932119 |
| piR-mmu-240378 | Cpne1 | 0.930997 |
| piR-mmu-240378 | Ipcef1 | 0.930598 |
| piR-mmu-240378 | Smoc2 | 0.930494 |
| piR-mmu-240378 | Cacul1 | 0.929571 |
| piR-mmu-240378 | Etf1 | 0.926133 |
| piR-mmu-240378 | Ralgps2 | 0.924875 |
| piR-mmu-240378 | Otud7b | 0.923934 |
| piR-mmu-240378 | A830005F24Rik | 0.923511 |
| piR-mmu-240378 | Hoxa1 | 0.921732 |
| piR-mmu-240378 | Creb1 | 0.921214 |
| piR-mmu-240378 | Styk1 | 0.920095 |
| piR-mmu-240378 | Hcrtr2 | 0.919976 |
| piR-mmu-240378 | Gps2 | 0.91816 |
| piR-mmu-240378 | Samd4b | 0.918087 |
| piR-mmu-240378 | Mef2d | 0.91177 |
| piR-mmu-240378 | Krt71 | 0.911478 |
| piR-mmu-240378 | Rcn2 | 0.911351 |
| piR-mmu-240378 | Rnf34 | 0.910822 |
| piR-mmu-240378 | Rnf219 | 0.909843 |
| piR-mmu-240378 | Hoxc9 | 0.909716 |
| piR-mmu-240378 | Gbe1 | 0.908705 |
| piR-mmu-240378 | Cilp | 0.90861 |

| | | |
|---|---|---|
| piR-mmu-240378 | Tnfrsf22 | 0.90797 |
| piR-mmu-240378 | Dmd | 0.905676 |
| piR-mmu-240378 | Mier3 | 0.904379 |
| piR-mmu-240378 | Sp4 | 0.903745 |
| piR-mmu-240378 | Ces1h | 0.902556 |
| piR-mmu-240378 | Slc4a3 | 0.902406 |
| piR-mmu-240378 | Tmem8b | 0.901722 |
| piR-mmu-240378 | Atxn1 | 0.900782 |
| piR-mmu-33199875 | Ddx3x | 0.990283 |
| piR-mmu-33199875 | Ogt | 0.983613 |
| piR-mmu-33199875 | Pcdhb9 | 0.962229 |
| piR-mmu-33199875 | Gm15140 | 0.95953 |
| piR-mmu-33199875 | Dennd5b | 0.95787 |
| piR-mmu-33199875 | Etnk1 | 0.957412 |
| piR-mmu-33199875 | Gclm | 0.957303 |
| piR-mmu-33199875 | Nfat5 | 0.956272 |
| piR-mmu-33199875 | Igf1r | 0.946534 |
| piR-mmu-33199875 | Rita1 | 0.944728 |
| piR-mmu-33199875 | Tmem251 | 0.94211 |
| piR-mmu-33199875 | Myh3 | 0.94207 |
| piR-mmu-33199875 | Zak | 0.938356 |
| piR-mmu-33199875 | Anapc11 | 0.937379 |
| piR-mmu-33199875 | Mxi1 | 0.937326 |
| piR-mmu-33199875 | Zfand3 | 0.936705 |
| piR-mmu-33199875 | Ptprd | 0.934847 |
| piR-mmu-33199875 | Ldah | 0.934602 |
| piR-mmu-33199875 | Rgs17 | 0.931481 |
| piR-mmu-33199875 | Hibadh | 0.931175 |
| piR-mmu-33199875 | Ctdspl2 | 0.93025 |
| piR-mmu-33199875 | Onecut1 | 0.929966 |
| piR-mmu-33199875 | Plcz1 | 0.925557 |
| piR-mmu-33199875 | Meis1 | 0.921529 |
| piR-mmu-33199875 | D1Ertd622e | 0.920765 |
| piR-mmu-33199875 | Trim36 | 0.919538 |
| piR-mmu-33199875 | Fam134a | 0.917391 |
| piR-mmu-33199875 | Anks4b | 0.912455 |
| piR-mmu-33199875 | Lcp2 | 0.91112 |
| piR-mmu-33199875 | Rnf217 | 0.910388 |
| piR-mmu-33199875 | Unc5d | 0.906644 |
| piR-mmu-33199875 | Zbtb20 | 0.906533 |
| piR-mmu-33199875 | Atp1a2 | 0.904224 |
| piR-mmu-33199875 | Strap | 0.903358 |
| piR-mmu-10906591 | Gm7293 | 0.99538 |
| piR-mmu-10906591 | E130114P18Rik | 0.97511 |
| piR-mmu-10906591 | Fam120a | 0.964375 |
| piR-mmu-10906591 | Gabrb2 | 0.953747 |
| piR-mmu-10906591 | Jph1 | 0.951016 |
| piR-mmu-10906591 | Parp6 | 0.941437 |
| piR-mmu-10906591 | Snx7 | 0.931536 |
| piR-mmu-10906591 | Skap1 | 0.928247 |
| piR-mmu-10906591 | Trpm8 | 0.927265 |
| piR-mmu-10906591 | Gspt1 | 0.924726 |
| piR-mmu-10906591 | Ptprb | 0.922519 |

| | | |
|---|---|---|
| piR-mmu-10906591 | Gria2 | 0.917028 |
| piR-mmu-10906591 | Csmd3 | 0.907886 |
| piR-mmu-10906591 | Ncaph2 | 0.905987 |
| piR-mmu-10906591 | Speer3 | 0.905922 |
| piR-mmu-10906591 | Cadm2 | 0.901818 |
| piR-mmu-50207618 | 4930474N05Rik | 0.96526 |
| piR-mmu-50207618 | Hs6st2 | 0.957563 |
| piR-mmu-50207618 | Rsf1 | 0.934684 |
| piR-mmu-50207618 | Esyt2 | 0.931364 |
| piR-mmu-50207618 | Zfp521 | 0.931319 |
| piR-mmu-50207618 | Rab21 | 0.929283 |
| piR-mmu-50207618 | Slc5a4b | 0.926282 |
| piR-mmu-50207618 | Gm4631 | 0.925222 |
| piR-mmu-50207618 | Fst | 0.922547 |
| piR-mmu-50207618 | 1700019D03Rik | 0.919696 |
| piR-mmu-50207618 | Ttn | 0.919118 |
| piR-mmu-50207618 | Chka | 0.913588 |
| piR-mmu-50207618 | Gm17359 | 0.904966 |

The piRNA target gene prediction was performed with MR-microT (aggregated score $\geq$ 0.9).

**Supplementary Table S5b: Bioinformatics prediction target genes of skin-specific differentially expressed piRNAs**

| OFFICIAL_GENE_SYMBOL | Name | Species |
|---|---|---|
| Mfsd12 | major facilitator superfamily domain containing 12(Mfsd12) | Mus musculus |
| Trmt12 | tRNA methyltranferase 12(Trmt12) | Mus musculus |
| Actr10 | ARP10 actin-related protein 10(Actr10) | Mus musculus |
| Nfrkb | nuclear factor related to kappa B binding protein(Nfrkb) | Mus musculus |
| Wdr72 | WD repeat domain 72(Wdr72) | Mus musculus |
| Fopnl | Fgfr1op N-terminal like(Fopnl) | Mus musculus |
| Adar | adenosine deaminase, RNA-specific(Adar) | Mus musculus |
| Carf | calcium response factor(Carf) | Mus musculus |
| Megf10 | multiple EGF-like-domains 10(Megf10) | Mus musculus |
| Ppp4c | protein phosphatase 4, catalytic subunit(Ppp4c) | Mus musculus |
| Dab1 | disabled 1(Dab1) | Mus musculus |
| Rpa1 | replication protein A1(Rpa1) | Mus musculus |
| Rab30 | RAB30, member RAS oncogene family(Rab30) | Mus musculus |
| Ackr2 | atypical chemokine receptor 2(Ackr2) | Mus musculus |
| Cwc15 | CWC15 spliceosome-associated protein(Cwc15) | Mus musculus |
| Tomm70a | translocase of outer mitochondrial membrane 70 homolog A (yeast)(Tomm70a) | Mus musculus |
| Gramd2 | GRAM domain containing 2(Gramd2) | Mus musculus |
| Tmem170b | transmembrane protein 170B(Tmem170b) | Mus musculus |
| Arhgef40 | Rho guanine nucleotide exchange factor (GEF) 40(Arhgef40) | Mus musculus |
| Lrp11 | low density lipoprotein receptor-related protein 11(Lrp11) | Mus musculus |
| Tox4 | TOX high mobility group box family member 4(Tox4) | Mus musculus |
| Trub2 | TruB pseudouridine (psi) synthase family member 2(Trub2) | Mus musculus |
| Stx5a | syntaxin 5A(Stx5a) | Mus musculus |
| Poldip3 | polymerase (DNA-directed), delta interacting protein 3(Poldip3) | Mus musculus |
| Fam96a | family with sequence similarity 96, member A(Fam96a) | Mus musculus |
| Unc13d | unc-13 homolog D (C. elegans)(Unc13d) | Mus musculus |
| Nfat5 | nuclear factor of activated T cells 5(Nfat5) | Mus musculus |
| C330011M18Rik | RIKEN cDNA C330011M18 gene(C330011M18Rik) | Mus musculus |
| Lmx1a | LIM homeobox transcription factor 1 alpha(Lmx1a) | Mus musculus |
| Asah2 | N-acylsphingosine amidohydrolase 2(Asah2) | Mus musculus |
| A830005F24Rik | RIKEN cDNA A830005F24 gene(A830005F24Rik) | Mus musculus |
| Rps6ka3 | ribosomal protein S6 kinase polypeptide 3(Rps6ka3) | Mus musculus |
| S100a14 | S100 calcium binding protein A14(S100a14) | Mus musculus |
| Fut10 | fucosyltransferase 10(Fut10) | Mus musculus |
| Meis2 | Meis homeobox 2(Meis2) | Mus musculus |
| Cd40lg | CD40 ligand(Cd40lg) | Mus musculus |

| | | |
|---|---|---|
| Zfp383 | zinc finger protein 383(Zfp383) | Mus musculus |
| Aldh1a7 | aldehyde dehydrogenase family 1, subfamily A7(Aldh1a7) | Mus musculus |
| Nyap2 | neuronal tyrosine-phophorylated phosphoinositide 3-kinase adaptor 2(Nyap2) | Mus musculus |
| Tmem174 | transmembrane protein 174(Tmem174) | Mus musculus |
| Ets1 | E26 avian leukemia oncogene 1, 5' domain(Ets1) | Mus musculus |
| Rnf182 | ring finger protein 182(Rnf182) | Mus musculus |
| Zfp599 | zinc finger protein 599(Zfp599) | Mus musculus |
| Dmtf1 | cyclin D binding myb-like transcription factor 1(Dmtf1) | Mus musculus |
| Zfp810 | zinc finger protein 810(Zfp810) | Mus musculus |
| Anapc11 | anaphase promoting complex subunit 11(Anapc11) | Mus musculus |
| Bace1 | beta-site APP cleaving enzyme 1(Bace1) | Mus musculus |
| Zfp426 | zinc finger protein 426(Zfp426) | Mus musculus |
| Hivep2 | human immunodeficiency virus type I enhancer binding protein 2(Hivep2) | Mus musculus |
| Pknox2 | Pbx/knotted 1 homeobox 2(Pknox2) | Mus musculus |
| Fbxo41 | F-box protein 41(Fbxo41) | Mus musculus |
| Bbx | bobby sox homolog (Drosophila)(Bbx) | Mus musculus |
| Ywhab | tyrosine 3-monooxygenase/tryptophan 5-monooxygenase activation protein, beta polypept | Mus musculus |
| Snx14 | sorting nexin 14(Snx14) | Mus musculus |
| Abcb9 | ATP-binding cassette, sub-family B (MDR/TAP), member 9(Abcb9) | Mus musculus |
| E2f8 | E2F transcription factor 8(E2f8) | Mus musculus |
| Cdc6 | cell division cycle 6(Cdc6) | Mus musculus |
| Ssx2ip | synovial sarcoma, X breakpoint 2 interacting protein(Ssx2ip) | Mus musculus |
| Nhp2 | NHP2 ribonucleoprotein(Nhp2) | Mus musculus |
| Rabl6 | RAB, member RAS oncogene family-like 6(Rabl6) | Mus musculus |
| Bpnt1 | bisphosphate 3'-nucleotidase 1(Bpnt1) | Mus musculus |
| Cyp2c37 | cytochrome P450, family 2. subfamily c, polypeptide 37(Cyp2c37) | Mus musculus |
| Tlr1 | toll-like receptor 1(Tlr1) | Mus musculus |
| Ncaph2 | non-SMC condensin II complex, subunit H2(Ncaph2) | Mus musculus |
| Vmn2r34 | vomeronasal 2, receptor 34(Vmn2r34) | Mus musculus |
| Kdm3b | KDM3B lysine (K)-specific demethylase 3B(Kdm3b) | Mus musculus |
| Tle3 | transducin-like enhancer of split 3(Tle3) | Mus musculus |
| Cadm4 | cell adhesion molecule 4(Cadm4) | Mus musculus |
| Pcca | propionyl-Coenzyme A carboxylase, alpha polypeptide(Pcca) | Mus musculus |
| Ythdc1 | YTH domain containing 1(Ythdc1) | Mus musculus |
| 4930568D16Rik | RIKEN cDNA 4930568D16 gene(4930568D16Rik) | Mus musculus |
| Rbm46 | RNA binding motif protein 46(Rbm46) | Mus musculus |
| Onecut2 | one cut domain, family member 2(Onecut2) | Mus musculus |
| Rel | reticuloendotheliosis oncogene(Rel) | Mus musculus |
| Hells | helicase, lymphoid specific(Hells) | Mus musculus |

| | | |
|---|---|---|
| BC024978 | cDNA sequence BC024978(BC024978) | Mus musculus |
| Kcnd3 | potassium voltage-gated channel, Shal-related family, member 3(Kcnd3) | Mus musculus |
| Cdc42bpb | CDC42 binding protein kinase beta(Cdc42bpb) | Mus musculus |
| Cnpy2 | canopy FGF signaling regulator 2(Cnpy2) | Mus musculus |
| Rgs17 | regulator of G-protein signaling 17(Rgs17) | Mus musculus |
| Srsf1 | serine/arginine-rich splicing factor 1(Srsf1) | Mus musculus |
| Rnf219 | ring finger protein 219(Rnf219) | Mus musculus |
| Tshz1 | teashirt zinc finger family member 1(Tshz1) | Mus musculus |
| Reg3a | regenerating islet-derived 3 alpha(Reg3a) | Mus musculus |
| Cpne1 | copine I(Cpne1) | Mus musculus |
| Zc3h11a | zinc finger CCCH type containing 11A(Zc3h11a) | Mus musculus |
| Haus2 | HAUS augmin-like complex, subunit 2(Haus2) | Mus musculus |
| Defb1 | defensin beta 1(Defb1) | Mus musculus |
| Ralgps2 | Ral GEF with PH domain and SH3 binding motif 2(Ralgps2) | Mus musculus |
| Ddx3x | DEAD/H (Asp-Glu-Ala-Asp/His) box polypeptide 3, X-linked(Ddx3x) | Mus musculus |
| Adgre4 | adhesion G protein-coupled receptor E4(Adgre4) | Mus musculus |
| Elf4 | E74-like factor 4 (ets domain transcription factor)(Elf4) | Mus musculus |
| Ddx5 | DEAD (Asp-Glu-Ala-Asp) box polypeptide 5(Ddx5) | Mus musculus |
| Cnksr3 | Cnksr family member 3(Cnksr3) | Mus musculus |
| Ddx4 | DEAD (Asp-Glu-Ala-Asp) box polypeptide 4(Ddx4) | Mus musculus |
| Ssfa2 | sperm specific antigen 2(Ssfa2) | Mus musculus |
| Msantd2 | Myb/SANT-like DNA-binding domain containing 2(Msantd2) | Mus musculus |
| Zbtb20 | zinc finger and BTB domain containing 20(Zbtb20) | Mus musculus |
| Esyt2 | extended synaptotagmin-like protein 2(Esyt2) | Mus musculus |
| Plpp7 | phospholipid phosphatase 7 (inactive)(Plpp7) | Mus musculus |
| Tgfa | transforming growth factor alpha(Tgfa) | Mus musculus |
| Fbxo33 | F-box protein 33(Fbxo33) | Mus musculus |
| Wfdc8 | WAP four-disulfide core domain 8(Wfdc8) | Mus musculus |
| Dazap2 | DAZ associated protein 2(Dazap2) | Mus musculus |
| Phactr2 | phosphatase and actin regulator 2(Phactr2) | Mus musculus |
| Tra2a | transformer 2 alpha homolog (Drosophila)(Tra2a) | Mus musculus |
| Tasp1 | taspase, threonine aspartase 1(Tasp1) | Mus musculus |
| Med26 | mediator complex subunit 26(Med26) | Mus musculus |
| Lin9 | lin-9 homolog (C. elegans)(Lin9) | Mus musculus |
| Arhgap18 | Rho GTPase activating protein 18(Arhgap18) | Mus musculus |
| Nt5e | 5' nucleotidase, ecto(Nt5e) | Mus musculus |
| Zfp667 | zinc finger protein 667(Zfp667) | Mus musculus |
| Hyi | hydroxypyruvate isomerase (putative)(Hyi) | Mus musculus |
| Zfand3 | zinc finger, AN1-type domain 3(Zfand3) | Mus musculus |

| | | |
|---|---|---|
| Rbm41 | RNA binding motif protein 41(Rbm41) | Mus musculus |
| Nemf | nuclear export mediator factor(Nemf) | Mus musculus |
| Gm4841 | predicted gene 4841(Gm4841) | Mus musculus |
| Lipn | lipase, family member N(Lipn) | Mus musculus |
| Mr1 | major histocompatibility complex, class I-related(Mr1) | Mus musculus |
| Myh3 | myosin, heavy polypeptide 3, skeletal muscle, embryonic(Myh3) | Mus musculus |
| Cyp20a1 | cytochrome P450, family 20, subfamily a, polypeptide 1(Cyp20a1) | Mus musculus |
| Homer2 | homer scaffolding protein 2(Homer2) | Mus musculus |
| Wipf2 | WAS/WASL interacting protein family, member 2(Wipf2) | Mus musculus |
| Mxi1 | MAX interactor 1, dimerization protein(Mxi1) | Mus musculus |
| Adprm | ADP-ribose/CDP-alcohol diphosphatase, manganese dependent(Adprm) | Mus musculus |
| Nhlrc3 | NHL repeat containing 3(Nhlrc3) | Mus musculus |
| Rab31 | RAB31, member RAS oncogene family(Rab31) | Mus musculus |
| Zfp24 | zinc finger protein 24(Zfp24) | Mus musculus |
| Bahcc1 | BAH domain and coiled-coil containing 1(Bahcc1) | Mus musculus |
| Cpt1a | carnitine palmitoyltransferase 1a, liver(Cpt1a) | Mus musculus |
| Btbd1 | BTB (POZ) domain containing 1(Btbd1) | Mus musculus |
| Slc15a1 | solute carrier family 15 (oligopeptide transporter), member 1(Slc15a1) | Mus musculus |
| Myo10 | myosin X(Myo10) | Mus musculus |
| Adamts16 | a disintegrin-like and metallopeptidase (reprolysin type) with thrombospondin type 1 motif | Mus musculus |
| Ctu1 | cytosolic thiouridylase subunit 1(Ctu1) | Mus musculus |
| Arel1 | apoptosis resistant E3 ubiquitin protein ligase 1(Arel1) | Mus musculus |
| Vps37a | vacuolar protein sorting 37A(Vps37a) | Mus musculus |
| Creb1 | cAMP responsive element binding protein 1(Creb1) | Mus musculus |
| Nebl | nebulette(Nebl) | Mus musculus |
| Cnfn | cornifelin(Cnfn) | Mus musculus |
| Meioc | meiosis specific with coiled-coil domain(Meioc) | Mus musculus |
| 2310035C23Rik | RIKEN cDNA 2310035C23 gene(2310035C23Rik) | Mus musculus |
| Huwe1 | HECT, UBA and WWE domain containing 1(Huwe1) | Mus musculus |
| Gstk1 | glutathione S-transferase kappa 1(Gstk1) | Mus musculus |
| Olfr862 | olfactory receptor 862(Olfr862) | Mus musculus |
| Maml3 | mastermind like 3 (Drosophila)(Maml3) | Mus musculus |
| Zdhhc15 | zinc finger, DHHC domain containing 15(Zdhhc15) | Mus musculus |
| Rcn2 | reticulocalbin 2(Rcn2) | Mus musculus |
| Rsl1d1 | ribosomal L1 domain containing 1(Rsl1d1) | Mus musculus |
| Cd2bp2 | CD2 antigen (cytoplasmic tail) binding protein 2(Cd2bp2) | Mus musculus |
| Tead1 | TEA domain family member 1(Tead1) | Mus musculus |
| Zfp652 | zinc finger protein 652(Zfp652) | Mus musculus |
| Rev3l | REV3 like, DNA directed polymerase zeta catalytic subunit(Rev3l) | Mus musculus |

| | | |
|---|---|---|
| Zfp58 | zinc finger protein 58(Zfp58) | Mus musculus |
| Zfp874a | zinc finger protein 874a(Zfp874a) | Mus musculus |
| Tec | tec protein tyrosine kinase(Tec) | Mus musculus |
| Rgs2 | regulator of G-protein signaling 2(Rgs2) | Mus musculus |
| Zfp184 | zinc finger protein 184 (Kruppel-like)(Zfp184) | Mus musculus |
| Gm17359 | predicted gene, 17359(Gm17359) | Mus musculus |
| Kcnk13 | potassium channel, subfamily K, member 13(Kcnk13) | Mus musculus |
| Mup5 | major urinary protein 5(Mup5) | Mus musculus |
| Mup1 | major urinary protein 1(Mup1) | Mus musculus |
| Col5a2 | collagen, type V, alpha 2(Col5a2) | Mus musculus |
| Col6a2 | collagen, type VI, alpha 2(Col6a2) | Mus musculus |
| Kctd8 | potassium channel tetramerisation domain containing 8(Kctd8) | Mus musculus |
| Nphp3 | nephronophthisis 3 (adolescent)(Nphp3) | Mus musculus |
| Nufip2 | nuclear fragile X mental retardation protein interacting protein 2(Nufip2) | Mus musculus |
| Cdk17 | cyclin-dependent kinase 17(Cdk17) | Mus musculus |
| Nudt8 | nudix (nucleoside diphosphate linked moiety X)-type motif 8(Nudt8) | Mus musculus |
| Gramd1b | GRAM domain containing 1B(Gramd1b) | Mus musculus |
| Ahnak | AHNAK nucleoprotein (desmoyokin)(Ahnak) | Mus musculus |
| Gm94 | predicted gene 94(Gm94) | Mus musculus |
| Jph2 | junctophilin 2(Jph2) | Mus musculus |
| Nrn1 | neuritin 1(Nrn1) | Mus musculus |
| Map1b | microtubule-associated protein 1B(Map1b) | Mus musculus |
| Adamts5 | a disintegrin-like and metallopeptidase (reprolysin type) with thrombospondin type 1 motif | Mus musculus |
| Etf1 | eukaryotic translation termination factor 1(Etf1) | Mus musculus |
| Smc3 | structural maintenance of chromosomes 3(Smc3) | Mus musculus |
| Gbe1 | glucan (1,4-alpha-), branching enzyme 1(Gbe1) | Mus musculus |
| Parg | poly (ADP-ribose) glycohydrolase(Parg) | Mus musculus |
| Samd4b | sterile alpha motif domain containing 4B(Samd4b) | Mus musculus |
| Taf12 | TATA-box binding protein associated factor 12(Taf12) | Mus musculus |
| Ldoc1l | leucine zipper, down-regulated in cancer 1-like(Ldoc1l) | Mus musculus |
| Fbxo27 | F-box protein 27(Fbxo27) | Mus musculus |
| Map3k7 | mitogen-activated protein kinase kinase kinase 7(Map3k7) | Mus musculus |
| Tox | thymocyte selection-associated high mobility group box(Tox) | Mus musculus |
| Map4k2 | mitogen-activated protein kinase kinase kinase kinase 2(Map4k2) | Mus musculus |
| Map3k1 | mitogen-activated protein kinase kinase kinase 1(Map3k1) | Mus musculus |
| Btla | B and T lymphocyte associated(Btla) | Mus musculus |
| Abhd2 | abhydrolase domain containing 2(Abhd2) | Mus musculus |
| Zfp420 | zinc finger protein 420(Zfp420) | Mus musculus |
| Zfp280d | zinc finger protein 280D(Zfp280d) | Mus musculus |

| | | |
|---|---|---|
| Rpl27 | ribosomal protein L27(Rpl27) | Mus musculus |
| Pcyt1a | phosphate cytidylyltransferase 1, choline, alpha isoform(Pcyt1a) | Mus musculus |
| Zfp658 | zinc finger protein 658(Zfp658) | Mus musculus |
| Zfp719 | zinc finger protein 719(Zfp719) | Mus musculus |
| 1700017B05Rik | RIKEN cDNA 1700017B05 gene(1700017B05Rik) | Mus musculus |
| Trpm8 | transient receptor potential cation channel, subfamily M, member 8(Trpm8) | Mus musculus |
| Col6a6 | collagen, type VI, alpha 6(Col6a6) | Mus musculus |
| Stap1 | signal transducing adaptor family member 1(Stap1) | Mus musculus |
| Atp6v1h | ATPase, H+ transporting, lysosomal V1 subunit H(Atp6v1h) | Mus musculus |
| Esrrg | estrogen-related receptor gamma(Esrrg) | Mus musculus |
| Lgi2 | leucine-rich repeat LGI family, member 2(Lgi2) | Mus musculus |
| Tmem184b | transmembrane protein 184b(Tmem184b) | Mus musculus |
| Prtg | protogenin(Prtg) | Mus musculus |
| Ppp1r14c | protein phosphatase 1, regulatory (inhibitor) subunit 14c(Ppp1r14c) | Mus musculus |
| Mtf1 | metal response element binding transcription factor 1(Mtf1) | Mus musculus |
| Dclk3 | doublecortin-like kinase 3(Dclk3) | Mus musculus |
| Lrrtm3 | leucine rich repeat transmembrane neuronal 3(Lrrtm3) | Mus musculus |
| Zfp180 | zinc finger protein 180(Zfp180) | Mus musculus |
| Msi1 | musashi RNA-binding protein 1(Msi1) | Mus musculus |
| Krt80 | keratin 80(Krt80) | Mus musculus |
| Rnf14 | ring finger protein 14(Rnf14) | Mus musculus |
| Rmnd5a | required for meiotic nuclear division 5 homolog A(Rmnd5a) | Mus musculus |
| Akap9 | A kinase (PRKA) anchor protein (yotiao) 9(Akap9) | Mus musculus |
| Gss | glutathione synthetase(Gss) | Mus musculus |
| Gspt1 | G1 to S phase transition 1(Gspt1) | Mus musculus |
| Nap1l3 | nucleosome assembly protein 1-like 3(Nap1l3) | Mus musculus |
| Ccpg1 | cell cycle progression 1(Ccpg1) | Mus musculus |
| Wwc2 | WW, C2 and coiled-coil domain containing 2(Wwc2) | Mus musculus |
| Zfp260 | zinc finger protein 260(Zfp260) | Mus musculus |
| Ccdc166 | coiled-coil domain containing 166(Ccdc166) | Mus musculus |
| Zfp146 | zinc finger protein 146(Zfp146) | Mus musculus |
| Rock1 | Rho-associated coiled-coil containing protein kinase 1(Rock1) | Mus musculus |
| Zkscan8 | zinc finger with KRAB and SCAN domains 8(Zkscan8) | Mus musculus |
| Nuak1 | NUAK family, SNF1-like kinase, 1(Nuak1) | Mus musculus |
| Susd6 | sushi domain containing 6(Susd6) | Mus musculus |
| Fam90a1a | family with sequence similarity 90, member A1A(Fam90a1a) | Mus musculus |
| Lingo1 | leucine rich repeat and Ig domain containing 1(Lingo1) | Mus musculus |
| Nhsl2 | NHS-like 2(Nhsl2) | Mus musculus |
| Clcf1 | cardiotrophin-like cytokine factor 1(Clcf1) | Mus musculus |

| | | |
|---|---|---|
| Ros1 | Ros1 proto-oncogene(Ros1) | Mus musculus |
| Ammecr1l | AMME chromosomal region gene 1-like(Ammecr1l) | Mus musculus |
| Cluh | clustered mitochondria (cluA/CLU1) homolog(Cluh) | Mus musculus |
| Krt71 | keratin 71(Krt71) | Mus musculus |
| Gtf2h1 | general transcription factor II H, polypeptide 1(Gtf2h1) | Mus musculus |
| Zfp644 | zinc finger protein 644(Zfp644) | Mus musculus |
| Usp7 | ubiquitin specific peptidase 7(Usp7) | Mus musculus |
| Rnf217 | ring finger protein 217(Rnf217) | Mus musculus |
| D1Ertd622e | DNA segment, Chr 1, ERATO Doi 622, expressed(D1Ertd622e) | Mus musculus |
| Scgb2b19 | secretoglobin, family 2B, member 19(Scgb2b19) | Mus musculus |
| Chka | choline kinase alpha(Chka) | Mus musculus |
| Sri | sorcin(Sri) | Mus musculus |
| Ube3c | ubiquitin protein ligase E3C(Ube3c) | Mus musculus |
| Dnajc19 | DnaJ heat shock protein family (Hsp40) member C19(Dnajc19) | Mus musculus |
| Oas1f | 2'-5' oligoadenylate synthetase 1F(Oas1f) | Mus musculus |
| Zfp715 | zinc finger protein 715(Zfp715) | Mus musculus |
| Xylb | xylulokinase homolog (H. influenzae)(Xylb) | Mus musculus |
| Rita1 | RBPJ interacting and tubulin associated 1(Rita1) | Mus musculus |
| Mark4 | MAP/microtubule affinity regulating kinase 4(Mark4) | Mus musculus |
| Gsx2 | GS homeobox 2(Gsx2) | Mus musculus |
| Tmem39b | transmembrane protein 39b(Tmem39b) | Mus musculus |
| Ddhd2 | DDHD domain containing 2(Ddhd2) | Mus musculus |
| Sirt1 | sirtuin 1(Sirt1) | Mus musculus |
| Lars2 | leucyl-tRNA synthetase, mitochondrial(Lars2) | Mus musculus |
| Chek1 | checkpoint kinase 1(Chek1) | Mus musculus |
| Trim2 | tripartite motif-containing 2(Trim2) | Mus musculus |
| Zfhx4 | zinc finger homeodomain 4(Zfhx4) | Mus musculus |
| Glipr2 | GLI pathogenesis-related 2(Glipr2) | Mus musculus |
| Kcnmb4 | potassium large conductance calcium-activated channel, subfamily M, beta member 4(Kcnm | Mus musculus |
| Pigx | phosphatidylinositol glycan anchor biosynthesis, class X(Pigx) | Mus musculus |
| Dcaf12l1 | DDB1 and CUL4 associated factor 12-like 1(Dcaf12l1) | Mus musculus |
| Anks4b | ankyrin repeat and sterile alpha motif domain containing 4B(Anks4b) | Mus musculus |
| Grin2b | glutamate receptor, ionotropic, NMDA2B (epsilon 2)(Grin2b) | Mus musculus |
| Spink8 | serine peptidase inhibitor, Kazal type 8(Spink8) | Mus musculus |
| Chd8 | chromodomain helicase DNA binding protein 8(Chd8) | Mus musculus |
| Senp1 | SUMO1/sentrin specific peptidase 1(Senp1) | Mus musculus |
| Csmd3 | CUB and Sushi multiple domains 3(Csmd3) | Mus musculus |
| Rap2c | RAP2C, member of RAS oncogene family(Rap2c) | Mus musculus |
| Gria2 | glutamate receptor, ionotropic, AMPA2 (alpha 2)(Gria2) | Mus musculus |

| | | |
|---|---|---|
| Gm7293 | glyceraldehyde-3-phosphate dehydrogenase pseudogene(Gm7293) | Mus musculus |
| Ip6k2 | inositol hexaphosphate kinase 2(Ip6k2) | Mus musculus |
| Cnot2 | CCR4-NOT transcription complex, subunit 2(Cnot2) | Mus musculus |
| Sfr1 | SWI5 dependent recombination repair 1(Sfr1) | Mus musculus |
| Wnt4 | wingless-type MMTV integration site family, member 4(Wnt4) | Mus musculus |
| Sub1 | SUB1 homolog (S. cerevisiae)(Sub1) | Mus musculus |
| Ccbe1 | collagen and calcium binding EGF domains 1(Ccbe1) | Mus musculus |
| Wnt7a | wingless-type MMTV integration site family, member 7A(Wnt7a) | Mus musculus |
| Pdc | phosducin(Pdc) | Mus musculus |
| Slc37a2 | solute carrier family 37 (glycerol-3-phosphate transporter), member 2(Slc37a2) | Mus musculus |
| Cebpz | CCAAT/enhancer binding protein zeta(Cebpz) | Mus musculus |
| Slc16a6 | solute carrier family 16 (monocarboxylic acid transporters), member 6(Slc16a6) | Mus musculus |
| Zfp286 | zinc finger protein 286(Zfp286) | Mus musculus |
| Snx2 | sorting nexin 2(Snx2) | Mus musculus |
| Uvrag | UV radiation resistance associated gene(Uvrag) | Mus musculus |
| Zmpste24 | zinc metallopeptidase, STE24(Zmpste24) | Mus musculus |
| Rps15a | ribosomal protein S15A(Rps15a) | Mus musculus |
| Unc5d | unc-5 netrin receptor D(Unc5d) | Mus musculus |
| Cstad | CSA-conditional, T cell activation-dependent protein(Cstad) | Mus musculus |
| Zbed4 | zinc finger, BED type containing 4(Zbed4) | Mus musculus |
| Zfp449 | zinc finger protein 449(Zfp449) | Mus musculus |
| Rassf3 | Ras association (RalGDS/AF-6) domain family member 3(Rassf3) | Mus musculus |
| Hibadh | 3-hydroxyisobutyrate dehydrogenase(Hibadh) | Mus musculus |
| Cdh4 | cadherin 4(Cdh4) | Mus musculus |
| Pak7 | p21 protein (Cdc42/Rac)-activated kinase 7(Pak7) | Mus musculus |
| Psat1 | phosphoserine aminotransferase 1(Psat1) | Mus musculus |
| Atp1a2 | ATPase, Na+/K+ transporting, alpha 2 polypeptide(Atp1a2) | Mus musculus |
| Lilr4b | leukocyte immunoglobulin-like receptor, subfamily B, member 4B(Lilr4b) | Mus musculus |
| Zfp418 | zinc finger protein 418(Zfp418) | Mus musculus |
| Gpsm1 | G-protein signalling modulator 1 (AGS3-like, C. elegans)(Gpsm1) | Mus musculus |
| Tnpo3 | transportin 3(Tnpo3) | Mus musculus |
| Atg7 | autophagy related 7(Atg7) | Mus musculus |
| Pdzd4 | PDZ domain containing 4(Pdzd4) | Mus musculus |
| Daam2 | dishevelled associated activator of morphogenesis 2(Daam2) | Mus musculus |
| Sbno1 | strawberry notch homolog 1 (Drosophila)(Sbno1) | Mus musculus |
| Tmprss11a | transmembrane protease, serine 11a(Tmprss11a) | Mus musculus |
| Tifab | TRAF-interacting protein with forkhead-associated domain, family member B(Tifab) | Mus musculus |
| Armc1 | armadillo repeat containing 1(Armc1) | Mus musculus |
| Fam46a | family with sequence similarity 46, member A(Fam46a) | Mus musculus |

| | | |
|---|---|---|
| Pex13 | peroxisomal biogenesis factor 13(Pex13) | Mus musculus |
| Nmral1 | NmrA-like family domain containing 1(Nmral1) | Mus musculus |
| Galk2 | galactokinase 2(Galk2) | Mus musculus |
| Tmem67 | transmembrane protein 67(Tmem67) | Mus musculus |
| Armc9 | armadillo repeat containing 9(Armc9) | Mus musculus |
| Nfib | nuclear factor I/B(Nfib) | Mus musculus |
| Esco2 | establishment of sister chromatid cohesion N-acetyltransferase 2(Esco2) | Mus musculus |
| Zfp735 | zinc finger protein 735(Zfp735) | Mus musculus |
| Gabpb2 | GA repeat binding protein, beta 2(Gabpb2) | Mus musculus |
| Vipr2 | vasoactive intestinal peptide receptor 2(Vipr2) | Mus musculus |
| Il31 | interleukin 31(Il31) | Mus musculus |
| Zdhhc8 | zinc finger, DHHC domain containing 8(Zdhhc8) | Mus musculus |
| Rbm24 | RNA binding motif protein 24(Rbm24) | Mus musculus |
| Tardbp | TAR DNA binding protein(Tardbp) | Mus musculus |
| A130051J06Rik | RIKEN cDNA A130051J0 gene(A130051J06Rik) | Mus musculus |
| Pcdhb9 | protocadherin beta 9(Pcdhb9) | Mus musculus |
| Ccr2 | chemokine (C-C motif) receptor 2(Ccr2) | Mus musculus |
| Vegfb | vascular endothelial growth factor B(Vegfb) | Mus musculus |
| Pcdhb14 | protocadherin beta 14(Pcdhb14) | Mus musculus |
| Zfp583 | zinc finger protein 583(Zfp583) | Mus musculus |
| Cmah | cytidine monophospho-N-acetylneuraminic acid hydroxylase(Cmah) | Mus musculus |
| Cdca4 | cell division cycle associated 4(Cdca4) | Mus musculus |
| Pank2 | pantothenate kinase 2(Pank2) | Mus musculus |
| Mrps25 | mitochondrial ribosomal protein S25(Mrps25) | Mus musculus |
| Baz1b | bromodomain adjacent to zinc finger domain, 1B(Baz1b) | Mus musculus |
| Wbp5 | WW domain binding protein 5(Wbp5) | Mus musculus |
| Ankrd13c | ankyrin repeat domain 13c(Ankrd13c) | Mus musculus |
| Neurod1 | neurogenic differentiation 1(Neurod1) | Mus musculus |
| Cers6 | ceramide synthase 6(Cers6) | Mus musculus |
| Gm10032 | predicted gene 10032(Gm10032) | Mus musculus |
| Dip2c | disco interacting protein 2 homolog C(Dip2c) | Mus musculus |
| Endod1 | endonuclease domain containing 1(Endod1) | Mus musculus |
| Inpp4a | inositol polyphosphate-4-phosphatase, type I(Inpp4a) | Mus musculus |
| Cacul1 | CDK2 associated, cullin domain 1(Cacul1) | Mus musculus |
| Rnf34 | ring finger protein 34(Rnf34) | Mus musculus |
| Gnao1 | guanine nucleotide binding protein, alpha O(Gnao1) | Mus musculus |
| Prdm10 | PR domain containing 10(Prdm10) | Mus musculus |
| Gclm | glutamate-cysteine ligase, modifier subunit(Gclm) | Mus musculus |
| Dhrs3 | dehydrogenase/reductase (SDR family) member 3(Dhrs3) | Mus musculus |

| | | |
|---|---|---|
| Cpsf7 | cleavage and polyadenylation specific factor 7(Cpsf7) | Mus musculus |
| Clcn1 | chloride channel, voltage-sensitive 1(Clcn1) | Mus musculus |
| Uty | ubiquitously transcribed tetratricopeptide repeat gene, Y chromosome(Uty) | Mus musculus |
| Ermp1 | endoplasmic reticulum metallopeptidase 1(Ermp1) | Mus musculus |
| Rabl3 | RAB, member RAS oncogene family-like 3(Rabl3) | Mus musculus |
| Frs2 | fibroblast growth factor receptor substrate 2(Frs2) | Mus musculus |
| Ncoa2 | nuclear receptor coactivator 2(Ncoa2) | Mus musculus |
| Trim63 | tripartite motif-containing 63(Trim63) | Mus musculus |
| Hectd2 | HECT domain containing 2(Hectd2) | Mus musculus |
| Adgrb3 | adhesion G protein-coupled receptor B3(Adgrb3) | Mus musculus |
| Usp9x | ubiquitin specific peptidase 9, X chromosome(Usp9x) | Mus musculus |
| Urgcp | upregulator of cell proliferation(Urgcp) | Mus musculus |
| Man2a2 | mannosidase 2, alpha 2(Man2a2) | Mus musculus |
| Gm12830 | predicted gene 12830(Gm12830) | Mus musculus |
| Ypel4 | yippee-like 4 (Drosophila)(Ypel4) | Mus musculus |
| Osbp | oxysterol binding protein(Osbp) | Mus musculus |
| Pcnp | PEST proteolytic signal containing nuclear protein(Pcnp) | Mus musculus |
| Myo7a | myosin VIIA(Myo7a) | Mus musculus |
| Onecut3 | one cut domain, family member 3(Onecut3) | Mus musculus |
| Ifitm10 | interferon induced transmembrane protein 10(Ifitm10) | Mus musculus |
| Fndc3b | fibronectin type III domain containing 3B(Fndc3b) | Mus musculus |
| Zfp882 | zinc finger protein 882(Zfp882) | Mus musculus |
| Glis3 | GLIS family zinc finger 3(Glis3) | Mus musculus |
| Dcaf5 | DDB1 and CUL4 associated factor 5(Dcaf5) | Mus musculus |
| Chuk | conserved helix-loop-helix ubiquitous kinase(Chuk) | Mus musculus |
| Atxn7 | ataxin 7(Atxn7) | Mus musculus |
| Ctnna1 | catenin (cadherin associated protein), alpha 1(Ctnna1) | Mus musculus |
| Fam134c | family with sequence similarity 134, member C(Fam134c) | Mus musculus |
| Runx2 | runt related transcription factor 2(Runx2) | Mus musculus |
| Frrs1 | ferric-chelate reductase 1(Frrs1) | Mus musculus |
| Soga1 | suppressor of glucose, autophagy associated 1(Soga1) | Mus musculus |
| Vps54 | VPS54 GARP complex subunit(Vps54) | Mus musculus |
| Cdc27 | cell division cycle 27(Cdc27) | Mus musculus |
| Runx1 | runt related transcription factor 1(Runx1) | Mus musculus |
| Cbln2 | cerebellin 2 precursor protein(Cbln2) | Mus musculus |
| Zbtb34 | zinc finger and BTB domain containing 34(Zbtb34) | Mus musculus |
| Cbfb | core binding factor beta(Cbfb) | Mus musculus |
| Zeb2 | zinc finger E-box binding homeobox 2(Zeb2) | Mus musculus |
| Arhgdig | Rho GDP dissociation inhibitor (GDI) gamma(Arhgdig) | Mus musculus |

| | | |
|---|---|---|
| Trpm3 | transient receptor potential cation channel, subfamily M, member 3(Trpm3) | Mus musculus |
| Eid1 | EP300 interacting inhibitor of differentiation 1(Eid1) | Mus musculus |
| Sema3a | sema domain, immunoglobulin domain (Ig), short basic domain, secreted, (semaphorin) 3A( | Mus musculus |
| 2010005H15Rik | RIKEN cDNA 2010005H15 gene(2010005H15Rik) | Mus musculus |
| Ss18 | synovial sarcoma translocation, Chromosome 18(Ss18) | Mus musculus |
| Duxbl2 | double homeobox B-like 2(Duxbl2) | Mus musculus |
| Ugt2b5 | UDP glucuronosyltransferase 2 family, polypeptide B5(Ugt2b5) | Mus musculus |
| Slc10a7 | solute carrier family 10 (sodium/bile acid cotransporter family), member 7(Slc10a7) | Mus musculus |
| Atp2b1 | ATPase, Ca++ transporting, plasma membrane 1(Atp2b1) | Mus musculus |
| Abhd17b | abhydrolase domain containing 17B(Abhd17b) | Mus musculus |
| Prdm9 | PR domain containing 9(Prdm9) | Mus musculus |
| Ccl25 | chemokine (C-C motif) ligand 25(Ccl25) | Mus musculus |
| Xlr5b | X-linked lymphocyte-regulated 5B(Xlr5b) | Mus musculus |
| Cd276 | CD276 antigen(Cd276) | Mus musculus |
| Unc5c | unc-5 netrin receptor C(Unc5c) | Mus musculus |
| Spdye4b | speedy/RINGO cell cycle regulator family, member E4B(Spdye4b) | Mus musculus |
| Gda | guanine deaminase(Gda) | Mus musculus |
| Trpm6 | transient receptor potential cation channel, subfamily M, member 6(Trpm6) | Mus musculus |
| Dnajc3 | DnaJ heat shock protein family (Hsp40) member C3(Dnajc3) | Mus musculus |
| Enho | energy homeostasis associated(Enho) | Mus musculus |
| D030056L22Rik | RIKEN cDNA D030056L22 gene(D030056L22Rik) | Mus musculus |
| Gm9936 | predicted gene 9936(Gm9936) | Mus musculus |
| Ubxn7 | UBX domain protein 7(Ubxn7) | Mus musculus |
| Pak2 | p21 protein (Cdc42/Rac)-activated kinase 2(Pak2) | Mus musculus |
| Cd300ld | CD300 molecule like family member d(Cd300ld) | Mus musculus |
| Hid1 | HID1 domain containing(Hid1) | Mus musculus |
| Rsrp1 | arginine/serine rich protein 1(Rsrp1) | Mus musculus |
| Chst10 | carbohydrate sulfotransferase 10(Chst10) | Mus musculus |
| Car13 | carbonic anhydrase 13(Car13) | Mus musculus |
| Fbxo42 | F-box protein 42(Fbxo42) | Mus musculus |
| Xaf1 | XIAP associated factor 1(Xaf1) | Mus musculus |
| Fam160b2 | family with sequence similarity 160, member B2(Fam160b2) | Mus musculus |
| Gm5591 | predicted gene 5591(Gm5591) | Mus musculus |
| Duxbl3 | double homeobox B-like 3(Duxbl3) | Mus musculus |
| Daam1 | dishevelled associated activator of morphogenesis 1(Daam1) | Mus musculus |
| Clasp1 | CLIP associating protein 1(Clasp1) | Mus musculus |
| Mbtps2 | membrane-bound transcription factor peptidase, site 2(Mbtps2) | Mus musculus |
| Pcdh17 | protocadherin 17(Pcdh17) | Mus musculus |
| Brinp3 | bone morphogenetic protein/retinoic acid inducible neural specific 3(Brinp3) | Mus musculus |

| | | |
|---|---|---|
| Clmn | calmin(Clmn) | Mus musculus |
| Arpc4 | actin related protein 2/3 complex, subunit 4(Arpc4) | Mus musculus |
| Atxn1 | ataxin 1(Atxn1) | Mus musculus |
| Wtap | Wilms tumour 1-associating protein(Wtap) | Mus musculus |
| Ltbp1 | latent transforming growth factor beta binding protein 1(Ltbp1) | Mus musculus |
| Ncaph | non-SMC condensin I complex, subunit H(Ncaph) | Mus musculus |
| Nelfa | negative elongation factor complex member A, Whsc2(Nelfa) | Mus musculus |
| Cnih4 | cornichon family AMPA receptor auxiliary protein 4(Cnih4) | Mus musculus |
| Iffo1 | intermediate filament family orphan 1(Iffo1) | Mus musculus |
| A730049H05Rik | RIKEN cDNA A730049H05 gene(A730049H05Rik) | Mus musculus |
| Ube2l3 | ubiquitin-conjugating enzyme E2L 3(Ube2l3) | Mus musculus |
| Patl1 | protein associated with topoisomerase II homolog 1 (yeast)(Patl1) | Mus musculus |
| Sobp | sine oculis-binding protein homolog (Drosophila)(Sobp) | Mus musculus |
| Cand1 | cullin associated and neddylation disassociated 1(Cand1) | Mus musculus |
| Pianp | PILR alpha associated neural protein(Pianp) | Mus musculus |
| Pcdh20 | protocadherin 20(Pcdh20) | Mus musculus |
| Ubfd1 | ubiquitin family domain containing 1(Ubfd1) | Mus musculus |
| Olfr20 | olfactory receptor 20(Olfr20) | Mus musculus |
| Nova1 | neuro-oncological ventral antigen 1(Nova1) | Mus musculus |
| Hs6st2 | heparan sulfate 6-O-sulfotransferase 2(Hs6st2) | Mus musculus |
| Fyttd1 | forty-two-three domain containing 1(Fyttd1) | Mus musculus |
| Ogdh | oxoglutarate (alpha-ketoglutarate) dehydrogenase (lipoamide)(Ogdh) | Mus musculus |
| Cdkl5 | cyclin-dependent kinase-like 5(Cdkl5) | Mus musculus |
| Phlpp1 | PH domain and leucine rich repeat protein phosphatase 1(Phlpp1) | Mus musculus |
| Trip11 | thyroid hormone receptor interactor 11(Trip11) | Mus musculus |
| Wdr38 | WD repeat domain 38(Wdr38) | Mus musculus |
| Rnf115 | ring finger protein 115(Rnf115) | Mus musculus |
| Srxn1 | sulfiredoxin 1 homolog (S. cerevisiae)(Srxn1) | Mus musculus |
| Fbxl21 | F-box and leucine-rich repeat protein 21(Fbxl21) | Mus musculus |
| Pcf11 | PCF11 cleavage and polyadenylation factor subunit(Pcf11) | Mus musculus |
| Ano2 | anoctamin 2(Ano2) | Mus musculus |
| Eda2r | ectodysplasin A2 receptor(Eda2r) | Mus musculus |
| Pgr15l | G protein-coupled receptor 15-like(Pgr15l) | Mus musculus |
| Dopey1 | dopey family member 1(Dopey1) | Mus musculus |
| Zak | sterile alpha motif and leucine zipper containing kinase AZK(Zak) | Mus musculus |
| Rap1b | RAS related protein 1b(Rap1b) | Mus musculus |
| Gad2 | glutamic acid decarboxylase 2(Gad2) | Mus musculus |
| Gucy1a3 | guanylate cyclase 1, soluble, alpha 3(Gucy1a3) | Mus musculus |
| Shox2 | short stature homeobox 2(Shox2) | Mus musculus |

| | | |
|---|---|---|
| Trp53inp1 | transformation related protein 53 inducible nuclear protein 1(Trp53inp1) | Mus musculus |
| Ocln | occludin(Ocln) | Mus musculus |
| Galnt3 | UDP-N-acetyl-alpha-D-galactosamine:polypeptide N-acetylgalactosaminyltransferase 3(Galr | Mus musculus |
| Mri1 | methylthioribose-1-phosphate isomerase 1(Mri1) | Mus musculus |
| Naa20 | N(alpha)-acetyltransferase 20, NatB catalytic subunit(Naa20) | Mus musculus |
| Ttn | titin(Ttn) | Mus musculus |
| Chd9 | chromodomain helicase DNA binding protein 9(Chd9) | Mus musculus |
| Jph1 | junctophilin 1(Jph1) | Mus musculus |
| Gabrb2 | gamma-aminobutyric acid (GABA) A receptor, subunit beta 2(Gabrb2) | Mus musculus |
| Ttc3 | tetratricopeptide repeat domain 3(Ttc3) | Mus musculus |
| Icosl | icos ligand(Icosl) | Mus musculus |
| Noct | nocturnin(Noct) | Mus musculus |
| Nr2c2 | nuclear receptor subfamily 2, group C, member 2(Nr2c2) | Mus musculus |
| C1galt1 | core 1 synthase, glycoprotein-N-acetylgalactosamine 3-beta-galactosyltransferase, 1(C1galt | Mus musculus |
| Trim36 | tripartite motif-containing 36(Trim36) | Mus musculus |
| Sgce | sarcoglycan, epsilon(Sgce) | Mus musculus |
| Tpp2 | tripeptidyl peptidase II(Tpp2) | Mus musculus |
| Scg5 | secretogranin V(Scg5) | Mus musculus |
| D10Wsu102e | DNA segment, Chr 10, Wayne State University 102, expressed(D10Wsu102e) | Mus musculus |
| Zfp106 | zinc finger protein 106(Zfp106) | Mus musculus |
| Pcmtd2 | protein-L-isoaspartate (D-aspartate) O-methyltransferase domain containing 2(Pcmtd2) | Mus musculus |
| Ctla4 | cytotoxic T-lymphocyte-associated protein 4(Ctla4) | Mus musculus |
| Skap1 | src family associated phosphoprotein 1(Skap1) | Mus musculus |
| Numb | numb homolog (Drosophila)(Numb) | Mus musculus |
| Mga | MAX gene associated(Mga) | Mus musculus |
| Ubn2 | ubinuclein 2(Ubn2) | Mus musculus |
| Traf6 | TNF receptor-associated factor 6(Traf6) | Mus musculus |
| Ntrk3 | neurotrophic tyrosine kinase, receptor, type 3(Ntrk3) | Mus musculus |
| Styk1 | serine/threonine/tyrosine kinase 1(Styk1) | Mus musculus |
| Shpk | sedoheptulokinase(Shpk) | Mus musculus |
| Tob1 | transducer of ErbB-2.1(Tob1) | Mus musculus |
| Snx7 | sorting nexin 7(Snx7) | Mus musculus |
| Nsdhl | NAD(P) dependent steroid dehydrogenase-like(Nsdhl) | Mus musculus |
| D330045A20Rik | RIKEN cDNA D330045A20 gene(D330045A20Rik) | Mus musculus |
| Dennd5b | DENN/MADD domain containing 5B(Dennd5b) | Mus musculus |
| Pum3 | pumilio RNA-binding family member 3(Pum3) | Mus musculus |
| S100pbp | S100P binding protein(S100pbp) | Mus musculus |
| Rab43 | RAB43, member RAS oncogene family(Rab43) | Mus musculus |
| Nrxn1 | neurexin I(Nrxn1) | Mus musculus |

| | | |
|---|---|---|
| Zfp672 | zinc finger protein 672(Zfp672) | Mus musculus |
| Cxxc4 | CXXC finger 4(Cxxc4) | Mus musculus |
| Wnk1 | WNK lysine deficient protein kinase 1(Wnk1) | Mus musculus |
| Tbck | TBC1 domain containing kinase(Tbck) | Mus musculus |
| Zfp568 | zinc finger protein 568(Zfp568) | Mus musculus |
| Zfp709 | zinc finger protein 709(Zfp709) | Mus musculus |
| Srsf10 | serine/arginine-rich splicing factor 10(Srsf10) | Mus musculus |
| Kcnj6 | potassium inwardly-rectifying channel, subfamily J, member 6(Kcnj6) | Mus musculus |
| Fat1 | FAT atypical cadherin 1(Fat1) | Mus musculus |
| Eif1 | eukaryotic translation initiation factor 1(Eif1) | Mus musculus |
| Corin | corin(Corin) | Mus musculus |
| Txndc11 | thioredoxin domain containing 11(Txndc11) | Mus musculus |
| P2rx1 | purinergic receptor P2X, ligand-gated ion channel, 1(P2rx1) | Mus musculus |
| Smoc2 | SPARC related modular calcium binding 2(Smoc2) | Mus musculus |
| Rimkla | ribosomal modification protein rimK-like family member A(Rimkla) | Mus musculus |
| Dennd1b | DENN/MADD domain containing 1B(Dennd1b) | Mus musculus |
| Atp5c1 | ATP synthase, H+ transporting, mitochondrial F1 complex, gamma polypeptide 1(Atp5c1) | Mus musculus |
| Strap | serine/threonine kinase receptor associated protein(Strap) | Mus musculus |
| Phf21a | PHD finger protein 21A(Phf21a) | Mus musculus |
| Zfp189 | zinc finger protein 189(Zfp189) | Mus musculus |
| Tshz3 | teashirt zinc finger family member 3(Tshz3) | Mus musculus |
| Sclt1 | sodium channel and clathrin linker 1(Sclt1) | Mus musculus |
| Pak3 | p21 protein (Cdc42/Rac)-activated kinase 3(Pak3) | Mus musculus |
| Gm4631 | predicted gene 4631(Gm4631) | Mus musculus |
| Hnrnpa2b1 | heterogeneous nuclear ribonucleoprotein A2/B1(Hnrnpa2b1) | Mus musculus |
| Zkscan16 | zinc finger with KRAB and SCAN domains 16(Zkscan16) | Mus musculus |
| Mtdh | metadherin(Mtdh) | Mus musculus |
| Atp1b3 | ATPase, Na+/K+ transporting, beta 3 polypeptide(Atp1b3) | Mus musculus |
| Wfikkn2 | WAP, follistatin/kazal, immunoglobulin, kunitz and netrin domain containing 2(Wfikkn2) | Mus musculus |
| Zfp850 | zinc finger protein 850(Zfp850) | Mus musculus |
| Atf2 | activating transcription factor 2(Atf2) | Mus musculus |
| Zfp563 | zinc finger protein 563(Zfp563) | Mus musculus |
| Kif5b | kinesin family member 5B(Kif5b) | Mus musculus |
| Fbrs | fibrosin(Fbrs) | Mus musculus |
| Gm5798 | predicted gene 5798(Gm5798) | Mus musculus |
| Zfp799 | zinc finger protein 799(Zfp799) | Mus musculus |
| Rnf141 | ring finger protein 141(Rnf141) | Mus musculus |
| Kif3b | kinesin family member 3B(Kif3b) | Mus musculus |
| Pak1 | p21 protein (Cdc42/Rac)-activated kinase 1(Pak1) | Mus musculus |

| | | |
|---|---|---|
| Gm10392 | predicted gene 10392(Gm10392) | Mus musculus |
| Zfp473 | zinc finger protein 473(Zfp473) | Mus musculus |
| Fgf14 | fibroblast growth factor 14(Fgf14) | Mus musculus |
| Pank3 | pantothenate kinase 3(Pank3) | Mus musculus |
| Tnfrsf22 | tumor necrosis factor receptor superfamily, member 22(Tnfrsf22) | Mus musculus |
| Zfp811 | zinc finger protein 811(Zfp811) | Mus musculus |
| Gm156 | predicted gene 156(Gm156) | Mus musculus |
| 9930022D16Rik | RIKEN cDNA 9930022D16 gene(9930022D16Rik) | Mus musculus |
| Sav1 | salvador family WW domain containing 1(Sav1) | Mus musculus |
| Gm3371 | predicted gene 3371(Gm3371) | Mus musculus |
| Tubgcp3 | tubulin, gamma complex associated protein 3(Tubgcp3) | Mus musculus |
| Fer | fer (fms/fps related) protein kinase(Fer) | Mus musculus |
| Duxbl1 | double homeobox B-like 1(Duxbl1) | Mus musculus |
| Slc16a13 | solute carrier family 16 (monocarboxylic acid transporters), member 13(Slc16a13) | Mus musculus |
| Boc | biregional cell adhesion molecule-related/down-regulated by oncogenes (Cdon) binding pro | Mus musculus |
| Ipcef1 | interaction protein for cytohesin exchange factors 1(Ipcef1) | Mus musculus |
| Lancl3 | LanC lantibiotic synthetase component C-like 3 (bacterial)(Lancl3) | Mus musculus |
| Timm21 | tranlocase of inner mitochondrial membrane 21(Timm21) | Mus musculus |
| Klf3 | Kruppel-like factor 3 (basic)(Klf3) | Mus musculus |
| Iqub | IQ motif and ubiquitin domain containing(Iqub) | Mus musculus |
| Atrn | attractin(Atrn) | Mus musculus |
| Cdk16 | cyclin-dependent kinase 16(Cdk16) | Mus musculus |
| Pcsk5 | proprotein convertase subtilisin/kexin type 5(Pcsk5) | Mus musculus |
| Zfp397 | zinc finger protein 397(Zfp397) | Mus musculus |
| Aak1 | AP2 associated kinase 1(Aak1) | Mus musculus |
| Fhl1 | four and a half LIM domains 1(Fhl1) | Mus musculus |
| Mgat5 | mannoside acetylglucosaminyltransferase 5(Mgat5) | Mus musculus |
| Arid2 | AT rich interactive domain 2 (ARID, RFX-like)(Arid2) | Mus musculus |
| 1700019D03Rik | RIKEN cDNA 1700019D03 gene(1700019D03Rik) | Mus musculus |
| Stx6 | syntaxin 6(Stx6) | Mus musculus |
| Fgl2 | fibrinogen-like protein 2(Fgl2) | Mus musculus |
| P4ha3 | procollagen-proline, 2-oxoglutarate 4-dioxygenase (proline 4-hydroxylase), alpha polypeptic | Mus musculus |
| Rgag4 | retrotransposon gag domain containing 4(Rgag4) | Mus musculus |
| Cyp2c54 | cytochrome P450, family 2, subfamily c, polypeptide 54(Cyp2c54) | Mus musculus |
| Fgfr1 | fibroblast growth factor receptor 1(Fgfr1) | Mus musculus |
| Surf4 | surfeit gene 4(Surf4) | Mus musculus |
| Fkbp7 | FK506 binding protein 7(Fkbp7) | Mus musculus |
| Tmem260 | transmembrane protein 260(Tmem260) | Mus musculus |
| Zfp746 | zinc finger protein 746(Zfp746) | Mus musculus |

| | | |
|---|---|---|
| 2410141K09Rik | RIKEN cDNA 2410141K09 gene(2410141K09Rik) | Mus musculus |
| E330009J07Rik | RIKEN cDNA E330009J07 gene(E330009J07Rik) | Mus musculus |
| Pde6d | phosphodiesterase 6D, cGMP-specific, rod, delta(Pde6d) | Mus musculus |
| Pde7a | phosphodiesterase 7A(Pde7a) | Mus musculus |
| Fmo6 | flavin containing monooxygenase 6(Fmo6) | Mus musculus |
| Cacna1h | calcium channel, voltage-dependent, T type, alpha 1H subunit(Cacna1h) | Mus musculus |
| Kdm4c | lysine (K)-specific demethylase 4C(Kdm4c) | Mus musculus |
| Itgae | integrin alpha E, epithelial-associated(Itgae) | Mus musculus |
| Il10 | interleukin 10(Il10) | Mus musculus |
| Scaf4 | SR-related CTD-associated factor 4(Scaf4) | Mus musculus |
| Zfp949 | zinc finger protein 949(Zfp949) | Mus musculus |
| Rasgef1b | RasGEF domain family, member 1B(Rasgef1b) | Mus musculus |
| Sh3pxd2a | SH3 and PX domains 2A(Sh3pxd2a) | Mus musculus |
| Ctgf | connective tissue growth factor(Ctgf) | Mus musculus |
| Apoa2 | apolipoprotein A-II(Apoa2) | Mus musculus |
| Gm4847 | predicted gene 4847(Gm4847) | Mus musculus |
| Rnf224 | ring finger protein 224(Rnf224) | Mus musculus |
| Cd47 | CD47 antigen (Rh-related antigen, integrin-associated signal transducer)(Cd47) | Mus musculus |
| Whsc1l1 | Wolf-Hirschhorn syndrome candidate 1-like 1 (human)(Whsc1l1) | Mus musculus |
| Parp6 | poly (ADP-ribose) polymerase family, member 6(Parp6) | Mus musculus |
| Peg3 | paternally expressed 3(Peg3) | Mus musculus |
| Vstm2b | V-set and transmembrane domain containing 2B(Vstm2b) | Mus musculus |
| Spopl | speckle-type POZ protein-like(Spopl) | Mus musculus |
| Fam69a | family with sequence similarity 69, member A(Fam69a) | Mus musculus |
| Mapk1ip1l | mitogen-activated protein kinase 1 interacting protein 1-like(Mapk1ip1l) | Mus musculus |
| Fgd5 | FYVE, RhoGEF and PH domain containing 5(Fgd5) | Mus musculus |
| B020011L13Rik | RIKEN cDNA B020011L13 gene(B020011L13Rik) | Mus musculus |
| Luzp1 | leucine zipper protein 1(Luzp1) | Mus musculus |
| Flt1 | FMS-like tyrosine kinase 1(Flt1) | Mus musculus |
| Ndufa10 | NADH dehydrogenase (ubiquinone) 1 alpha subcomplex 10(Ndufa10) | Mus musculus |
| Arhgap44 | Rho GTPase activating protein 44(Arhgap44) | Mus musculus |
| Strbp | spermatid perinuclear RNA binding protein(Strbp) | Mus musculus |
| Fam78b | family with sequence similarity 78, member B(Fam78b) | Mus musculus |
| Dgkk | diacylglycerol kinase kappa(Dgkk) | Mus musculus |
| Wbp11 | WW domain binding protein 11(Wbp11) | Mus musculus |
| Cap2 | CAP, adenylate cyclase-associated protein, 2 (yeast)(Cap2) | Mus musculus |
| Bcorl1 | BCL6 co-repressor-like 1(Bcorl1) | Mus musculus |
| 4930474N05Rik | RIKEN cDNA 4930474N05 gene(4930474N05Rik) | Mus musculus |
| Rgs20 | regulator of G-protein signaling 20(Rgs20) | Mus musculus |

| | | |
|---|---|---|
| Rap1a | RAS-related protein-1a(Rap1a) | Mus musculus |
| Tbc1d19 | TBC1 domain family, member 19(Tbc1d19) | Mus musculus |
| Stc1 | stanniocalcin 1(Stc1) | Mus musculus |
| Khdc1a | KH domain containing 1A(Khdc1a) | Mus musculus |
| Gm8011 | predicted gene 8011(Gm8011) | Mus musculus |
| Cdk14 | cyclin-dependent kinase 14(Cdk14) | Mus musculus |
| Jarid2 | jumonji, AT rich interactive domain 2(Jarid2) | Mus musculus |
| Fign | fidgetin(Fign) | Mus musculus |
| Per1 | period circadian clock 1(Per1) | Mus musculus |
| 4930503E14Rik | RIKEN cDNA 4930503E14 gene(4930503E14Rik) | Mus musculus |
| Arntl | aryl hydrocarbon receptor nuclear translocator-like(Arntl) | Mus musculus |
| Celf4 | CUGBP, Elav-like family member 4(Celf4) | Mus musculus |
| Tmem251 | transmembrane protein 251(Tmem251) | Mus musculus |
| Kbtbd7 | kelch repeat and BTB (POZ) domain containing 7(Kbtbd7) | Mus musculus |
| Kcna2 | potassium voltage-gated channel, shaker-related subfamily, member 2(Kcna2) | Mus musculus |
| Arnt | aryl hydrocarbon receptor nuclear translocator(Arnt) | Mus musculus |
| Tmprss12 | transmembrane (C-terminal) protease, serine 12(Tmprss12) | Mus musculus |
| Gps1 | G protein pathway suppressor 1(Gps1) | Mus musculus |
| Cpne9 | copine family member IX(Cpne9) | Mus musculus |
| Snx4 | sorting nexin 4(Snx4) | Mus musculus |
| Kcnb1 | potassium voltage gated channel, Shab-related subfamily, member 1(Kcnb1) | Mus musculus |
| Arf3 | ADP-ribosylation factor 3(Arf3) | Mus musculus |
| Fstl1 | follistatin-like 1(Fstl1) | Mus musculus |
| Stpg2 | sperm tail PG rich repeat containing 2(Stpg2) | Mus musculus |
| Fst | follistatin(Fst) | Mus musculus |
| Amot | angiomotin(Amot) | Mus musculus |
| Ssr3 | signal sequence receptor, gamma(Ssr3) | Mus musculus |
| Ctdspl2 | CTD (carboxy-terminal domain, RNA polymerase II, polypeptide A) small phosphatase like 2( | Mus musculus |
| Epb42 | erythrocyte membrane protein band 4.2(Epb42) | Mus musculus |
| Gm7233 | predicted gene 7233(Gm7233) | Mus musculus |
| 4930503B20Rik | RIKEN cDNA 4930503B20 gene(4930503B20Rik) | Mus musculus |
| Chic1 | cysteine-rich hydrophobic domain 1(Chic1) | Mus musculus |
| Pik3r1 | phosphatidylinositol 3-kinase, regulatory subunit, polypeptide 1 (p85 alpha)(Pik3r1) | Mus musculus |
| Gbp7 | guanylate binding protein 7(Gbp7) | Mus musculus |
| Cadm2 | cell adhesion molecule 2(Cadm2) | Mus musculus |
| Igf1r | insulin-like growth factor I receptor(Igf1r) | Mus musculus |
| Sox1 | SRY (sex determining region Y)-box 1(Sox1) | Mus musculus |
| Ephb2 | Eph receptor B2(Ephb2) | Mus musculus |
| Sort1 | sortilin 1(Sort1) | Mus musculus |

| | | |
|---|---|---|
| Sorl1 | sortilin-related receptor, LDLR class A repeats-containing(Sorl1) | Mus musculus |
| Zyg11b | zyg-ll family member B, cell cycle regulator(Zyg11b) | Mus musculus |
| Dsg4 | desmoglein 4(Dsg4) | Mus musculus |
| Epha7 | Eph receptor A7(Epha7) | Mus musculus |
| Pim1 | proviral integration site 1(Pim1) | Mus musculus |
| Pcgf3 | polycomb group ring finger 3(Pcgf3) | Mus musculus |
| Erbb4 | erb-b2 receptor tyrosine kinase 4(Erbb4) | Mus musculus |
| Ldb2 | LIM domain binding 2(Ldb2) | Mus musculus |
| Zfp763 | zinc finger protein 763(Zfp763) | Mus musculus |
| Lcp2 | lymphocyte cytosolic protein 2(Lcp2) | Mus musculus |
| Maob | monoamine oxidase B(Maob) | Mus musculus |
| Col16a1 | collagen, type XVI, alpha 1(Col16a1) | Mus musculus |
| Erp29 | endoplasmic reticulum protein 29(Erp29) | Mus musculus |
| Scgb2b12 | secretoglobin, family 2B, member 12(Scgb2b12) | Mus musculus |
| Rnf38 | ring finger protein 38(Rnf38) | Mus musculus |
| Prkce | protein kinase C, epsilon(Prkce) | Mus musculus |
| Ccdc171 | coiled-coil domain containing 171(Ccdc171) | Mus musculus |
| Exoc8 | exocyst complex component 8(Exoc8) | Mus musculus |
| 1700034J05Rik | RIKEN cDNA 1700034J05 gene(1700034J05Rik) | Mus musculus |
| Rnf103 | ring finger protein 103(Rnf103) | Mus musculus |
| Slc39a2 | solute carrier family 39 (zinc transporter), member 2(Slc39a2) | Mus musculus |
| Cdk13 | cyclin-dependent kinase 13(Cdk13) | Mus musculus |
| Ywhaz | tyrosine 3-monooxygenase/tryptophan 5-monooxygenase activation protein, zeta polypept | Mus musculus |
| Ankrd52 | ankyrin repeat domain 52(Ankrd52) | Mus musculus |
| Pgm3 | phosphoglucomutase 3(Pgm3) | Mus musculus |
| Jchain | immunoglobulin joining chain(Jchain) | Mus musculus |
| Pla2r1 | phospholipase A2 receptor 1(Pla2r1) | Mus musculus |
| Lypla1 | lysophospholipase 1(Lypla1) | Mus musculus |
| Ldhb | lactate dehydrogenase B(Ldhb) | Mus musculus |
| Scn4b | sodium channel, type IV, beta(Scn4b) | Mus musculus |
| Mob3b | MOB kinase activator 3B(Mob3b) | Mus musculus |
| Btg1 | B cell translocation gene 1, anti-proliferative(Btg1) | Mus musculus |
| Papola | poly (A) polymerase alpha(Papola) | Mus musculus |
| Ip6k1 | inositol hexaphosphate kinase 1(Ip6k1) | Mus musculus |
| Plaur | plasminogen activator, urokinase receptor(Plaur) | Mus musculus |
| Nol4l | nucleolar protein 4-like(Nol4l) | Mus musculus |
| Siae | sialic acid acetylesterase(Siae) | Mus musculus |
| Zfp160 | zinc finger protein 160(Zfp160) | Mus musculus |
| Sp4 | trans-acting transcription factor 4(Sp4) | Mus musculus |

| | | |
|---|---|---|
| Arrb2 | arrestin, beta 2(Arrb2) | Mus musculus |
| Lhx2 | LIM homeobox protein 2(Lhx2) | Mus musculus |
| Zfp60 | zinc finger protein 60(Zfp60) | Mus musculus |
| Slc5a4b | solute carrier family 5 (neutral amino acid transporters, system A), member 4b(Slc5a4b) | Mus musculus |
| Zfp59 | zinc finger protein 59(Zfp59) | Mus musculus |
| Tsr2 | TSR2 20S rRNA accumulation(Tsr2) | Mus musculus |
| Zfp81 | zinc finger protein 81(Zfp81) | Mus musculus |
| Chd2 | chromodomain helicase DNA binding protein 2(Chd2) | Mus musculus |
| Gpr85 | G protein-coupled receptor 85(Gpr85) | Mus musculus |
| Zfp472 | zinc finger protein 472(Zfp472) | Mus musculus |
| Arhgap11a | Rho GTPase activating protein 11A(Arhgap11a) | Mus musculus |
| Ssbp1 | single-stranded DNA binding protein 1(Ssbp1) | Mus musculus |
| Zfp961 | zinc finger protein 961(Zfp961) | Mus musculus |
| Slc23a1 | solute carrier family 23 (nucleobase transporters), member 1(Slc23a1) | Mus musculus |
| Bdnf | brain derived neurotrophic factor(Bdnf) | Mus musculus |
| Slc3a1 | solute carrier family 3, member 1(Slc3a1) | Mus musculus |
| Klra9 | killer cell lectin-like receptor subfamily A, member 9(Klra9) | Mus musculus |
| Zfp37 | zinc finger protein 37(Zfp37) | Mus musculus |
| Zfp35 | zinc finger protein 35(Zfp35) | Mus musculus |
| Kras | Kirsten rat sarcoma viral oncogene homolog(Kras) | Mus musculus |
| Slc4a3 | solute carrier family 4 (anion exchanger), member 3(Slc4a3) | Mus musculus |
| Zfp26 | zinc finger protein 26(Zfp26) | Mus musculus |
| Kcnip2 | Kv channel-interacting protein 2(Kcnip2) | Mus musculus |
| Clhc1 | clathrin heavy chain linker domain containing 1(Clhc1) | Mus musculus |
| Gatsl2 | GATS protein-like 2(Gatsl2) | Mus musculus |
| Slbp | stem-loop binding protein(Slbp) | Mus musculus |
| Zfp398 | zinc finger protein 398(Zfp398) | Mus musculus |
| Tanc2 | tetratricopeptide repeat, ankyrin repeat and coiled-coil containing 2(Tanc2) | Mus musculus |
| Celf2 | CUGBP, Elav-like family member 2(Celf2) | Mus musculus |
| Zfp874b | zinc finger protein 874b(Zfp874b) | Mus musculus |
| Opn1sw | opsin 1 (cone pigments), short-wave-sensitive (color blindness, tritan)(Opn1sw) | Mus musculus |
| Slc12a2 | solute carrier family 12, member 2(Slc12a2) | Mus musculus |
| Nptxr | neuronal pentraxin receptor(Nptxr) | Mus musculus |
| Dusp12 | dual specificity phosphatase 12(Dusp12) | Mus musculus |
| Sfxn4 | sideroflexin 4(Sfxn4) | Mus musculus |
| Agbl1 | ATP/GTP binding protein-like 1(Agbl1) | Mus musculus |
| Nlgn1 | neuroligin 1(Nlgn1) | Mus musculus |
| Pcgf2 | polycomb group ring finger 2(Pcgf2) | Mus musculus |
| Ccdc30 | coiled-coil domain containing 30(Ccdc30) | Mus musculus |

| | | |
|---|---|---|
| Bcl2l2 | BCL2-like 2(Bcl2l2) | Mus musculus |
| Csnk1g1 | casein kinase 1, gamma 1(Csnk1g1) | Mus musculus |
| Zfand4 | zinc finger, AN1-type domain 4(Zfand4) | Mus musculus |
| Ikzf2 | IKAROS family zinc finger 2(Ikzf2) | Mus musculus |
| Insm1 | insulinoma-associated 1(Insm1) | Mus musculus |
| Rad54l2 | RAD54 like 2 (S. cerevisiae)(Rad54l2) | Mus musculus |
| Snai2 | snail family zinc finger 2(Snai2) | Mus musculus |
| Plch1 | phospholipase C, eta 1(Plch1) | Mus musculus |
| Ccdc50 | coiled-coil domain containing 50(Ccdc50) | Mus musculus |
| BC106179 | cDNA sequence BC106179(BC106179) | Mus musculus |
| Zik1 | zinc finger protein interacting with K protein 1(Zik1) | Mus musculus |
| Cnot4 | CCR4-NOT transcription complex, subunit 4(Cnot4) | Mus musculus |
| Evx1 | even-skipped homeobox 1(Evx1) | Mus musculus |
| Zfp641 | zinc finger protein 641(Zfp641) | Mus musculus |
| Clasrp | CLK4-associating serine/arginine rich protein(Clasrp) | Mus musculus |
| Hcrtr2 | hypocretin (orexin) receptor 2(Hcrtr2) | Mus musculus |
| Tmed9 | transmembrane p24 trafficking protein 9(Tmed9) | Mus musculus |
| Taok1 | TAO kinase 1(Taok1) | Mus musculus |
| Ier5 | immediate early response 5(Ier5) | Mus musculus |
| Dip2b | disco interacting protein 2 homolog B(Dip2b) | Mus musculus |
| Wbscr16 | Williams-Beuren syndrome chromosome region 16 homolog (human)(Wbscr16) | Mus musculus |
| Bend3 | BEN domain containing 3(Bend3) | Mus musculus |
| Fam214b | family with sequence similarity 214, member B(Fam214b) | Mus musculus |
| Kbtbd2 | kelch repeat and BTB (POZ) domain containing 2(Kbtbd2) | Mus musculus |
| Nrep | neuronal regeneration related protein(Nrep) | Mus musculus |
| Zfp85 | zinc finger protein 85(Zfp85) | Mus musculus |
| Pi15 | peptidase inhibitor 15(Pi15) | Mus musculus |
| Zfp9 | zinc finger protein 9(Zfp9) | Mus musculus |
| Ank2 | ankyrin 2, brain(Ank2) | Mus musculus |
| Slfn3 | schlafen 3(Slfn3) | Mus musculus |
| Ifnar2 | interferon (alpha and beta) receptor 2(Ifnar2) | Mus musculus |
| 6430550D23Rik | RIKEN cDNA 6430550D23 gene(6430550D23Rik) | Mus musculus |
| Ebf4 | early B cell factor 4(Ebf4) | Mus musculus |
| Gpbp1 | GC-rich promoter binding protein 1(Gpbp1) | Mus musculus |
| Trim44 | tripartite motif-containing 44(Trim44) | Mus musculus |
| Zfp62 | zinc finger protein 62(Zfp62) | Mus musculus |
| Il31ra | interleukin 31 receptor A(Il31ra) | Mus musculus |
| Zbtb39 | zinc finger and BTB domain containing 39(Zbtb39) | Mus musculus |
| Arrb1 | arrestin, beta 1(Arrb1) | Mus musculus |

| | | |
|---|---|---|
| Col4a6 | collagen, type IV, alpha 6(Col4a6) | Mus musculus |
| Shb | src homology 2 domain-containing transforming protein B(Shb) | Mus musculus |
| 3110043O21Rik | RIKEN cDNA 3110043O21 gene(3110043O21Rik) | Mus musculus |
| Pou3f2 | POU domain, class 3, transcription factor 2(Pou3f2) | Mus musculus |
| Etnk1 | ethanolamine kinase 1(Etnk1) | Mus musculus |
| 4933434E20Rik | RIKEN cDNA 4933434E20 gene(4933434E20Rik) | Mus musculus |
| R3hcc1l | R3H domain and coiled-coil containing 1 like(R3hcc1l) | Mus musculus |
| Phc1 | polyhomeotic-like 1 (Drosophila)(Phc1) | Mus musculus |
| Adgrf5 | adhesion G protein-coupled receptor F5(Adgrf5) | Mus musculus |
| Gm5464 | predicted gene 5464(Gm5464) | Mus musculus |
| Ddi2 | DNA-damage inducible protein 2(Ddi2) | Mus musculus |
| Edn1 | endothelin 1(Edn1) | Mus musculus |
| Pou2f1 | POU domain, class 2, transcription factor 1(Pou2f1) | Mus musculus |
| Lrrc7 | leucine rich repeat containing 7(Lrrc7) | Mus musculus |
| Eda | ectodysplasin-A(Eda) | Mus musculus |
| Gm2832 | predicted gene 2832(Gm2832) | Mus musculus |
| Xpot | exportin, tRNA (nuclear export receptor for tRNAs)(Xpot) | Mus musculus |
| Speer3 | spermatogenesis associated glutamate (E)-rich protein 3(Speer3) | Mus musculus |
| Vwc2 | von Willebrand factor C domain containing 2(Vwc2) | Mus musculus |
| Pak6 | p21 protein (Cdc42/Rac)-activated kinase 6(Pak6) | Mus musculus |
| Rsf1 | remodeling and spacing factor 1(Rsf1) | Mus musculus |
| Ldah | lipid droplet associated hydrolase(Ldah) | Mus musculus |
| Apba1 | amyloid beta (A4) precursor protein binding, family A, member 1(Apba1) | Mus musculus |
| B020004C17Rik | RIKEN cDNA B020004C17 gene(B020004C17Rik) | Mus musculus |
| Ubtf | upstream binding transcription factor, RNA polymerase I(Ubtf) | Mus musculus |
| Mapre1 | microtubule-associated protein, RP/EB family, member 1(Mapre1) | Mus musculus |
| Ebf1 | early B cell factor 1(Ebf1) | Mus musculus |
| Plppr3 | phospholipid phosphatase related 3(Plppr3) | Mus musculus |
| Vangl1 | vang-like 1 (van gogh, Drosophila)(Vangl1) | Mus musculus |
| Pnn | pinin(Pnn) | Mus musculus |
| Mier3 | MIER family member 3(Mier3) | Mus musculus |
| Zfp354a | zinc finger protein 354A(Zfp354a) | Mus musculus |
| Srp72 | signal recognition particle 72(Srp72) | Mus musculus |
| Mafk | v-maf musculoaponeurotic fibrosarcoma oncogene family, protein K (avian)(Mafk) | Mus musculus |
| Eif4g2 | eukaryotic translation initiation factor 4, gamma 2(Eif4g2) | Mus musculus |
| Fam134a | family with sequence similarity 134, member A(Fam134a) | Mus musculus |
| Eif4a2 | eukaryotic translation initiation factor 4A2(Eif4a2) | Mus musculus |
| Mtmr4 | myotubularin related protein 4(Mtmr4) | Mus musculus |
| C2cd3 | C2 calcium-dependent domain containing 3(C2cd3) | Mus musculus |

| | | |
|---|---|---|
| Thtpa | thiamine triphosphatase(Thtpa) | Mus musculus |
| Pgm2l1 | phosphoglucomutase 2-like 1(Pgm2l1) | Mus musculus |
| Eif2ak3 | eukaryotic translation initiation factor 2 alpha kinase 3(Eif2ak3) | Mus musculus |
| Zcchc16 | zinc finger, CCHC domain containing 16(Zcchc16) | Mus musculus |
| Rbak | RB-associated KRAB zinc finger(Rbak) | Mus musculus |
| Ntmt1 | N-terminal Xaa-Pro-Lys N-methyltransferase 1(Ntmt1) | Mus musculus |
| Zfp872 | zinc finger protein 872(Zfp872) | Mus musculus |
| Lyn | LYN proto-oncogene, Src family tyrosine kinase(Lyn) | Mus musculus |
| Abi3 | ABI gene family, member 3(Abi3) | Mus musculus |
| Srek1 | splicing regulatory glutamine/lysine-rich protein 1(Srek1) | Mus musculus |
| Zfp266 | zinc finger protein 266(Zfp266) | Mus musculus |
| Adam19 | a disintegrin and metallopeptidase domain 19 (meltrin beta)(Adam19) | Mus musculus |
| Marcks | myristoylated alanine rich protein kinase C substrate(Marcks) | Mus musculus |
| Efna5 | ephrin A5(Efna5) | Mus musculus |
| 5330417C22Rik | RIKEN cDNA 5330417C22 gene(5330417C22Rik) | Mus musculus |
| Prox1 | prospero homeobox 1(Prox1) | Mus musculus |
| Gm10797 | predicted gene 10797(Gm10797) | Mus musculus |
| Arhgap29 | Rho GTPase activating protein 29(Arhgap29) | Mus musculus |
| Gk5 | glycerol kinase 5 (putative)(Gk5) | Mus musculus |
| Ascc2 | activating signal cointegrator 1 complex subunit 2(Ascc2) | Mus musculus |
| Gm6793 | heterogeneous nuclear ribonucleoprotein A3 pseudogene(Gm6793) | Mus musculus |
| Necab2 | N-terminal EF-hand calcium binding protein 2(Necab2) | Mus musculus |
| Ube3b | ubiquitin protein ligase E3B(Ube3b) | Mus musculus |
| Fam172a | family with sequence similarity 172, member A(Fam172a) | Mus musculus |
| Gm10447 | predicted gene 10447(Gm10447) | Mus musculus |
| Stx8 | syntaxin 8(Stx8) | Mus musculus |
| Tmem8b | transmembrane protein 8B(Tmem8b) | Mus musculus |
| Kansl1l | KAT8 regulatory NSL complex subunit 1-like(Kansl1l) | Mus musculus |
| Zfp626 | zinc finger protein 626(Zfp626) | Mus musculus |
| Cdc42bpg | CDC42 binding protein kinase gamma (DMPK-like)(Cdc42bpg) | Mus musculus |
| Nop58 | NOP58 ribonucleoprotein(Nop58) | Mus musculus |
| B3galt2 | UDP-Gal:betaGlcNAc beta 1,3-galactosyltransferase, polypeptide 2(B3galt2) | Mus musculus |
| Snx12 | sorting nexin 12(Snx12) | Mus musculus |
| Lmnb1 | lamin B1(Lmnb1) | Mus musculus |
| Hnrnpa3 | heterogeneous nuclear ribonucleoprotein A3(Hnrnpa3) | Mus musculus |
| Smek1 | SMEK homolog 1, suppressor of mek1 (Dictyostelium)(Smek1) | Mus musculus |
| Abi1 | abl-interactor 1(Abi1) | Mus musculus |
| Glyctk | glycerate kinase(Glyctk) | Mus musculus |
| Srgn | serglycin(Srgn) | Mus musculus |

| | | |
|---|---|---|
| Esp1 | exocrine gland secreted peptide 1(Esp1) | Mus musculus |
| Cabyr | calcium-binding tyrosine-(Y)-phosphorylation regulated (fibrousheathin 2)(Cabyr) | Mus musculus |
| Hrnr | hornerin(Hrnr) | Mus musculus |
| Elf1 | E74-like factor 1(Elf1) | Mus musculus |
| Plppr4 | phospholipid phosphatase related 4(Plppr4) | Mus musculus |
| Gm7145 | predicted gene 7145(Gm7145) | Mus musculus |
| Elf5 | E74-like factor 5(Elf5) | Mus musculus |
| Gm8882 | predicted gene 8882(Gm8882) | Mus musculus |
| Henmt1 | HEN1 methyltransferase homolog 1 (Arabidopsis)(Henmt1) | Mus musculus |
| Gpr22 | G protein-coupled receptor 22(Gpr22) | Mus musculus |
| Dok4 | docking protein 4(Dok4) | Mus musculus |
| 4933402J07Rik | RIKEN cDNA 4933402J07 gene(4933402J07Rik) | Mus musculus |
| Rc3h2 | ring finger and CCCH-type zinc finger domains 2(Rc3h2) | Mus musculus |
| Xlr5a | X-linked lymphocyte-regulated 5A(Xlr5a) | Mus musculus |
| Enah | enabled homolog (Drosophila)(Enah) | Mus musculus |
| Enam | enamelin(Enam) | Mus musculus |
| Tnfrsf26 | tumor necrosis factor receptor superfamily, member 26(Tnfrsf26) | Mus musculus |
| Rab11a | RAB11A, member RAS oncogene family(Rab11a) | Mus musculus |
| Ephb1 | Eph receptor B1(Ephb1) | Mus musculus |
| Gm498 | predicted gene 498(Gm498) | Mus musculus |
| Alg10b | asparagine-linked glycosylation 10B (alpha-1,2-glucosyltransferase)(Alg10b) | Mus musculus |
| E130114P18Rik | RIKEN cDNA E130114P18 gene(E130114P18Rik) | Mus musculus |
| Zranb2 | zinc finger, RAN-binding domain containing 2(Zranb2) | Mus musculus |
| Vstm2a | V-set and transmembrane domain containing 2A(Vstm2a) | Mus musculus |
| Ankrd61 | ankyrin repeat domain 61(Ankrd61) | Mus musculus |
| Zfp846 | zinc finger protein 846(Zfp846) | Mus musculus |
| D17H6S53E | DNA segment, Chr 17, human D6S53E(D17H6S53E) | Mus musculus |
| Dgcr2 | DiGeorge syndrome critical region gene 2(Dgcr2) | Mus musculus |
| Rnf128 | ring finger protein 128(Rnf128) | Mus musculus |
| Edc3 | enhancer of mRNA decapping 3(Edc3) | Mus musculus |
| Tmsb4x | thymosin, beta 4, X chromosome(Tmsb4x) | Mus musculus |
| Ptpn12 | protein tyrosine phosphatase, non-receptor type 12(Ptpn12) | Mus musculus |
| Grm5 | glutamate receptor, metabotropic 5(Grm5) | Mus musculus |
| Egln3 | egl-9 family hypoxia-inducible factor 3(Egln3) | Mus musculus |
| Zbtb4 | zinc finger and BTB domain containing 4(Zbtb4) | Mus musculus |
| Ptpn14 | protein tyrosine phosphatase, non-receptor type 14(Ptpn14) | Mus musculus |
| Sumo2 | small ubiquitin-like modifier 2(Sumo2) | Mus musculus |
| Adamtsl3 | ADAMTS-like 3(Adamtsl3) | Mus musculus |
| Mtap | methylthioadenosine phosphorylase(Mtap) | Mus musculus |

| | | |
|---|---|---|
| Sdc2 | syndecan 2(Sdc2) | Mus musculus |
| Zfp617 | zinc finger protein 617(Zfp617) | Mus musculus |
| Ptprb | protein tyrosine phosphatase, receptor type, B(Ptprb) | Mus musculus |
| Gucy1b3 | guanylate cyclase 1, soluble, beta 3(Gucy1b3) | Mus musculus |
| Hspa1b | heat shock protein 1B(Hspa1b) | Mus musculus |
| Ptch1 | patched 1(Ptch1) | Mus musculus |
| Macrod2 | MACRO domain containing 2(Macrod2) | Mus musculus |
| Lyrm7 | LYR motif containing 7(Lyrm7) | Mus musculus |
| Plcz1 | phospholipase C, zeta 1(Plcz1) | Mus musculus |
| Pter | phosphotriesterase related(Pter) | Mus musculus |
| Cttnbp2 | cortactin binding protein 2(Cttnbp2) | Mus musculus |
| Rfesd | Rieske (Fe-S) domain containing(Rfesd) | Mus musculus |
| Zhx2 | zinc fingers and homeoboxes 2(Zhx2) | Mus musculus |
| Klrk1 | killer cell lectin-like receptor subfamily K, member 1(Klrk1) | Mus musculus |
| Gak | cyclin G associated kinase(Gak) | Mus musculus |
| Pycard | PYD and CARD domain containing(Pycard) | Mus musculus |
| Ypel5 | yippee-like 5 (Drosophila)(Ypel5) | Mus musculus |
| Spcs1 | signal peptidase complex subunit 1 homolog (S. cerevisiae)(Spcs1) | Mus musculus |
| Smim7 | small integral membrane protein 7(Smim7) | Mus musculus |
| Brdt | bromodomain, testis-specific(Brdt) | Mus musculus |
| Tmem170 | transmembrane protein 170(Tmem170) | Mus musculus |
| Ogt | O-linked N-acetylglucosamine (GlcNAc) transferase (UDP-N-acetylglucosamine:polypeptide- | Mus musculus |
| Ddx3y | DEAD (Asp-Glu-Ala-Asp) box polypeptide 3, Y-linked(Ddx3y) | Mus musculus |
| Zscan29 | zinc finger SCAN domains 29(Zscan29) | Mus musculus |
| Hmcn1 | hemicentin 1(Hmcn1) | Mus musculus |
| Hnrnpu | heterogeneous nuclear ribonucleoprotein U(Hnrnpu) | Mus musculus |
| Pcdh7 | protocadherin 7(Pcdh7) | Mus musculus |
| Fam175a | family with sequence similarity 175, member A(Fam175a) | Mus musculus |
| Anxa7 | annexin A7(Anxa7) | Mus musculus |
| Rbm8a2 | RNA binding motif protein 8A2(Rbm8a2) | Mus musculus |
| Elavl4 | ELAV (embryonic lethal, abnormal vision, Drosophila)-like 4 (Hu antigen D)(Elavl4) | Mus musculus |
| Ptprd | protein tyrosine phosphatase, receptor type, D(Ptprd) | Mus musculus |
| Rab21 | RAB21, member RAS oncogene family(Rab21) | Mus musculus |
| Dld | dihydrolipoamide dehydrogenase(Dld) | Mus musculus |
| Rbm38 | RNA binding motif protein 38(Rbm38) | Mus musculus |
| Ap1g1 | adaptor protein complex AP-1, gamma 1 subunit(Ap1g1) | Mus musculus |
| Dmd | dystrophin, muscular dystrophy(Dmd) | Mus musculus |
| Mblac2 | metallo-beta-lactamase domain containing 2(Mblac2) | Mus musculus |
| Wac | WW domain containing adaptor with coiled-coil(Wac) | Mus musculus |

| | | |
|---|---|---|
| Arfgef2 | ADP-ribosylation factor guanine nucleotide-exchange factor 2 (brefeldin A-inhibited)(Arfgef | Mus musculus |
| Zfyve27 | zinc finger, FYVE domain containing 27(Zfyve27) | Mus musculus |
| C2cd2 | C2 calcium-dependent domain containing 2(C2cd2) | Mus musculus |
| Gm15140 | predicted gene 15140(Gm15140) | Mus musculus |
| Purb | purine rich element binding protein B(Purb) | Mus musculus |
| Cilp | cartilage intermediate layer protein, nucleotide pyrophosphohydrolase(Cilp) | Mus musculus |
| St6galnac5 | ST6 (alpha-N-acetyl-neuraminyl-2,3-beta-galactosyl-1,3)-N-acetylgalactosaminide alpha-2,6 | Mus musculus |
| Ap1m2 | adaptor protein complex AP-1, mu 2 subunit(Ap1m2) | Mus musculus |
| Prpf40a | pre-mRNA processing factor 40A(Prpf40a) | Mus musculus |
| Ptbp2 | polypyrimidine tract binding protein 2(Ptbp2) | Mus musculus |
| Cnot6l | CCR4-NOT transcription complex, subunit 6-like(Cnot6l) | Mus musculus |
| Xlr5c | X-linked lymphocyte-regulated 5C(Xlr5c) | Mus musculus |
| Gtf3c1 | general transcription factor III C 1(Gtf3c1) | Mus musculus |
| Crocc2 | ciliary rootlet coiled-coil, rootletin family member 2(Crocc2) | Mus musculus |
| Vapa | vesicle-associated membrane protein, associated protein A(Vapa) | Mus musculus |
| Sbspon | somatomedin B and thrombospondin, type 1 domain containing(Sbspon) | Mus musculus |
| Matr3 | matrin 3(Matr3) | Mus musculus |
| Parp1 | poly (ADP-ribose) polymerase family, member 1(Parp1) | Mus musculus |
| Ran | RAN, member RAS oncogene family(Ran) | Mus musculus |
| Xrcc2 | X-ray repair complementing defective repair in Chinese hamster cells 2(Xrcc2) | Mus musculus |
| Ranbp2 | RAN binding protein 2(Ranbp2) | Mus musculus |
| Piezo2 | piezo-type mechanosensitive ion channel component 2(Piezo2) | Mus musculus |
| Kdm4b | lysine (K)-specific demethylase 4B(Kdm4b) | Mus musculus |
| Fam35a | family with sequence similarity 35, member A(Fam35a) | Mus musculus |
| Kctd13 | potassium channel tetramerisation domain containing 13(Kctd13) | Mus musculus |
| Nt5c1b | 5'-nucleotidase, cytosolic IB(Nt5c1b) | Mus musculus |
| Rhog | ras homolog family member G(Rhog) | Mus musculus |
| Mbd2 | methyl-CpG binding domain protein 2(Mbd2) | Mus musculus |
| Rbm25 | RNA binding motif protein 25(Rbm25) | Mus musculus |
| Arid3a | AT rich interactive domain 3A (BRIGHT-like)(Arid3a) | Mus musculus |
| Thoc1 | THO complex 1(Thoc1) | Mus musculus |
| Phrf1 | PHD and ring finger domains 1(Phrf1) | Mus musculus |
| Mbl2 | mannose-binding lectin (protein C) 2(Mbl2) | Mus musculus |
| Hoxa1 | homeobox A1(Hoxa1) | Mus musculus |
| Mbp | myelin basic protein(Mbp) | Mus musculus |
| Eif4b | eukaryotic translation initiation factor 4B(Eif4b) | Mus musculus |
| Ces1h | carboxylesterase 1H(Ces1h) | Mus musculus |
| Tspan5 | tetraspanin 5(Tspan5) | Mus musculus |
| Ptpdc1 | protein tyrosine phosphatase domain containing 1(Ptpdc1) | Mus musculus |

| | | |
|---|---|---|
| Nprl3 | nitrogen permease regulator-like 3(Nprl3) | Mus musculus |
| Mfsd7b | major facilitator superfamily domain containing 7B(Mfsd7b) | Mus musculus |
| Fam120a | family with sequence similarity 120, member A(Fam120a) | Mus musculus |
| Slco6d1 | solute carrier organic anion transporter family, member 6d1(Slco6d1) | Mus musculus |
| Zfp87 | zinc finger protein 87(Zfp87) | Mus musculus |
| Zfp521 | zinc finger protein 521(Zfp521) | Mus musculus |
| Usp25 | ubiquitin specific peptidase 25(Usp25) | Mus musculus |
| Zfp182 | zinc finger protein 182(Zfp182) | Mus musculus |
| Onecut1 | one cut domain, family member 1(Onecut1) | Mus musculus |
| Vma21 | VMA21 vacuolar H+-ATPase homolog (S. cerevisiae)(Vma21) | Mus musculus |
| Hnrnpa1 | heterogeneous nuclear ribonucleoprotein A1(Hnrnpa1) | Mus musculus |
| Rab23 | RAB23, member RAS oncogene family(Rab23) | Mus musculus |
| Tor1b | torsin family 1, member B(Tor1b) | Mus musculus |
| Slc25a53 | solute carrier family 25, member 53(Slc25a53) | Mus musculus |
| Samd10 | sterile alpha motif domain containing 10(Samd10) | Mus musculus |
| Veph1 | ventricular zone expressed PH domain-containing 1(Veph1) | Mus musculus |
| Hmga2 | high mobility group AT-hook 2(Hmga2) | Mus musculus |
| Spag9 | sperm associated antigen 9(Spag9) | Mus musculus |
| Meis1 | Meis homeobox 1(Meis1) | Mus musculus |
| Sdha | succinate dehydrogenase complex, subunit A, flavoprotein (Fp)(Sdha) | Mus musculus |
| Fam117b | family with sequence similarity 117, member B(Fam117b) | Mus musculus |
| Acan | aggrecan(Acan) | Mus musculus |
| Zfp964 | zinc finger protein 964(Zfp964) | Mus musculus |
| Ythdf3 | YTH domain family 3(Ythdf3) | Mus musculus |
| Fam188a | family with sequence similarity 188, member A(Fam188a) | Mus musculus |
| Vmn2r45 | vomeronasal 2, receptor 45(Vmn2r45) | Mus musculus |
| Hmgxb4 | HMG box domain containing 4(Hmgxb4) | Mus musculus |
| 4921507P07Rik | RIKEN cDNA 4921507P07 gene(4921507P07Rik) | Mus musculus |
| Otud7b | OTU domain containing 7B(Otud7b) | Mus musculus |
| Arhgef10l | Rho guanine nucleotide exchange factor (GEF) 10-like(Arhgef10l) | Mus musculus |
| Mef2d | myocyte enhancer factor 2D(Mef2d) | Mus musculus |
| Mecp2 | methyl CpG binding protein 2(Mecp2) | Mus musculus |
| Dsg1a | desmoglein 1 alpha(Dsg1a) | Mus musculus |
| Rpl27-ps3 | ribosomal protein L27, pseudogene 3(Rpl27-ps3) | Mus musculus |
| Zfp560 | zinc finger protein 560(Zfp560) | Mus musculus |
| Smarce1 | SWI/SNF related, matrix associated, actin dependent regulator of chromatin, subfamily e, m | Mus musculus |
| Dtna | dystrobrevin alpha(Dtna) | Mus musculus |
| Ttc26 | tetratricopeptide repeat domain 26(Ttc26) | Mus musculus |
| Gps2 | G protein pathway suppressor 2(Gps2) | Mus musculus |

| | | |
|---|---|---|
| Cd46 | CD46 antigen, complement regulatory protein(Cd46) | Mus musculus |
| Trpv3 | transient receptor potential cation channel, subfamily V, member 3(Trpv3) | Mus musculus |
| Zfp248 | zinc finger protein 248(Zfp248) | Mus musculus |
| Ggcx | gamma-glutamyl carboxylase(Ggcx) | Mus musculus |
| Fam168a | family with sequence similarity 168, member A(Fam168a) | Mus musculus |
| Zfp74 | zinc finger protein 74(Zfp74) | Mus musculus |
| Mlf2 | myeloid leukemia factor 2(Mlf2) | Mus musculus |
| Inpp5j | inositol polyphosphate 5-phosphatase J(Inpp5j) | Mus musculus |
| Hoxc9 | homeobox C9(Hoxc9) | Mus musculus |
| Yy2 | Yy2 transcription factor(Yy2) | Mus musculus |
| Obox2 | oocyte specific homeobox 2(Obox2) | Mus musculus |
| March9 | Membrane Associated Ring-CH-Type Finger 9 (March9) | Mus musculus |
| Unmapped Id | Gm42715 | |
| Unmapped Id | Gm21987 | |
| Unmapped Id | C030039L03Rik | |
| Unmapped Id | Gm28551 | |
| Unmapped Id | Gm17296 | |
| Unmapped Id | RP23-281H4.10 | |
| Unmapped Id | Gm20683 | |
| Unmapped Id | RP23-95K12.13 | |
| Unmapped Id | A330050F15Rik | |
| Unmapped Id | Gm26965 | |
| Unmapped Id | Gm15262 | |
| Unmapped Id | Gm26920 | |
| Unmapped Id | Zfp607 | |
| Unmapped Id | Gm28557 | |
| Unmapped Id | 1700049G17Rik | |
| Unmapped Id | Gm28168 | |

Mapped genes were used for pathway analysis.
